# Functional characterization of the ATOH1 molecular subtype indicates a pro-metastatic role in small cell lung cancer

**DOI:** 10.1016/j.celrep.2025.115603

**Published:** 2025-04-29

**Authors:** Alessia Catozzi, Maria Peiris Pagès, Sam Humphrey, Mitchell Revill, Derrick Morgan, Jordan Roebuck, Yitao Chen, Bethan Davies-Williams, Kevin Brennan, A.S. Md. Mukarram Hossain, Vsevolod J. Makeev, Karishma Satia, Pagona P. Sfyri, Melanie Galvin, Darryl Coles, Alice Lallo, Simon P. Pearce, Alastair Kerr, Lynsey Priest, Victoria Foy, Mathew Carter, Rebecca Caeser, Joseph M. Chan, Charles M. Rudin, Fiona Blackhall, Kristopher K. Frese, Caroline Dive, Kathryn L. Simpson

**Affiliations:** 1 SCLC Biology Group, Cancer Research UK Manchester Institute, University of Manchester, Manchester M20 4BX, UK; 2 Cancer Research UK Lung Cancer Centre of Excellence, Manchester and London, UK; 3 Cancer Research UK National Biomarker Centre, University of Manchester, Manchester M20 4BX, UK; 4 Medical Oncology, The Christie NHS Foundation Trust, Manchester M20 4BX, UK; 5 Division of Cancer Sciences, Faculty of Biology, Medicine and Health, University of Manchester, Manchester M20 4BX, UK; 6 Department of Medicine, Memorial Sloan Kettering Cancer Center, New York, NY 10065, USA

**Keywords:** small cell lung cancer, SCLC, metastasis, ATOH1, SCLC molecular subtype, CDX, CTC-derived explant models

## Abstract

Molecular subtypes of small cell lung cancer (SCLC) have been described based on differential expression of the transcription factors (TFs) *ASCL1*, *NEUROD1*, and *POU2F3* and immune-related genes. We previously reported an additional subtype based on expression of the neurogenic TF *ATOH1* within our SCLC circulating tumor cell-derived explant (CDX) model biobank. Here, we show that ATOH1 protein is detected in 7 of 81 preclinical models and 16 of 102 clinical samples of SCLC. In CDX models, ATOH1 directly regulates neurogenesis and differentiation programs, consistent with roles in normal tissues. In *ex vivo* cultures of ATOH1+ CDXs, ATOH1 is required for cell survival. *In vivo*, ATOH1 depletion slows tumor growth and suppresses liver metastasis. Our data validate ATOH1 as a *bona fide* lineage-defining TF of SCLC with cell survival and pro-metastatic functions. Further investigation exploring ATOH1-driven vulnerabilities for targeted treatment with predictive biomarkers is warranted.

## Highlights

• ATOH1 expression defines a transcriptionally distinct SCLC molecular subtype
• A minority of SCLC tumors express ATOH1, alone or in combination with ASCL1 and NEUROD1
• ChIP/RNA-seq reveals ATOH1 regulates neurogenesis and neuronal differentiation in SCLC
• ATOH1 is essential for cell survival in *ex vivo* cultures and supports metastasis *in vivo*

## Graphical abstract

**Figure:**
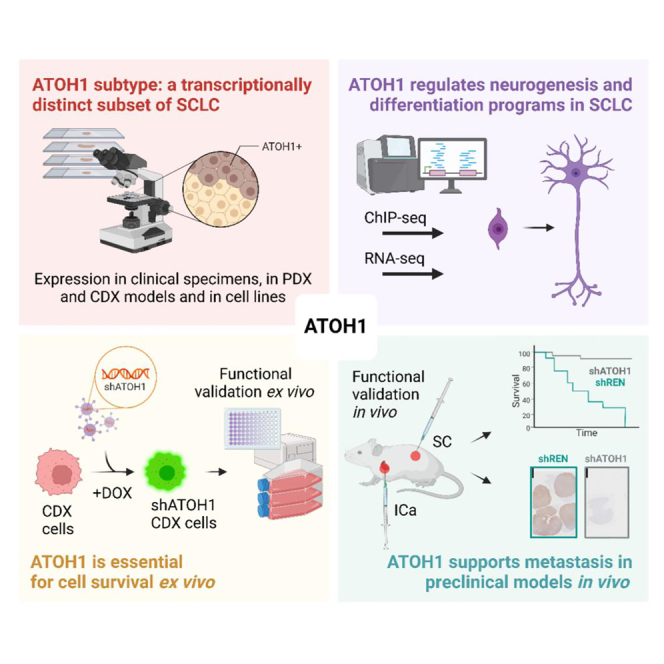

## Teaser

Catozzi et al. describe the ATOH1 SCLC molecular subtype, detected in a distinct subset of SCLC tumors and preclinical models, contributing to SCLC heterogeneity. Like ASCL1 and NEUROD1, ATOH1 regulates neurogenesis. ATOH1 is associated with chemoresistance and is essential for tumor cell survival. ATOH1 supports aggressive tumor growth, including liver metastasis.

## Introduction

Small cell lung cancer (SCLC) is an aggressive neuroendocrine (NE) tumor constituting ∼15% of lung cancers with ∼250,000 diagnoses worldwide each year and the sixth most common cause of cancer-related deaths.^1^^,^^2^^,^^3^^,^^4^ Most patients with SCLC present with extensive stage (ES) disease characterized by widespread metastases and rapidly acquired resistance to initially effective standard-of-care (SoC) platinum-based chemotherapy.^5^ The SoC was unchanged for >30 years^6^ until the recent addition of immunotherapy, which extends the overall survival of a minority of patients, including rare patients with durable responses.^7^^,^^8^^,^^9^^,^^10^

In 2019, SCLC molecular subtypes were defined based on expression of master neurogenic transcription factors (TFs) *ASCL1* (SCLC-A) and *NEUROD1* (SCLC-N) and a rarer subtype defined by the non-NE tuft cell TF *POU2F3* (SCLC-P).^11^^,^^12^ SCLC expressing an immune signature without these TFs was defined as “inflamed” (SCLC-I).^13^ Preclinical studies suggest subtype-dependent therapeutic vulnerabilities^14^ heralding potential for stratified therapy, potentially guided by circulating tumor DNA methylation subtyping,^15^ where serial liquid biopsy could assess evolving subtype plasticity.^16^

Patients with SCLC have prevalent circulating tumor cells (CTCs),^17^ prompting our establishment of CTC-derived patient explant (CDX) models in immunodeficient mice to explore SCLC biology and test novel therapeutics.^12^ ASCL1 and/or NEUROD1 subtype CDX consist primarily of NE cells with a minority non-NE subpopulation,^12^^,^^18^ consistent with NE-to-non-NE phenotype switching brought about by Notch signaling generating intra-tumoral heterogeneity.^16^^,^^19^^,^^20^ POU2F3-expressing CDX13 tumors are exclusively non-NE.^12^ YAP1, initially considered a subtype determinant of SCLC,^11^ is expressed in non-NE cells within ASCL1 or NEUROD1 CDX.^18^

We recently described a subset of SCLC CDX lacking expression of *ASCL1* or *POU2F3* that instead expressed the neurogenic, basic-helix-loop-helix TF *ATOH1*, which could be co-expressed with *NEUROD1*.^12^ *ATOH1* was expressed in 4 CDX models from 3 of 31 patients with SCLC (9.6%). Two of these CDXs were generated from the same patient pre and post treatment and maintained ATOH1 expression.

*ATOH1* is a homolog of *Drosophila melanogaster Atonal*, first identified in sensory organs of developing embryos.^21^ In mouse models, Atoh1 (or Math1) is critical for development and differentiation of sensory cell types, including granule cells in the brain, sensory inner ear hair cells, Merkel cells in the skin, and secretory cells in the intestine.^22^^,^^23^^,^^24^^,^^25^^,^^26^^,^^27^^,^^28^ Atoh1, like Ascl1, engages Notch signaling through lateral inhibition to avoid aberrant cellular differentiation in the brain and intestine.^25^^,^^29^^,^^30^ ATOH1 impact in cancer is context dependent, described as a tumor suppressor in colorectal cancer and an oncogene in medulloblastoma.^31^^,^^32^ Functional role(s) of ATOH1 in SCLC are unknown.

Although rare in our CDX biobank compared to SCLC-A, we identified ATOH1 in a subset of patient tumors and in additional patient-derived xenograft (PDX) models.^33^ We show that, in SCLC cell lines and/or CDX models, *ATOH1* regulates neurogenesis, maintains cell survival *in vitro*, and promotes tumor growth and liver metastasis *in vivo*. Our study adds to the emerging landscape of SCLC heterogeneity, highlighting potential for subtype-stratified approaches for improved treatment outcomes.

## Results

### ATOH1, MYCL, and chemosensitivity

**Figure 1:**
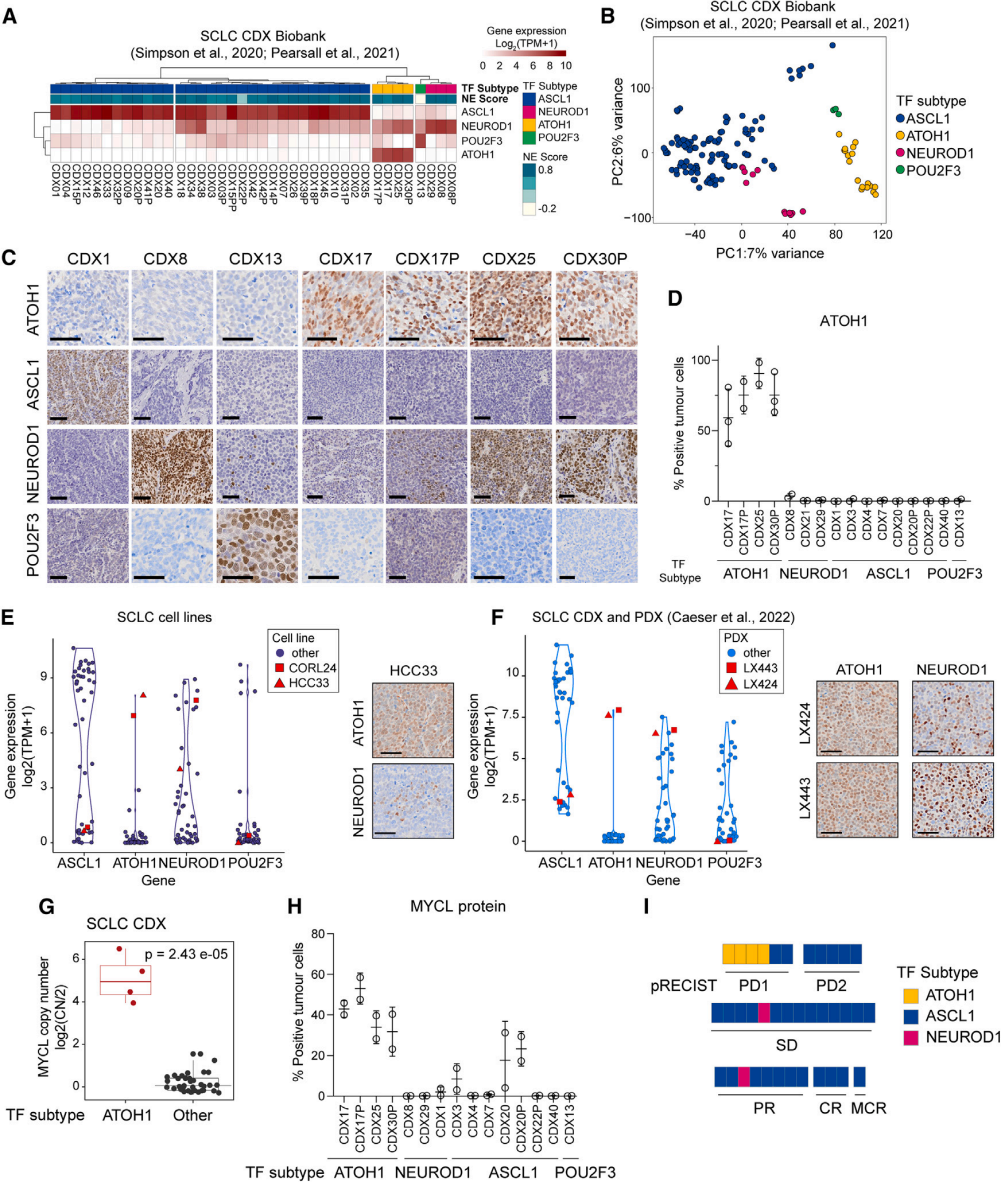
ATOH1 is expressed in a transcriptionally distinct subset of SCLC CDXs, PDXs, and established cell lines (A) Heatmap illustrating expression levels of *ASCL1*, *NEUROD1*, *ATOH1*, and *POU2F3* in the SCLC CDX biobank, annotated by SCLC subtype and NE score.^12^^,^^18^ Gene expression is shown as log_2_(transcripts per million [TPM]+1). (B) Unbiased principal-component analysis (PCA) of SCLC CDX annotated by SCLC molecular subtypes. Blue, ASCL1; pink, NEUROD1; yellow, ATOH1; green, POU2F3. (C) Representative IHC images for ATOH1, ASCL1, NEUROD1, and POU2F3 in CDX models of different SCLC molecular subtypes. Scale bars: 50 μm. (D) Quantification of ATOH1 expression in 2 CDX tumors in a panel of CDX models. Open circles show expression levels for individual biological replicates, mean value is shown with error bars representing +/-SD. (E and F) Violin plot representing expression of the indicated NE and non-NE TFs in SCLC established cell lines (E) and the SCLC CDX and PDX biobank^33^ (F); ATOH1-expressing HCC33 and CORL24 (E) and LX424 and LX443 (F) are highlighted in red. Gene expression is reported as log_2_(TPM+1). Insets: representative images of ATOH1 and NEUROD1 IHC staining for HCC33 (E) and LX424, LX443 (F). (G) Boxplot of MYCL copy number (CN), reported as CN ratio (log2(CN/2)) in CDXs grouped by molecular subtype (ATOH1 or other). Each dot represents a CDX, mean is illustrated in the box plot; statistics are reported as per Wilcoxon rank-sum exact test. (H) Quantification of MYCL expression by IHC in 2 CDX tumors in a panel of CDX models belonging to different SCLC molecular subtypes (annotated below). Open circles show expression level for individual biological replicates, mean value is shown with error bars represting +/- S.D. (I) Chemosensitivity scores of the SCLC CDX biobank according to pRECIST criteria, colored by SCLC molecular subtypes. Yellow, ATOH1; blue, ASCL1; pink, NEUROD1. Data are reported after 1 cycle of cisplatin/etoposide treatment and as average of 3 mice for 29 CDXs (STAR Methods). Statistical analysis was performed with a Fisher’s exact test between ATOH1 CDXs and the remaining CDXs; *p* = 0.0049.

We suggested ATOH1 as an SCLC subtype determinant after noting its expression in 4 of 38 CDX models that were distinct upon unsupervised clustering of whole transcriptomes^12^ (Figure 1A). Four ATOH1 CDXs were derived from three donors: one sampled prior to chemotherapy (CDX25), one post chemotherapy (CDX30P), and one where paired CDXs were generated pre and post chemotherapy (CDX17 and CDX17P) with maintained ATOH1 expression^12^ (Table S1). While ATOH1 can be co-expressed with NEUROD1 (Figure 1A), we confirmed and extended principal-component analysis (PCA) of transcriptomic data from 39 CDXs (including SCLC-A CDX31P^18^) that separated *ATOH1* models from *NEUROD1*-only models and from models expressing *ASCL1* or *POU2F3* (Figure 1B). As *ATOH1* is expressed in Merkel cells and most Merkel cell carcinomas (MCCs),^34^ we checked whether ATOH1 CDXs were, in fact, derived from CTCs from misdiagnosed MCC primary tumors characterized by oncogenic Merkel cell polyoma virus (MCPyV) (found in 80% of cases).^35^ We detected MCPyV sequences in MCC patient samples from a publicly available dataset (BioProject: PRJNA775071) but not in any ATOH1 SCLC CDXs (Figure S1A). Because a minority of MCC expresses neither *ATOH1* nor MCPyV, we performed differential gene expression analysis (DGEA) of ATOH1 CDXs compared to the entire CDX biobank and applied a Merkel cell-specific gene signature^36^ (Table S2), which was not significantly enriched in ATOH1 CDXs (Figure S1B), further supporting the theory that ATOH1 CDXs do not have a Merkel cell origin.

SCLC subtyping was based predominantly on transcriptomes.^11^^,^^13^^,^^37^ To examine ATOH1 protein expression, we optimized an immunohistochemistry (IHC) assay using a commercially available antibody (here referred to as Ptech) that revealed nuclear ATOH1 staining only in ATOH1-subtype CDXs (Figure 1C; quantified in Figure 1D). Like *ASCL1* and *POU2F3*, and in contrast to *NEUROD1*, *ATOH1* transcript and ATOH1 protein expression followed a bimodal pattern; ATOH1 was either highly expressed or undetectable (Figures 1A–1C). While ATOH1 CDXs expressed neither *ASCL1* nor *POU2F3* (Figure 1A), *ATOH1* was expressed alone (CDX17P) or in combination with *NEUROD1* at the transcript (Figure 1A) and protein levels (Figure 1C; CDX25, 78% positive tumor cells; CDX30P, 78% positive tumor cells; CDX17, moderate *NEUROD1* expression, 30% positive tumor cells). Models classified as NEUROD1 by RNA sequencing (RNA-seq) (CDX08, CDX08P, and CDX29) did not have detectable *ATOH1* expression, indicating distinct transcriptomic programs between *ATOH1* and *NEUROD1* gene expression (Figures 1A and 1B).^12^

CDXs reflect chemosensitivity profiles of their patient donors.^12^^,^^38^ We investigated tumor growth and responses of ATOH1 CDX models to the SoC (cisplatin/etoposide) *in vivo*, adopting a modified version of preclinical response evaluation criteria in solid tumors (pRECIST) (STAR Methods); tumor growth data are transformed to progressive disease (PD1 and PD2), stable disease, and partial response, complete response, and maintained complete response.^39^^,^^40^ Compared to other molecular subtype CDXs (14 ASCL1 CDXs, 4 NEUROD1 CDXs, and 4 ATOH1 CDXs), ATOH1 CDXs were the most aggressive, taking only 61 days to reach the target tumor volume of 800–1,000 mm^2^ compared to ASCL1 (75 days, *p* = 0.0128) and NEUROD1 (95 days, *p* < 0.0001) (Figure S1H). Compared to other molecular subtype CDXs (31 SCLC-A, 25 patients; 2 SCLC-N, 2 patients), which displayed variable chemotherapy responses, all 4 ATOH1 CDXs (3 patients) were the most chemoresistant, scoring as PD1 (Figure 1G; Fisher’s exact test, *p* = 0.0049; Table S1). This finding was mirrored in clinical data from the 3 ATOH1 CDX donors, who all had chemorefractory disease (Table S1). These findings were concordant with *in vitro* chemosensitivity in established SCLC cell lines, whereby the single available ATOH1-expressing HCC33 cell line was up to 10-fold more resistant to cisplatin and etoposide compared to ASCL1-and NEUROD1-expressing cell lines (Figures S1I and S1J). While more ATOH1 models are required, our early findings imply a putative association of ATOH1 with chemotherapy resistance.

**Figure 2:**
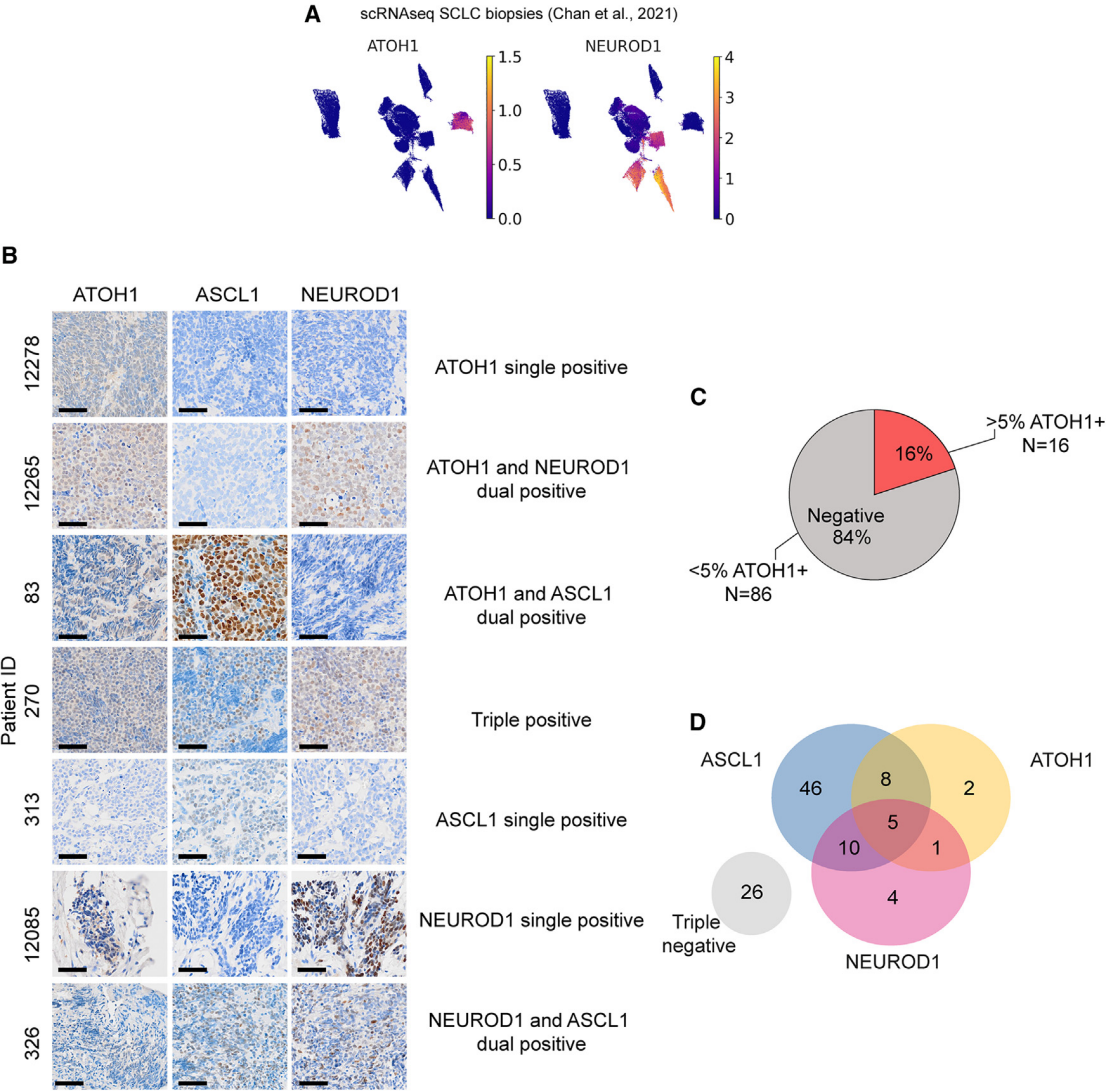
ATOH1 protein is expressed in SCLC clinical samples (A) Uniform manifold approximation and projection (UMAP) plots of single-cell RNA-seq (scRNA-seq) from SCLC biopsies from the publicly available Memorial Sloan Kettering (MSK) SCLC Atlas,^46^ reporting expression of *ATOH1* (left) and *NEUROD1* (right). Gene expression is reported in units of log_2_(X + 1), where X = normalized counts. (B) Representative IHC images for ATOH1, ASCL1, and NEUROD1 in SCLC tissue biopsies presenting with single, dual, or triple positivity (annotated). Scale bars: 50 uM. (C) Pie chart illustrating the prevalence of ATOH1+ (>5% positive tumor cells) clinical specimens (*n* = 16/102). (D) Venn diagram illustrating overlap of ASCL1, ATOH1, and NEUROD1 expression in 102 clinical specimens as detected by IHC. Positivity was determined as >1.5% positive tumor cells for ASCL1 and NEUROD1; positivity for ATOH1 was determined as in (C).

ATOH1 was expressed (transcript and protein) in 2 of 51 SCLC cell lines^41^ (Figure 1E) and 2 of 42 SCLC PDXs^33^ (Figure 1F). The ATOH1-expressing PDXs and cell lines also exhibited bimodal ATOH1 expression accompanied by either low (HCC33) or high expression of NEUROD1 (CORL24, LX424, and LX443) (Figures 1E and 1F, insets).

*MYCL* amplification is often observed in SCLC and MCC.^42^^,^^43^ *ATOH1* expression in CDXs strongly correlates with *MYCL* focal amplification (Figure 1G; *p* = 2.43 × 10^−5^), resulting in higher levels of *MYCL* transcript (Figure S1C) and MYCL protein (Figures 1H and S1D) compared to other subtypes. *ATOH1* amplification was not detected in any of the 37 CDX models tested (Figure S1E). *MYCL* amplification was also observed in *ATOH1*-expressing SCLC cell lines^44^ (HCC33 CN ratio ∼5 and CORL24 CN ratio ∼2) and PDXs (LX424/443),^33^ and all ATOH1 preclinical models expressed some of the highest reported levels of *MYCL* (Figures S1F and S1G). The *ATOH1*-expressing PDXs were obtained from one chemorefractory donor (Table S1). Overall, while requiring larger sample sizes, these findings indicate that *ATOH1* expression in SCLC CDXs, PDXs, and cell lines, with or without *NEUROD1*, correlates with high *MYCL* expression and chemoresistance.

### ATOH1 in SCLC clinical specimens

*ATOH1* was detected in 1 of 81 SCLC tumors (samples taken from diagnostic biopsies and surgical resections)^37^ and in 3 of 100 small cell NE pulmonary and extrapulmonary carcinoma biopsies.^45^ We detected *ATOH1* in 1 of 19 SCLC tumors profiled by single cell RNA-seq (scRNA-seq),^46^ previously classified as the NEUROD1 subtype by expression of *NEUROD2* and *NEUROD4* but lacking *NEUROD1* (Figure 2A). We quantified ATOH1 protein in 65 specimens from 11 LS to 54 ES patients with SCLC from the CHEMORES protocol and 37 specimens from LS patients with SCLC enrolled in the concurrent once-daily versus twice-daily chemoradiotherapy trial (STAR Methods; Table S4). ATOH1 was detected in 16 of 102 (16%) cases (Figures 2B and 2C). One patient sample co-expressed ATOH1 and NEUROD1 (1 of 16, 6%) (Figure 2D; Table S5), but in contrast to CDXs and PDXs, 8 of 16 (50%) ATOH1+ samples also had detectable ASCL1 expression, and all three neurogenic TFs were detectable in 5 of 16 (31%) cases (Figure 2D). Due to scant biopsies, we could not investigate cellular co-expression of TFs. ATOH1 expression did not correlate with altered overall survival or progression-free survival compared to other SCLC subtypes in this cohort (data not shown). Nevertheless, the relatively high prevalence of ATOH1 expression in clinical samples, either alone or combined with ASCL1 and/or NEUROD1, encouraged further study of ATOH1-driven biology.

### ATOH1 regulates a neurogenesis program by binding to E boxes at promoter and distal regulatory regions in SCLC CDXs

**Figure 3:**
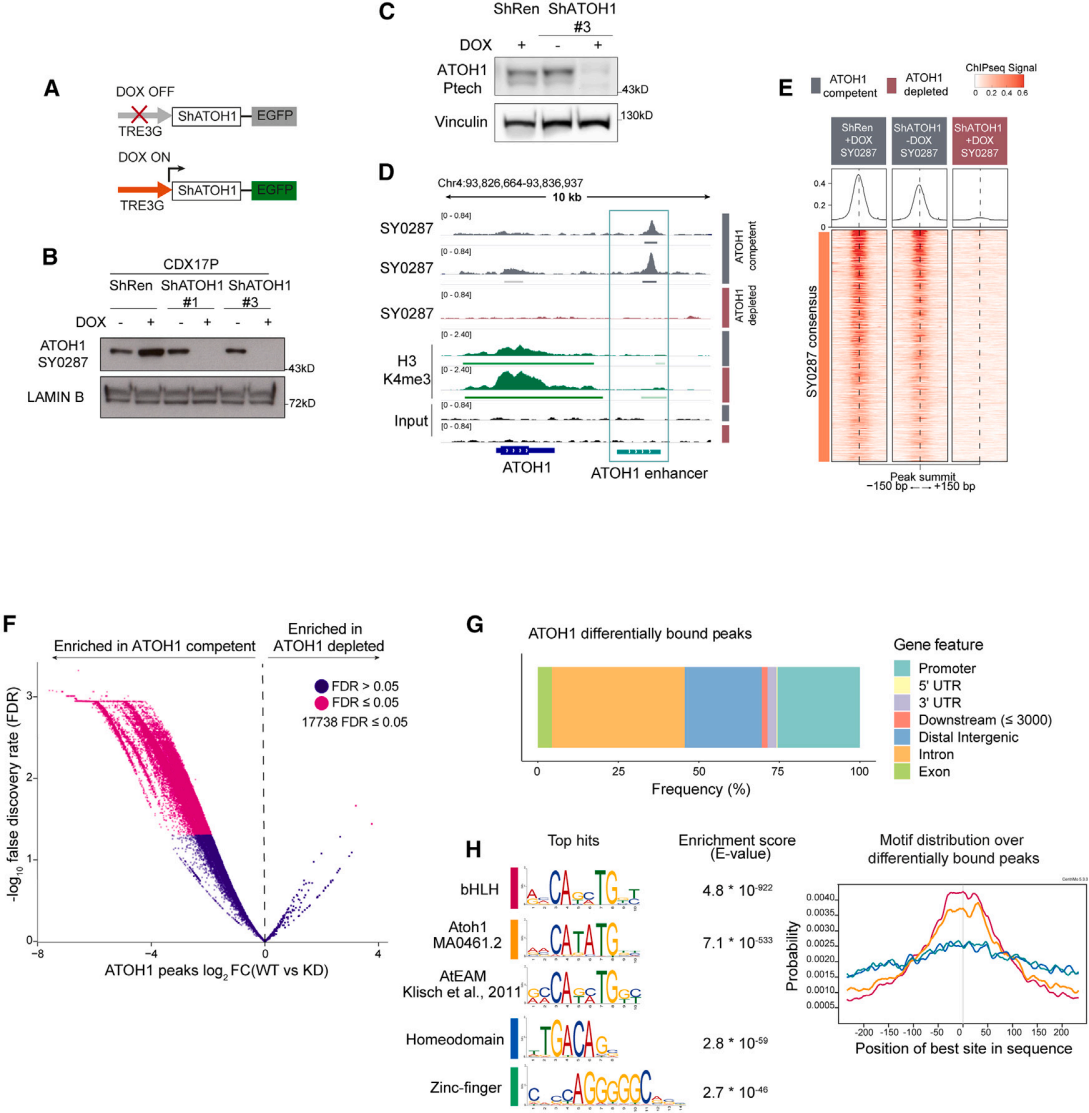
High-confidence ATOH1 binding sites are located at promoter and distal regulatory regions and are enriched for E box motifs (A) Schematic of the DOX-inducible knockdown (KD) system: without DOX, EGFP and shRNAs targeting ATOH1 (ShATOH1) or *Renilla* luciferase (ShRen) are not expressed; upon induction with DOX, both EGFP and ShATOH1 or ShRen are expressed. (B) Nuclear fractionation validating ATOH1 KD with the in-house ATOH1 antibody SY0287 in CDX17P ShRen, ShATOH1#1, and ShATOH1#3 upon treatment with DOX for 7 days. (C) Western blot showing ATOH1 expression (detected with the Ptech antibody) in the samples processed for ChIP-seq. (D) Heatmap of ChIP-seq signal for consensus peak sets SY0287 in ATOH1-competent (gray) and -depleted (red) CDX17P, generated with the generateEnrichedHeatmap function within profileplyr v.1.8.1.^48^ (E) ATOH1 binding peaks at the ATOH1 locus, highlighting ATOH1 binding peaks at the ATOH1 downstream enhancer (light green), which are lost upon ATOH1 depletion. Dark green, ChIP-seq tracks for H3K4me3 at the ATOH1 locus. Peaks were visualized with the Integrated Genomics Viewer genome browser. (F) Volcano plot of ATOH1 differentially bound regions (by false discovery rate [FDR] < 0.05) in ATOH1-competent vs. ATOH1-depleted CDX17P. Significant peaks are highlighted in pink (17,738). (G) Relative frequency of ATOH1 differentially bound peaks in regulatory genetic regions. (H) Motif enrichment analysis of ATOH1 differentially bound peaks with MEME ChIP.^49^ The mouse Atoh1 E box-associated motif (AtEAM^50^) is reported for comparison with the Atoh1 DNA binding motif and basic-helix-loop-helix (bHLH) motif. (I) Centrimo^51^ analysis of the location of enriched motifs in ATOH1 differentially bound peaks.

To interrogate biological roles of *ATOH1* in CDXs, we developed stable CDX17P lines carrying doxycycline (DOX)-inducible *ATOH1* knockdown (KD) short hairpin RNA (shRNA) constructs (ShATOH1#1 and AhATOH1#3) or a control shRNA targeting *Renilla* luciferase^47^ (ShRen) which also expressed GFP following DOX induction (Figure 3A). GFP expression enabled flow cytometry sorting of transduced cells. Maximal ATOH1 KD was observed after 7 days with both the Ptech antibody (Figure S2A) and an in-house-generated antibody (SY0287) (Figures S2B–S2E and 3B).

Transcriptional programs of *ATOH1* are unexplored in SCLC. To reveal *ATOH1*-specific TF-DNA binding, we conducted chromatin immunoprecipitation sequencing (ChIP-seq) on *ATOH1*-competent CDX17P (ShRen, 7 days of DOX and untreated ShATOH1#3) and *ATOH1*-depleted ShATOH1#3 CDX17P (7 days of DOX). Upon *ATOH1* KD (Figure 3C), samples clustered based on *ATOH1* expression (Figure S3A). While the ATOH1 ChIP-seq signal was almost completely lost upon *ATOH1* KD using SY0287 (Figure 3D), some ChIP-seq signal (∼50%) was retained with Ptech (Figure S3B), possibly due to non-specific antibody binding, consistent with immunoblots (Figures S2A and 3C). Metagene analysis showed that ATOH1 peaks were located on the transcription start site (TSS) near H3K4me3 peaks that identify active promoter regions^52^ and at intergenic regions mostly downstream of the gene body (Figure S3C), indicating that ATOH1 could regulate transcription at both promoter and distal regulatory elements. In support of this, we found that ATOH1 binds to its own enhancer, located downstream and highly conserved across species^23^ (Figures 3E and S3D).

To identify high-confidence ATOH1 binding peaks, we performed differential binding analysis between ATOH1-replete and -depleted conditions, considering peaks detected by both antibodies and thus avoiding potential false positives. We found 17,738 ATOH1-specific binding events corresponding to 70% of total peaks detected (25,464) (Figure 3F; Table S6). Among ATOH1-specific binding events, peaks are located at promoter regions (25%) and distal regulatory regions, such as distal intergenic (24%) and intronic regions (41%) (Figure 3G), in accordance with recent results from MCC lines.^53^ The most highly enriched motifs in ATOH1-specific peaks were basic-helix-loop-helix binding motifs, including the reported ATOH1 DNA binding motif (MA0461.2) and the Atoh1 E box-associated motif (AtEAM) identified in murine studies^23^^,^^50^ (Figure 3H). Compared to the second and third most enriched motifs (homeodomains and zinc fingers), E box- and ATOH1-specific motifs were found at the summit of ATOH1 peaks (Figure 3I), suggesting that they are uniquely present where there is the highest ATOH1 signal.^51^

### ATOH1 target genes in SCLC CDXs

**Figure 4:**
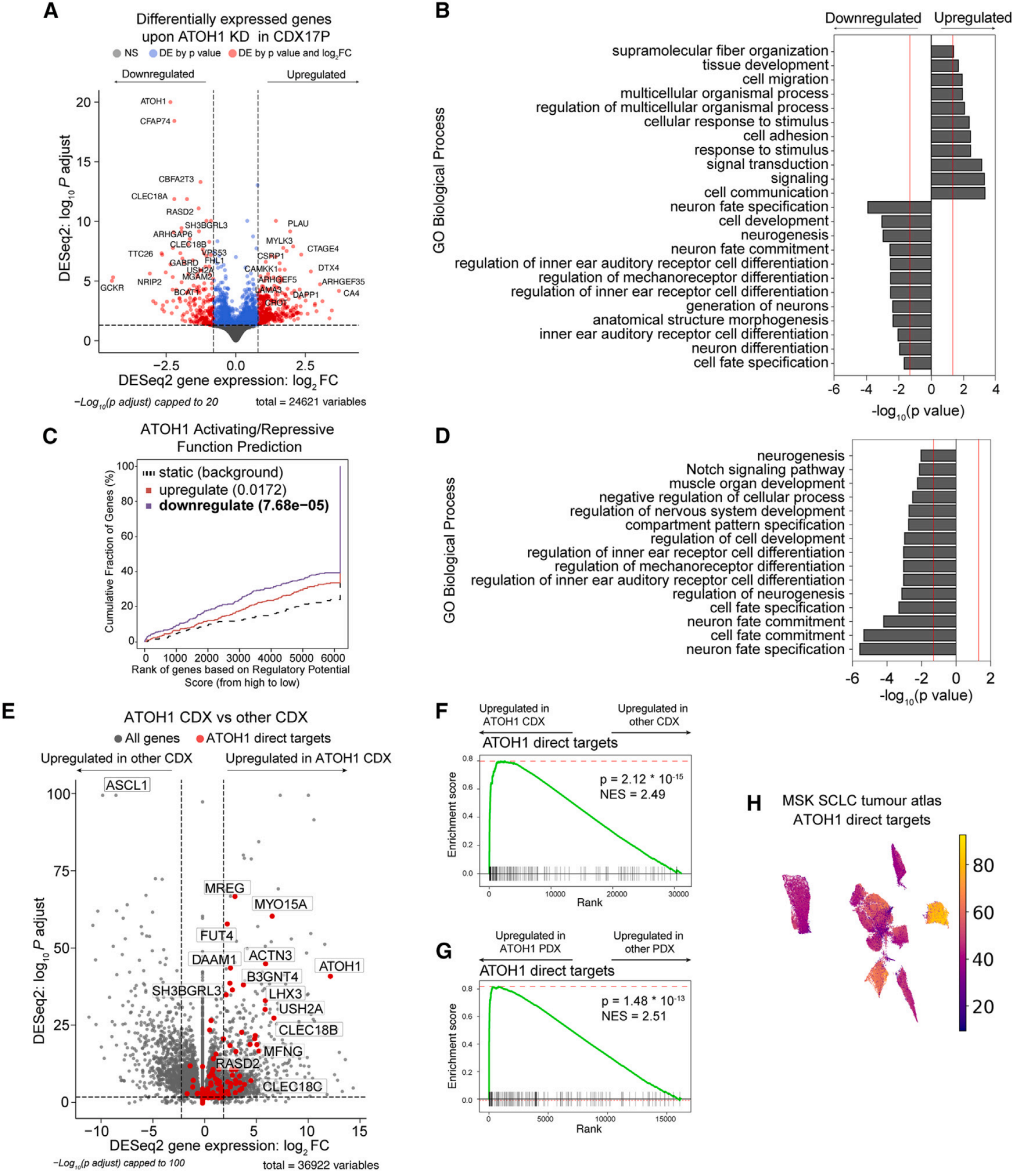
Identification of the ATOH1 targetome and gene signature (A) Volcano plot illustrating differentially expressed (DE) genes upon ATOH1 depletion (DOX treatment for 6 days) in CDX17P. Gray, not significant; blue, significant by *p* value; red, significant by *p* < 0.01 and log_2_(fold change) > 0.8 or < −0.8. Dotted lines represent thresholds for determining significant gene expression changes (*p* < 0.01 and log_2_(fold change) > 0.8 or < −0.8). The most significant DE genes are labeled. (B) Bar plot illustrating the top 20 biological processes up- and downregulated upon ATOH1 KD in CDX17P. Analysis was performed with gProfiler2.^56^ (C) Prediction of ATOH1 transcriptional function after integration of ChIP-seq and RNA-seq with BETA.^57^ ATOH1 KD results in downregulation of genes with ATOH1 binding sites identified in ChIP-seq (*p* = 7.68 × 10^−5^) and with predicted function in promoting transcription. (D) Bar plot illustrating biological processes (performed with gProfiler2) associated with ATOH1 target genes identified in (C). (E) Volcano plot illustrating genes enriched in 4 ATOH1 CDXs compared to the whole CDX biobank (*n* = 35). The ATOH1 gene signature (i.e., ATOH1 target genes) is highlighted in red. Dotted lines represent thresholds for determining significant gene expression changes (*p* < 0.01 and log_2_(fold change) > 2 or < −2). (F) Gene set enrichment analysis (GSEA) for ATOH1 direct targets in 4 ATOH1 CDXs vs. the rest of the biobank (*n* = 35). NES, normalized enrichment score. (G) GSEA for ATOH1 direct targets in 2 ATOH1 PDXs vs. the rest of the MSK PDX biobank (*n* = 40) (*p* = 1.48 × 10^−13^). GSEA was performed with Fgsea.^58^ (H) UMAP of cumulative expression of ATOH1 direct targets in scRNA-seq of SCLC tumor biopsies.^46^ ATOH1 target gene expression is highest in the only ATOH1-expressing tumor (identified in Figure 2A).

We then sought to identify the biological processes in SCLC regulated by ATOH1 and its putative target genes. Consistent with its role as a neurogenic TF, ATOH1-bound genes were enriched in pathways related to neurogenesis (Figures S3E and S3F; Table S7). However, this analysis only considered DNA binding events irrespective of gene expression changes. To define genes directly regulated by ATOH1, we performed global transcriptomics (RNA-seq) of CDX17P cells cultured *ex vivo* in the presence or absence of DOX-induced *ATOH1* KD (ShATOH#1 and ShATOH#3). Genes directly regulated by ATOH1 should be downregulated after ATOH1 loss. As expected, *ATOH1* was the most differentially expressed (DE) gene of ∼500 genes (Figure 4A; Table S8). Genes upregulated after *ATOH1* KD included those involved in cell adhesion and migration, whereas downregulated genes play roles in neurogenesis (Figure 4B; Table S9) and in inner ear hair cell differentiation, corroborated by decreased expression of independent inner ear hair cell signatures upon *ATOH1* KD^54^^,^^55^ (Figures S4A and S4B; Tables S10 and S11). Overall, our findings agree with known ATOH1 transcriptional programs in murine developmental models, where Atoh1 is required for inner ear hair cell and cerebellar granule cell development and differentiation,^22^ although the relevance of these processes to SCLC initiation and progression is unclear.

ASCL1 and NEUROD1 are highly expressed in their respective NE subtypes of SCLC,^11^^,^^59^ where they drive a NE transcriptional program. Given that ATOH1 also regulates neurogenesis, we asked whether NE status was affected by ATOH1 depletion. While a 25-gene NE signature^60^ and SYP expression were unchanged upon *ATOH1* KD (Figures S4C and S4E; Table S10), a 25-gene non-NE signature was upregulated^60^ (Figure S4D; Table S10), suggesting that ATOH1 may contribute to NE to non-NE plasticity. However, to affect a full transition, other factors may be required, such as increased expression of YAP1 or MYC, as shown in other preclinical models^16^^,^^19^ that were not evident in these data (Figure S4E).

Fewer significant transcriptional changes were seen upon *ATOH1* KD relative to the abundance of ATOH1 binding sites (by ChIP-seq), suggesting that ATOH1 activity might be restricted to a subset of ATOH1-bound genes in SCLC CDXs. Thus, to infer direct ATOH1 transcriptional targets in SCLC, we performed an integrated analysis of ChIP-seq and RNA-seq with the binding and expression target analysis (BETA).^57^ We found that ATOH1 mainly acts as a transcriptional activator (Figure 4C, blue line) and identified 150 genes downregulated upon ATOH1 depletion, directly downstream of ATOH1 (Table S12). Among these genes were components of Notch signaling (including *HES6*, *DLL1*, *DLL3*, and *DLL4*), consistent with the interplay between ATOH1 and Notch signaling during brain and intestinal development^25^^,^^61^ and genes important for inner ear hair cell development, such as *USH2A*, *LHX3*, and *RASD2*.^55^ Concordant with the ChIP-seq analysis, ATOH1 regulates expression of HES6 by binding its promoter region (Figure S4F), while it regulates LHX3 by binding multiple sites up to 60 kB downstream of the LHX3 promoter (Figure S4G). Concordant with transcriptomics analysis (Figure 4B), ATOH1 direct targets are also involved in neurogenesis and inner ear hair cell differentiation (Figure 4D; Table S13). Notably, ATOH1 direct targets minimally overlap with known ASCL1 and NEUROD1 target genes (Figures S4H and S4I; Table S14). We further validated the outcome of this analysis by mapping the top 10% ATOH1-specific binding events to their nearest genes with the Genomic Regions Enrichment of Annotations Tool ^62^^,^^63^ and then overlapping these genes with the DE genes upon ATOH1 depletion with gene set enrichment analysis (GSEA). We found a significant enrichment of ATOH1-bound genes within genes downregulated upon ATOH1 depletion (Figure S4J; normalized enrichment score [NES] = −1.46, false discovery rate [FDR] = 0%). With this information, we sought to investigate whether ATOH1 was more likely to regulate gene expression at promoters or distal regulatory elements. Thus, we divided genes mapped to the top 10% of ATOH1-specific peaks into proximally and distally regulated genes based on whether ATOH1 binding peaks were observed ±5 kb from the TSS or >5 kb from the TSS. We found that both sets of genes were significantly downregulated upon ATOH1 depletion (Figures S4K and S4L), suggesting that ATOH1 regulates gene expression both at promoters and distal regulatory elements.

This integrated analysis was performed only in CDX17P, so we next asked whether ATOH1 direct targets were conserved across all ATOH1-expressing CDXs. We performed DGEA between ATOH1 CDXs (CDX17, 17P, 25, 30P) and the whole CDX biobank (35 CDXs) (Figure 4E; Table S15), followed by GSEA for ATOH1 direct targets to demonstrate that ATOH1 direct target genes were conserved (Figure 4F; NES = 2.48, *p* = 1.13 × 10^−16^). We also detected high expression of ATOH1 target genes in the 2 ATOH1 SCLC PDXs (Figure 4G; NES = 2.44, *p* = 5 × 10^−10^) and an ATOH1-expressing tumor from the MSK SCLC tumor atlas dataset^46^ (Figure 4H). These direct targets comprise the first SCLC-based ATOH1 gene signature consistently observed in CDXs, PDXs, and tumor biopsies, indicative of a conserved transcriptional role for ATOH1 in SCLC.

### Impact of ATOH1 on SCLC CDX cell survival *ex vivo*

**Figure 5:**
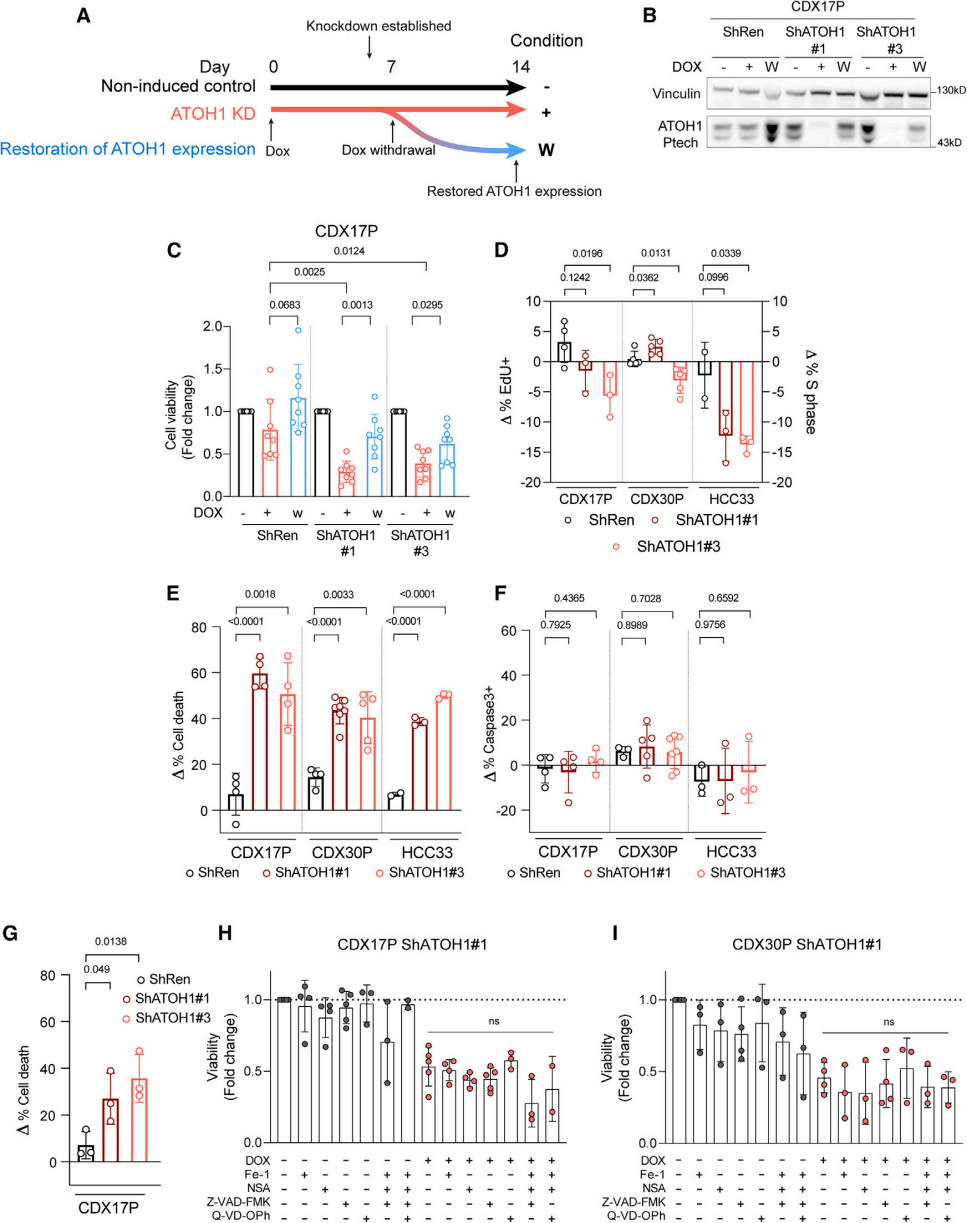
ATOH1 is necessary for SCLC cell survival *in vitro* (A) Schematic of ATOH1 KD induction. ATOH1 KD was established after 7 days of induction with 1 μg/mL doxycycline (DOX). Cells were cultured for 14 days with DOX (red line, +) or without DOX as controls; after the initial 7 days of DOX induction, an aliquot of cells was plated without DOX to restore ATOH1 expression (blue line, W). Untreated parental cells served as additional control (black line, -). (B) Western blot validation of ATOH1 depletion and restoration in the conditions specified in (A). ShRen was treated with DOX for 14 days, and untreated Sh*Ren*, ShATOH1#1, and ShATOH1#3 were used as controls. Statistics are reported as two-tailed unpaired t tests across the indicated conditions. (C) Relative cell viability measured with CellTiter-Glo (Promega) upon ATOH1 KD (red) and restoration (blue) compared to uninduced controls (black). *n* = 8 independent experiments. (D) Flow cytometry quantification of cell cycle progression using 2′-deoxy-5-ethynyluridine (EdU; CDX17P and HCC33) and propidium iodide (PI) incorporation (CDX30P). Data were normalized to DOX-untreated parental controls by subtracting the proportion of cells in S phase in untreated cells from that of DOX-treated cells (Δ % S phase = % S phase_DOX-treated_ − % S phase_untreated_); ShATOH1 conditions were compared to ShRen controls. CDX17P, *n* = 4 ShRen, *n* = 3 ShATOH1#1 and #3; CDX30P, *n* = 5; HCC33, *n* = 2 ShRen, *n* = 3 Sh*ATOH1#1* and *#3* independent experiments. (E) Flow cytometry quantification of cell death after 14 days of DOX induction of ATOH1 KD, normalized as in (D). Total cell death is reported as the sum of apoptotic and necrotic cells. CDX17P: *n* = 4; CDX30P: *n* = 4 ShRen, *n* = 7 ShATOH1#1, *n* = 5 ShATOH1#3; HCC33: *n* = 2 ShRen, *n* = 3 ShATOH1#1 and *#3* independent experiments. (F) As in (E), reporting total caspase-3+ cells. (G) Flow cytometry quantification of cell death (defined in E) after 7 days of DOX-induction of ATOH1 KD in CDX17P. *n* = 3 independent experiments. (C–G) *p* values are reported as per two-tailed unpaired t test. (H and I) ShATOH1#1 CDX17P (H) and CDX30P (I) cells were treated with (red) or without (black) DOX and with or without ferrostatin-1 (1 μM), necrosulfonamide (NSA; 100 nM), or Z-VAD-FMK/Q-VD-OPh (20 μM) and the indicated combinations for 7 days. Cell viability was measured with CellTiter-Glo, normalized to vehicle-treated, DOX-untreated cells and reported as fold change. Statistics are reported as per one-way ANOVA test with Dunnett’s test correction for multiple comparisons between DOX-treated conditions with and without programmed cell death inhibitors. Data are shown as mean ± SD.

We chose CDX17P to examine biological effects of ATOH1 depletion via DOX-inducible ATOH1 KD *ex vivo* and, subsequently, *in vivo* because of its amenability to genetic modulation and most reproducible growth properties *in vivo*. Maximal ATOH1 KD was achieved *ex vivo* after 7 days of DOX (Figure S2A) and was maintained for 14 days (longest duration of *ex vivo* studies). Withdrawal of DOX restored ATOH1 expression (7 days +DOX and then 7 days −DOX) (Figures 5A and 5B). ATOH1 depletion caused a >50% decrease in cell viability (ShATOH1#1, *p* = 0.0025; ShATOH1#3, *p* = 0.0124) compared to uninduced and ShRen controls, which was attenuated by restoring ATOH1 expression (Figure 5C). To interrogate the mechanism of decreased cell viability, we established DOX-inducible ATOH1 KD in CDX30P and HCC33 SCLC cells (Figures S5A and S5B) and assessed cell death and cell cycle progression following ATOH1 depletion. Compared to ShRen DOX-induced controls and uninduced cells, there were no reproducible changes in cell cycle progression in CDX17P or CDX30P upon ATOH1 depletion for 14 days (Figures 5D and S5C). A modest ∼12% decrease in cell proliferation was evident in HCC33 cells, although this did not constitute a complete proliferation arrest, with ∼15% cells still cycling (Figure S5D). These slightly different effects on proliferation in CDXs versus HCC33 cells may result from differences between established cell lines and plastic naive CDX *ex vivo* cultures. ATOH1 depletion increased cell death in CDX17P (55%), CDX30P (42%), and HCC33 (44%) cells after 14 days of ATOH1 depletion (Figure 5E) via a caspase-3-independent process (Figure 5F). Notably, ATOH1 depletion resulted in increased cell death in CDX30P despite increased expression of NEUROD1 (Figure S5E).

After 7 days of DOX treatment, ATOH1 KD already induced detectable cell death (Figure 5G) and a decrease in ATP production, used as a proxy for viable cell number (Figures 5H and 5I, red). Because other types of non-apoptotic, programmed cell death, such as ferroptosis and pyroptosis, have been observed in SCLC,^64^^,^^65^ we induced ATOH1 KD in CDX17P and CDX30P ShATOH1#1 with DOX and with or without cell death pathway inhibitors for 7 days. Inhibition of apoptosis, pyroptosis, necroptosis, or ferroptosis (with single or combined inhibitors) did not prevent ATOH1 KD-induced loss of cell viability (Figures 5H and 5I; Table S16). Taken together, these findings identify ATOH1 as necessary for cell survival, as its depletion induces cell death, either via an undefined programmed cell death pathway or, most likely, via necrosis.

### Impact of ATOH1 on tumor growth *in vivo*

**Figure 6:**
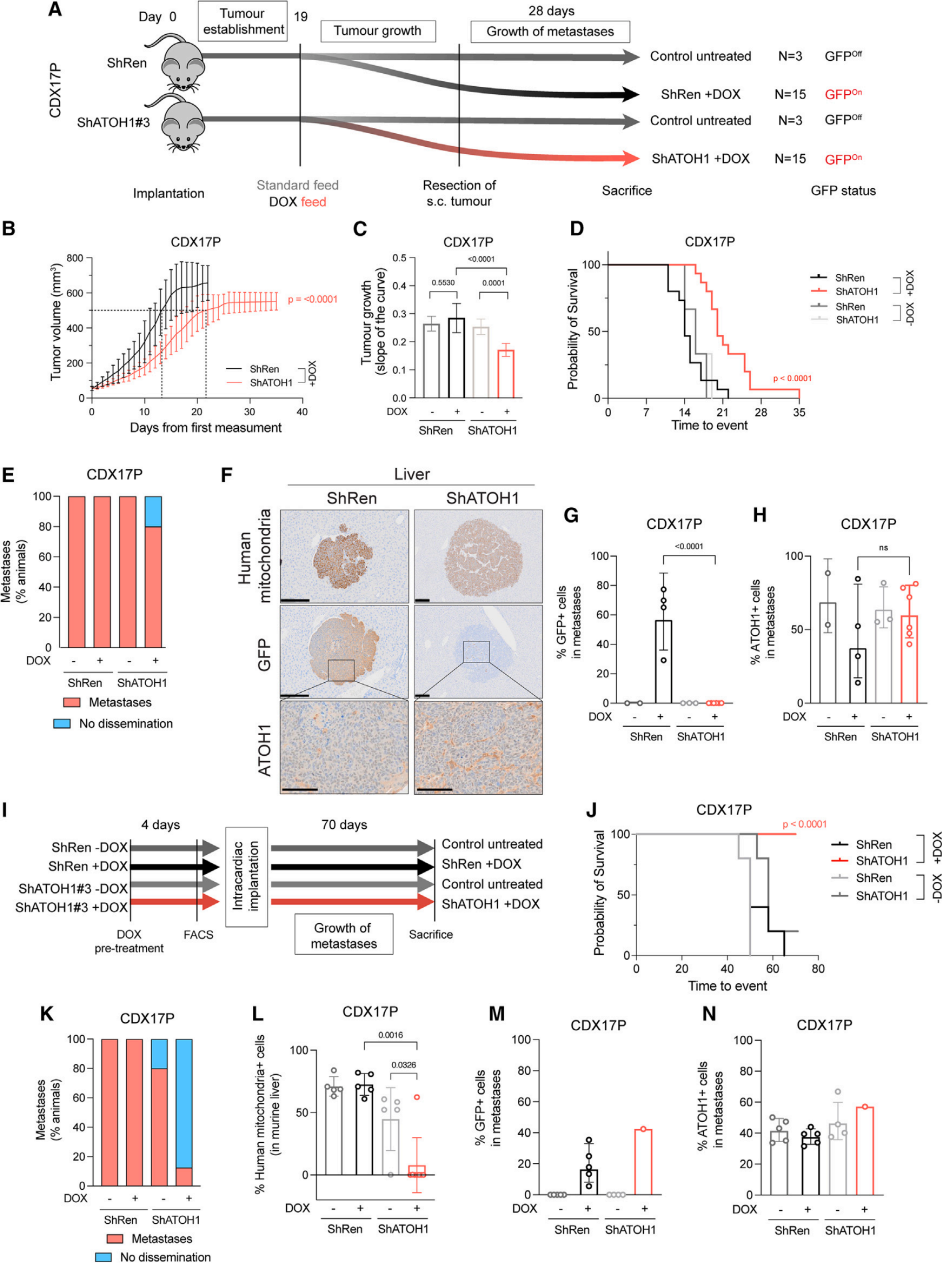
ATOH1 depletion decreases tumor growth kinetics and metastasis *in vivo* (A) *In vivo* study design to investigate subcutaneous (s.c.) tumor growth and metastasis after s.c. tumor resection. CDX17P ShRen and ShATOH1#3 (ShATOH1) were injected s.c. into NSG mice and left for 19 days for tumor establishment. Mice were then fed either a standard diet (control arms, *n* = 3) or DOX-supplemented food (experimental arms, *n* = 15), and s.c. tumor growth was assessed. Tumors were surgically resected when at 500–800 mm^3^ to allow for metastatic dissemination. Mice were kept on the study for 28 days or until s.c. tumors reached maximum size, whichever came first. (B) Tumor growth curves from day of first tumor measurement to s.c. tumor resection (STAR Methods) for mice implanted with ShRen and ShATOH1 cells and fed a DOX-supplemented diet. Black, ShRen fed a DOX diet; red, ShATOH1#3 fed a DOX diet. 15 mice per cohort; data reported as mean ± SD. Dotted lines indicate when tumors from each cohort reached 500 mm^3^: ShRen, 14 ± 3 days; ShATOH1, 21 ± 5 days. (C) Quantification of tumor growth curves slopes in (B). Shades of gray, control cohort fed a standard diet for study duration. *p* values were calculated with ANOVA test, and slope of the curve is reported as mean ± SD per cohort. (D) Kaplan-Meier curve of time to surgical resection of s.c. tumor or maximum 800 mm^3^ for inoperable tumors. Control arms, fed a standard diet, are reported in scales of gray. *p* values were calculated with log rank Mantel-Cox test. (E) Quantification of metastatic dissemination to the liver in 3 mice fed a standard diet, 5 Sh*Ren* and 15 Sh*ATOH1* tumor-bearing mice fed a DOX diet underwent surgical resection of s.c. tumors and survived on the study thereafter for at least 22 days. Data are shown as percentage of animals displaying metastatic dissemination (disseminated tumor cells and micro/macro-metastases, red) or no metastatic dissemination in the liver (blue). Metastases were identified by human mitochondrion staining. (F) Representative images of human mitochondria, GFP and ATOH1 IHC staining in liver from Sh*Ren* DOX-fed and Sh*ATOH1#3* DOX-fed cohorts. Scale bars: 200 μm for human mitochondria and GFP; 100 μm for ATOH1. (G and H) Quantification of GFP (G) and ATOH1 (H) IHC staining in metastases from 2 DOX-untreated ShRen, 3 DOX-untreated ShATOH1#3, 4 ShRen DOX-fed, and 6 ShATOH1#3 DOX-fed mice. Data are geometric mean ± geometric SD. *p* values are reported as per two-tailed unpaired Mann Whitney U test. (I) *In vivo* study design to investigate development of metastasis following intracardiac implantation. Prior to cell implantation, ATOH1 depletion was DOX induced for 4 days *in vitro*, followed by sorting GFP+, viable cells by flow cytometry. Untreated control cells were sorted exclusively for viable cells. Animals in DOX-treated cohorts were fed a DOX-supplemented diet 24 h before implantation and kept on that diet until the endpoint. Animals in the uninduced control groups were given a standard diet. Animals from all 4 cohorts (ShRen with or without DOX and ShATOH1 with or without DOX) were removed at onset of symptoms (i.e., distended abdomen; detailed in STAR Methods) or after 70 days. (J) Kaplan-Meier curve of time to sacrifice. Control cohorts, fed a standard diet, are reported in scales of gray. *p* values were calculated with log rank Mantel-Cox test. (K) Quantification of metastatic liver dissemination for each cohort. Data are shown as in (D). (L) Quantification of metastatic liver cells per cohort. Metastatic cells were identified based on human mitochondrion staining. Data are shown as mean ± SD. *p* values were calculated with a two-tailed unpaired Mann Whitney U test. (M and N) Quantification of GFP (M) and ATOH1 (N) IHC staining in metastases from 5 DOX-untreated ShRen, 5 DOX-untreated ShATOH1, 5 ShRen DOX-fed mice, and 1 ShATOH1#3 DOX-fed mouse. Data are shown as geometric mean ± geometric SD. No statistical test could be performed, as ShATOH1 contained only one value.

We next asked whether the role of ATOH1 in maintaining cell survival *ex vivo* translated to impact on tumor growth *in vivo*. CDX17P control ShRen or ShATOH1(#3) cells were implanted subcutaneously (s.c.) in immunocompromised mice, and KD was induced with DOX-supplemented food after 19 days (Figure 6A), when mice had palpable tumors. Once tumors reached 500–800 mm^3^, they were surgically resected, and mice were kept on the study for 28 days to allow time for metastatic dissemination (based on previous experiments; STAR Methods; Figure 6A).

Significantly delayed s.c. tumor growth was observed in mice bearing DOX-induced ATOH1 KD tumors compared to DOX-induced ShRen controls or uninduced tumors (Figures 6B and 6C). This tumor growth delay extended time to experimental endpoint tumor volume or s.c. tumor surgical resection (22 days for ShRen, 35 days for ShATOH1, *p* < 0.0001; Figure 6D). To interpret the observed growth delay, we examined persistence of ATOH1 KD throughout the experiment by performing IHC for ATOH1 and GFP in resected s.c. tumors (mean tumor volume and time from implant: 603 ± 54 mm^3^, 44 ± 5 days ShRen +DOX; 552 ± 48 mm^3^, 70 ± 13 days ShATOH1 +DOX) (Figure 6B). At tumor resection, mice bearing DOX-induced ATOH1 KD tumors showed a 75% reduction in ATOH1 protein expression, and both DOX-induced controls and KD tumors had high expression of GFP (Figures S6A and S6B). However, GFP expression was ∼10% lower in DOX-induced ATOH1 KD tumors (Figure S6B, *p* = 0.008) and expression of GFP and ATOH1 was heterogeneous in DOX-induced ATOH1 KD tumors, with most tumor presenting with some GFP−, ATOH1+ regions (Figure S6C).

Overall, these data indicate that reduced ATOH1 expression promotes tumor growth delay *in vivo*, where impact may have been attenuated by outgrowth of ATOH1+ cells, which are potentially untransduced wild-type cells or cells that escaped inducible KD, as reported in other settings.^66^^,^^67^ These data are consistent with a selective pressure to re-instate ATOH1 expression in ATOH1 KD tumors, supporting a pro-tumorigenic role of ATOH1.

### A role of ATOH1 in liver-metastatic dissemination *in vivo*

We have reported previously that metastasis to multiple organs, including the brain and liver, occurs after resection of s.c. CDX17P tumors.^12^ To investigate whether ATOH1 supports metastatic growth, s.c. tumors were resected, and mice were left on the study for 28 days (Figure 6A) before metastasis (defined as >50 tumor cells) was quantified using a human mitochondrion antibody and IHC. Dissemination, predominantly to the liver, was observed in all cohorts regardless of DOX feeding, including single tumor cells and micro- or macro-metastases (Figure 6E). Although the frequency of liver metastases between control and DOX-induced ATOH1 KD mice was approximately equivalent, all liver metastases from DOX-induced ShATOH1 mice were negative for GFP and expressed similar levels of ATOH1 compared to uninduced tumors (Figures 6F–6H), again implying a selective pressure to retain/re-express ATOH1^66^^,^^67^ and indirectly suggesting a role of ATOH1 in promoting liver metastasis.

In a more direct approach to investigate the role of ATOH1 in metastasis, we performed intracardiac injection of tumor cells (Figure 6I), reasoning that liver metastasis would occur faster, allowing less time for outgrowth of cells with high or re-expressed ATOH1 (Figure 6F). CDX17P control ShRen or ShATOH1 cells were cultured with or without DOX for 4 days to induce ATOH1 KD *in vitro*, and GFP+ viable cells were sorted by flow cytometry before intracardiac injection. One group of mice per construct (ShRen and ShATOH1) received DOX-supplemented food (*n* = 5 ShRen and *n* = 8 ShATOH1), while control animals were maintained on a standard diet (*n* = 5 ShRen and *n* = 5 ShATOH1). Animals were removed from the study 70 days after intracardiac injection (STAR Methods; Figure 6I).

Almost all animals (14 of 15) in control cohorts (standard food or implanted with DOX-induced ShRen cells) were removed before the study endpoint due to extensive metastatic liver disease (Figure S6D). In contrast, 8 of 8 (100%) animals implanted with DOX-induced ShATOH1 cells reached the study endpoint (time from implantation: 53.6 ± 7.9 ShRen + DOX, 70 ± 0 ShATOH1 + DOX; Figure 6J). There was a significant reduction in metastatic burden in animals with ATOH1 KD compared to control cohorts (Figures 6K and 6L), and only one animal in the DOX-induced ShATOH1 group developed liver metastasis (Figure S6D). Despite showing positive GFP expression (>40% GFP+ cells), the only liver metastasis derived from ATOH1 KD cells also exhibited ATOH1 positivity in >60% of metastatic cells, indicating that ATOH1 KD was not completely retained in these cells (Figures 6M and 6N). Our data provide evidence that ATOH1 KD reduced metastasis to the liver and promoted longer survival. However, we cannot conclude that the pro-survival role of ATOH1 is the sole mechanism underpinning the aggressive metastasis to the liver or whether ATOH1 bestows additional pro-metastatic behaviors.

## Discussion

Emerging understanding of SCLC subtypes and phenotypic plasticity is considered key to support rational development of biomarker-directed personalized treatments.^14^ Building upon knowledge of inter- and intra-tumoral heterogeneity,^33^^,^^45^ we characterized the ATOH1 subtype, defining its prevalence and demonstrating pro-tumor functions of growth and metastasis.

ASCL1, NEUROD1, and ATOH1 are all pro-neural TFs negatively regulated by Notch signaling.^25^^,^^29^^,^^68^ While expression of ATOH1 is not reported during normal lung development, its expression has been reported in NE lung cancer,^69^ extrapulmonary high-grade NE cancers,^45^ MCC,^34^ medulloblastoma,^70^^,^^71^ and, rarely, in NSCLC^72^ and colorectal cancer (CRC).^31^^,^^73^^,^^74^ While mechanistically understudied, in medulloblastoma and MCC, ATOH1 is tumor promoting,^32^^,^^75^^,^^76^^,^^77^ whereas it is a tumor suppressor in CRC.^31^^,^^73^ These opposing context-dependent functions have been attributed to imbalance between differentiation and proliferation driven by abnormal ATOH1 expression levels.^78^

Co-expression of subtype TFs is commonly observed, contributing to SCLC heterogeneity.^12^^,^^33^^,^^79^^,^^80^ ATOH1 was found to be frequently expressed in SCLC clinical samples, either alone or with ASCL1 and/or NEUROD1 (Figures 1 and 2), extending existing sparse data.^69^ While in CDX models, ATOH1 was not co-expressed with ASCL1, and absolute expression levels of ATOH1 were generally lower in clinical samples than when present in CDXs, it was not possible to understand this heterogeneity in matched CDX tumors and their respective donor biopsy samples due to rarity of these donor samples. CDXs are generated from CTCs that were alive in the bloodstream when sampled and survived in the mouse. How this selection process affects the prevalence and distribution of subtype TFs is unknown. One could argue, however, that these CDX-generating CTCs are perhaps more likely to represent the patient-lethal clones than a small, single time point, often necrotic biopsy, where tissue sample bias is also a confounder.

In CDX30P, where ATOH1 was co-expressed with NEUROD1, while ATOH1 depletion did lead to increased NEUROD1 (Figure S5E), ATOH1 loss impacted cell survival *ex vivo* (Figure 5), suggesting that NEUROD1 did not compensate for ATOH1 loss and indicating that there is potential compensation between NEUROD1 and ATOH1 when both TFs are present. *NEUROD1* was not identified among ATOH1 direct targets, and there was minimal overlap with ASCL1 and NEUROD1 target genes (Figures S4F and S4G; Table S14), indicating that ATOH1 is not a NEUROD1 target in SCLC and in agreement with other data.^59^^,^^81^ As with NEUROD1 and ASCL1 in their respective subtypes,^82^^,^^83^^,^^84^^,^^85^^,^^86^^,^^87^ ATOH1 supports cell viability in ATOH1 subtype tumor cells (Figure 5).

In SCLC, ATOH1 exerts its function by binding E box motifs at promoter and distal regulatory elements of target genes as in the developing mouse brain^50^ and in MCC,^53^ including binding to its own downstream enhancer^23^ (Figure 3), although this does not definitively indicate that ATOH1 is present at active versus silent chromatin loci in SCLC. In CDXs, ATOH1 directly regulates expression of genes involved in neuronal fate development and mechanoreceptor differentiation (Figure 4), consistent with murine developmental studies and MCC.^22^^,^^34^^,^^88^^,^^89^ The ability of ATOH1 to regulate neuronal fate determination and Notch ligands (DLL1, DLL3, and DLL4) in mice^25^ mirrors the activity of ASCL1 in SCLC^59^^,^^82^; in CDX17P, ATOH1 depletion increased expression of non-NE and cell adhesion genes, invoking a similar role of ATOH1 in NE fate determination in SCLC (Figure S4). However, as the NE gene expression signature was retained upon ATOH1 depletion (Figure S4), additional factors; for example, MYC overexpression,^16^ are likely required to promote full NE-to-non-NE transition in ATOH1-driven SCLC. The need for additional signals to fully induce an NE-to-non-NE transition is similarly posited in studies of ASCL1 and NEUROD1 depletion in SCLC, where morphological changes or an NE-to-non-NE transition were not observed.^81^^,^^85^^,^^86^^,^^90^

Both *ATOH1* and *ASCL1* correlate with *MYCL* overexpression (Figure 1).^12^^,^^36^^,^^55^ In SCLC, overexpression/genetic amplification of *MYCL* was often correlated with the SCLC-A subtype, and *MYCL* is a direct transcriptional target of ASCL1.^16^^,^^59^ A more complex relationship was recently revealed by a clinical study where MYCL protein was present in only ∼30% of ASCL1+ samples.^80^ Adding to this heterogeneity, we show that all ATOH1-expressing CDXs present focal amplification and overexpression of MYCL (Figures 1 and S1). Correlation between ATOH1 and MYCL expression was also observed in MCC.^42^^,^^43^ However, we did not identify MYCL as a direct ATOH1 target (Table S12), and *MYCL* expression was unchanged upon ATOH1 depletion (Table S8; Figure 4). Combined, these data indicate that other factors contribute to *MYCL* expression in ATOH1+ SCLC.

The profound impact of metastasis on SCLC patient outcomes drives a pressing need to understand and target underlying mechanisms. Acquisition of neuronal gene expression programs is associated with invasive and metastatic SCLC in cell lines and genetically engineered mouse models (GEMMs).^66^^,^^91^^,^^92^ It was not possible to conclude whether the pro-survival phenotype of ATOH1 in CDX17P was the only cause of enhanced liver metastasis or whether ATOH1 promotes additional pro-metastatic behaviors. However, this study does draw parallels with the ATOH1 pro-invasive phenotype in MCC^76^ and its pro-metastatic role in medulloblastoma,^93^ where the ability of ATOH1 to suppress cell death was not explored. ATOH1 downregulation was linked with loss of cell adhesion (Figure 4B; Table S8), which was also observed in MCC.^34^^,^^94^

SCLC was once considered to derive from pulmonary NE cell precursors.^95^ However, elegant studies on SCLC GEMMs describe different potential cells of origin,^66^^,^^96^^,^^97^^,^^98^ with differences only evident at the molecular level.^16^^,^^46^^,^^59^ In this regard, similarities between MCC and ATOH1-driven SCLC are intriguing. MCC is an NE skin carcinoma, expressing epithelial and NE markers with morphological, ultrastructural, and immunohistochemical features shared with Merkel cells,^97^^,^^98^^,^^99^ yet there is no direct histo-genetic link between Merkel cells and MCC, with ongoing debate on cell(s) of origin of MCC.^99^^,^^100^ Tumor heterogeneity in MCC is attributed to variant disease etiologies mediated by either UV light exposure or MCPyV integration.^100^ Virus+ MCC has a low mutation burden, while virus-negative MCC, like SCLC, has characteristic RB1 and TP53 mutations in a highly mutated landscape.^101^^,^^102^ The recent identification of “mesenchymal-like” MCC with an inflamed phenotype exhibiting better response to immunotherapy draws parallels with the SCLC-I subtype^13^ and contrasts “immune-cold” immunotherapy-resistant MCC with higher expression of neuroepithelial markers, including ATOH1.^103^ That the ATOH1 subtype of SCLC CDX shares features with NE SCLC and with MCC, another NE cancer, is perhaps not surprising and might indicate convergent tumor evolution.^99^^,^^104^

In summary, we validate the ATOH1 SCLC subtype, where ATOH1 suppresses cell death and promotes tumor growth and metastasis. Further studies are needed to deepen our understanding of ATOH1-driven SCLC biology and to address whether there are therapeutic vulnerabilities of this subtype.

### Limitations of the study

The role of ATOH1 in cell survival and metastasis was explored using shRNA in our preclinical models generated from patient CTCs. We assume that CTCs that grow as CDXs represent an aggressive subpopulation within the tumor. Conditional KD had to be employed, as attempts to generate CRISPR knockouts of ATOH1 in CDX cells resulted in rapid cell death. While this highlights the critical role of ATOH1 in cell survival, this study is limited by incomplete KD of ATOH1 in 100% of SCLC cells, confounding interrogation of how ATOH1 contributes to SCLC metastasis via lengthy *in vivo* experiments. Intracardiac implantation (considered a “gold-standard” tool to study steps of the metastatic cascade after intravasation) was performed to shorten study time and consequently minimize loss of ATOH1 KD or re-expression. We combined this approach with positive selection of tumor cells with ATOH1 KD (GFP+ proxy) that remained viable. However, despite this, we could not determine whether the pro-survival role of ATOH1 was the sole contributor to metastatic liver colonization. Future experiments require profiling of proliferative states versus metastatic liver colonization at early time points. The ATOH1 subtype is rare, and the 4 CDX models described add to the single available established ATOH1 SCLC cell line.

## Resource availability

### Lead contact

Requests for further information, resources, and reagents should be directed to and will be fulfilled by lead contact, Caroline Dive (caroline.dive@cruk.man.ac.uk)

### Materials availability

This study generated an ATOH1 antibody (SY0287) (see key resources table); this was depleted during this study.

### Data and code availability

• Accession numbers for the raw RNA-seq and ChIP-seq data are in the key resources table.
• All new code generated is published in Zenodo (see Deposited Data section of the key resource table).
• Any additional information required to reanalyze data reported in this paper is available from the lead contact upon request.

## Acknowledgments

We acknowledge the following funders that enabled this research: the Cancer Research UK Manchester Institute (C5759/A27412), the Cancer Research UK National Biomarker Centre (CTRNBC-2022/100001), the 10.13039/501100020252Cancer Research UK Lung Cancer Centre of Excellence (BALCOE-Jun24/100005), the 10.13039/501100017008Cancer Research UK Manchester Centre (CTRQQR-2021\100010), the 10.13039/100013684Christie Charitable Fund, the Manchester National Institute for Health and Care Research (NIHR) 10.13039/100014653Manchester Biomedical Research Centre (NIHR203308), and the 10.13039/100000054National Cancer Institute (R35 CA263816 and U24 CA213274). Patient recruitment was supported by the 10.13039/501100000272National Institute for Health and Care Research (NIHR) 10.13039/100014653Manchester Biomedical Research Centre, the NIHR Manchester Clinical Research Facility at The Christie Hospital. Sample collection was undertaken through the CHEMORES protocol, the TARGET study, and the CONVERT protocol.

## Author contributions

K.L.S., C.D., and K.K.F. supervised and devised the study. A.C., M.P.-P., K.L.S., and C.D. co-wrote the manuscript. M.R., A.C., M.P.-P., D.M., and B.D.-W. performed immunohistochemistry analysis, data analysis, and interpretation. A.C. carried out experiments on CDXs and cell lines, ChIP-seq, RNA-seq, and western blotting, including data analysis and interpretation. K.S. and P.P.S. carried out *ex vivo* analyses. A.C., S.H., J.M.C., K.B., A.S.M.M.H., V.J.M., S.P.P., and A.K. carried out bioinformatics analyses. M.P.-P. designed and analyzed the *in vivo* metastasis studies. M.G., J.R., A.L., and D.C. carried out *in vivo* work. L.P., M.C., V.F., and F.B. oversaw the acquisition of ethical permission and patient consent and the collection of blood samples from patients in the CHEMORES and CONVERT studies. R.C. provided PDX along with C.M.R., who also assisted with manuscript revision. F.B. assisted with manuscript revision and is the chief investigator of the CHEMORES study. All authors read and approved the final manuscript.

## Declaration of interests

F.B. declares advisory board honoraria, speaker fees, and research funding from Amgen. C.D. declares research funding/educational research grants from AstraZeneca, Astex Pharmaceuticals, Biomodal, Bioven, Amgen, Carrick Therapeutics, Merck AG, Taiho Oncology, GSK, Bayer, Boehringer Ingelheim, Roche, BMS, Novartis, Celgene, Epigene Therapeutics Inc, Angle PLC, Menarini, Clearbridge Biomedics, Thermo Fisher Scientific, and Neomed Therapeutics; consultancy and/or advisory board honoraria from Biocartis, Merck, AstraZeneca, GRAIL, Boehringer Ingelheim, and VHIO; and personal remuneration from IFOM. C.M.R. has consulted for Amgen, AstraZeneca, Daiichi Sankyo, Hoffman-La Roche, and Jazz; serves on the scientific advisory boards of Auron, DISCO, and Earli and received royalty payments for DLL3-directed therapeutics licensing. J.M.C. consulted for Sonata Therapeutics.

## STAR★Methods

### Key resources table

**Table:** 

REAGENT or RESOURCE	SOURCE	IDENTIFIER
**Antibodies**		

SY0287 anti-ATOH1, rabbit polyclonal	In-house	SY0287 α-ATOH1
Anti-ATOH1, Rabbit polyclonal	Proteintech	Cat# 21215-1-AP; RRID: AB_10733126
Anti-Vinculin, mouse monoclonal	Sigma-Aldrich	Cat# V9264; RRID: AB_10603627
Anti-Lamin B, rabbit monoclonal	Abcam	Cat# ab229025; RRID: AB_3083735
Anti-NEUROD1, rabbit monoclonal	Abcam	Cat# ab213725; RRID: AB_2801303
Anti-MYC, rabbit monoclonal	Abcam	Cat# ab32072; RRID: AB_731658
Anti-SYP, rabbit monoclonal	Abcam	Cat# ab32127; RRID: AB_2286949
Anti-YAP1, rabbit monoclonal	Abcam	Cat# ab52771; RRID: AB_2219141
Anti-urokinase (PLAU), rabbit polyclonal	Abcam	Cat# ab24121; RRID: AB_447884
Anti-GFP D5.1, rabbit monoclonal	Cell Signaling Technology	Cat# 2956; RRID: AB_1196615
Anti-MASH1 (ASCL1), mouse monoclonal	BD Pharmingen	Cat# 556604; RRID: AB_396479
Anti-human mitochondria, mouse monoclonal	Abcam	Cat# ab92824; RRID: AB_10562769
Goat Anti-Rabbit Immunoglobulins/HRP	Agilent	Cat# P0448; RRID: AB_2617138
Goat Anti-Mouse Immunoglobulins/HRP	Agilent	Cat# P0447; RRID: AB_2617137
Anti-H3K4me3, rabbit polyclonal	Abcam	Cat# ab8580; RRID: AB_306649

**Bacterial and virus strains**		

T7 Express lysY/Iq Competent E. coli (High Efficiency)	NEB	Cat# C30131
NEB 5alpha competent E.coli (High Efficiency)	NEB	Cat# C2987U
One Shot™ Stbl3™ Chemically Competent E. coli	ThermoFisher	Cat# C737303

**Biological samples**		

Human Small Cell Lung Cancer Tissue (Biopsy, Tissue Microarray)	Manchester Cancer Research Center Biobank	https://mcrc.manchester.ac.uk
SCLC CDX Tissue	This Paper, (Simpson et al.^12^; Pearsall et al.^18^)	N/A

**Chemicals, peptides, and recombinant proteins**		

Human ATOH1 protein	This paper	N/A
2′-Deoxy-5-ethynyluridine (EdU)	Carbosynth	Cat# NE08701
Pierce™ SuperSignal™ West Pico PLUS Chemiluminescent Substrate	ThermoFisher	Cat# 34580
Puromycin	Sigma Aldrich	Cat# P8833
NucView® 405 Caspase-3 Enzyme	Biotium	Cay# 1040704077
Polybrene	Merck	Cat# TR1003G
ChIP cross-link Gold	Diagenode	Cat# C01019027
tetracycline-free FBS	Takara Bio	Cat# 631106
Doxycycline	Sigma Aldrich	Cat# D9891
Z-VAD-FMK	R&D Systems	Cat# FMK001
Q-VD-OPh	Insight Biotechnology	Cat# HY-12305
Necrosulfonamide	Tocris	Cat# 5025
Ferrostatin-1	Sigma Aldrich	Cat# SML0583
Propidium Iodide	ThermoFisher	Cat# P3566
Protease Inhibitor Cocktail	Sigma Aldrich	Cat# P8340
Cisplatin	Christie Pharmacy (Accord Pharmaceuticals)	In-house/Hospital Pharmacy
Etoposide	Sigma Aldrich	Cat# 33419-42-0
Matrigel	VWR	Cat# 734-0270
Doxycyline-supplemented diet	SSniff	Cat# A115D70541
Accutase	Sigma Aldrich	Cat# A6964
Insulin	Merck	Cat# I9278
Transferrin	Merck	Cat# T8158
hydrocortisone	Merck	Cat# H0888
sodium selenite	Merck	Cat# S5261
β-estradiol	Merck	Cat# E2758
Primocin	Invivogen	Cat# ant-pm-2
ROCK inhibitor	Tocris	Cat# 1254-10
DNAse I	Millipore Sigma	Cat# 11284932001
TryplE	Fisher Scientific	Cat# 10043382
FuGENE® HD	Promega	Cat# E2311
RNAlater®	Sigma Aldrich	Cat# #R0901
Phosphatase Inhibitor Cocktail II	Merck	Cat# P0044
Phosphatase Inhibitor Cocktail III	Merck	Cat# P5726

**Critical commercial assays**		

DNA extraction using Nucleobond® Xtra-midi EF kit	Macherey-Nagel	Cat# 740420.50
Live/Dead assay using LIVE/DEAD™ Fixable Far Red Dead Cell Stain Kit	Invitrogen	Cat# L34976
Live/Dead assay using LIVE/DEAD™ Fixable Violet Dead Cell Stain Kit (Invitrogen, L34963)	Invitrogen	Cat# L34963
QIAquick Gel Extraction Kit	Qiagen	Qiagen, 28706
RNeasy mini kit	Qiagen	Qiagen, 74104
QIAquick PCR Purification Kit	Qiagen	Qiagen, 28104
QIAprep Spin Miniprep Kit	Qiagen	Qiagen, 27106
Quick Ligation Kit	NEB	NEB, M2200
CellTiter-Glo® 3D luminescent assay	Promega	Cat# G9683

**Deposited data**		

Resource website for the sequencing data generated in this publication	This paper	“ENA: PRJEB85548”
Sequence data, analyses, and resources related to the RNA sequencing and ChIP sequencing of the CDX models in this paper	This paper	“Zenodo Data: https://doi.org/10.5281/zenodo.14866517”

**Experimental models: Cell lines**		

HCC33 human SCLC cell line	Leibniz Institute DSMZ-German Collection of Microorganisms and Cell Cultures	Cat# ACC487; RRID: CVCL_2058
H1339 human SCLC cell line	Creative Bioarray	Cat# CSC-C0516; RRID: CVCL_A472
HT29 human colon adenocarcinoma cell line	ATCC	Cat# HTB-38; RRID: CVCL_A8EZ
CDX cells	This paper (Simpson et al.^12^; Pearsall et al.^18^)	N/A
LentiX 293T cells	Clontech	Cat# 632180

**Experimental models: Organisms/strains**		

Mice: NOD SCID Gamma (NSG) (NOD.Cg-Prkdc^scid^Il2rg^tm1Wjl^/SzJ)	Charles River (refreshed and bred in-house)	Strain code 614

**Oligonucleotides**		

ATOH1 forward primer: TGGTGGACA GCAAATGGGTCGCGGATCCAT GAGCCGTCTGCTGCATG	This paper	AC_ATOH1_FRW-2
ATOH1 reverse primer: GGTGGTGCT CGAGTGCGGCCGCGCTT GCTTCATCACTATCGC TATAATG	This paper	AC_ATOH1_REV-2
Sequencing primer for PET28A and PET28A-ATOH1: GCTAGTTATTGCTCAGCGG	Addgene	T7-Terminal-REV
Sequencing primer for PET28A-ATOH1: TAATACGACTCACTATAGGG	Addgene	T7-FRW
Sequencing primer for PET28A: ACACCATCGAATGGCGCAA	This paper	LacI-FRW
Sequencing primer for mir-E constructs: TGTTTGAATGAGGCTTCAGTAC	Fellmann et al.^47^	mir-E CTRL primer
shATOH1#1: TGCTGTTGACAGTGAGCGAAGCGATGA TGGCGCAAAAGAATAGTGAAGCCACAG ATGTATTCTTTTGCGCCATCATCGCTGT GCCTACTGCCTCGGA	This paper	ATOH1 knockdown construct#1
shATOH1#3: TGCTGTTGACAGTGAGCGCCAACGAC AAGAAGCTGTCCAATAGTGAAGCCAC AGATGTATTGGACAGCTTCTTGTCGTT GTTGCCTACTGCCTCGGA	This paper	ATOH1 knockdown construct#3
shRenilla Luciferase(shRen), Ren.713	Fellman et al.^47^	N/A

**Recombinant DNA**		

LT3GEPIR vector for shRNA insertions	Fellmann et al.^47^	Addgene, Cat# 111177
PET-28A vector for ATOH1 insertion	Originally from Addgene, now in-house	N/A
pMDLg/pRRE for lentivirus production	a gift from Didier Trono (Dull et al.)^105^	Addgene, Cat# 12251
pCMV-VSV-G for lentivirus production	a gift from Bob Weinberg (Stewart et al.)^106^	Addgene, Cat# 8454
pRSV-Rev for lentivirus production	a gift from Didier Trono (Dull et al.)^105^	Addgene, Cat# 12253

**Software and algorithms**		

nf-core RNA-seq pipeline 3.2	Ewels et al.^107^	https://github.com/nf-core
STAR v2.6.1d	Dobin et al.^108^	https://github.com/alexdobin/STAR
bamcmp v2.0	Khandelwal et al.^109^	N/A
Limma v3.48	Ritchie et al.^110^	N/A
DESeq2 v1.32	Love et al.^23^	https://github.com/thelovelab/DESeq2
EnhancedVolcano v1.10	Blighe et al.^111^	https://githubcom/kevinblighe/EnhancedVolcano
pheatmap v1.0.12	Kolde et al.^112^	https://github.com/raivokolde/pheatmap
gProfiler2 v0.2.1	Kolberg et al.^56^	https://biit.cs.ut.ee/gprofiler/page/citing
Fast Gene Set Enrichment Analysis (FGSEA) v1.18	Korotkevich et al.^58^	https://github.com/ctlab/fgsea
R v4.1.0	https://www.R-project.org/	https://www.R-project.org/
ggplot2 v3.3.5	Wickham^113^	https://ggplot2.tidyverse.org/
nf-core ChIP-seq pipeline 1.2.1	Ewals et al.^107^	https://github.com/nf-core
Burrows-Wheeler Aligner (BWA) v0.7.17-r1188	Li et al.^114^	https://github.com/lh3/bwa
ChIPseq QC using MultiQC v1.9	Ewals et al.^115^	https://github.com/MultiQC/MultiQC
BEDTools v2.29.2	Quinlan and Hall^116^	https://github.com/arq5x/bedtools2
deepTools v3.4.3	Ramirez et al.^117^	https://github.com/deeptools/deepTools
ChIPSeeker v1.28.3	Yu et al.^118^	https://github.com/YuLab-SMU/ChIPseeker
ClusterProfiler v4.0.5	Yu et al.^119^	https://github.com/YuLab-SMU/clusterProfiler
profileplyr v1.8.1	Barrows and Carroll^120^	https://github.com/RockefellerUniversity/profileplyr
MEME ChIP	Machanick et al.^121^	https://meme-suite.org/meme
Binding and Expression Target Analysis (BETA, v.1.0.7)	Wang et al.^57^	https://github.com/suwangbio/BETA
IHC analysis: HALO™ Image Analysis Software	Akoya Biosciences	N/A
FCS Express v7	*DeNovo* Softwares	N/A

**Other**		

Publicly available RNAseq data from SCLC cell lines	The Broad Institute Cancer Cell Line Encyclopedia	https://portals.broadinstitute.org/ccle
Publicly available RNAseq data from surgically resected SCLC	George et al.^37^	https://github.com/dpeerlab/SCLC_atlas-HTAN
Publicly available RNAseq data from SCLC PDX	Caeser et al.^33^	E-MTAB-11230
Publicly available Transcriptome profile of Merkel Cell Carcinomas	Helsinki University	https://www.ncbi.nlm.nih.gov/bioproject/?term=775071

### Experimental model and subject details

#### Animal models for *in vivo* studies

All procedures were carried out in accordance with Home Office Regulations (UK), the UK Coordinating Committee on Cancer Research guidelines and by approved protocols (Home Office Project license 40–3306/70-8252/P3ED48266) and Cancer Research UK Manchester Institute Animal Welfare and Ethical Review Advisory Board). Six-weeks to ten-weeks old female NSG (NOD.Cg-Prkdc^scid^Il2rg^tm1Wjl^/SzJ) mice were housed and bred at Cancer Research UK Manchester Institute in specific pathogen-free conditions in individually ventilated cages (Home Office Project License no. PD673E295). All mice used were drug/test naive. A limitation of this study is that female mice had to be used, thus any potential differences due to mouse sex would not be captured within this study. Animals did not undergo previous procedures, were housed in a 12-h light/12-h dark environment, maintained at ambient temperature and humidity, and were given free access to food and water.

#### SCLC patient samples

65 patients described in this study had samples obtained between February 2012 and August 2016 following informed consent and according to ethically approved protocols. Sample collection was undertaken via the CHEMORES protocol (molecular mechanisms underlying chemotherapy resistance, therapeutic escape, efficacy and toxicity—improving knowledge of treatment resistance in patients with lung cancer), NHS Northwest 9 Research Ethical Committee ref. 07/H1014/96) and The TARGET (tumor characterization to guide experimental targeted therapy) study, approved by the North-West (Preston) National Research Ethics Service in February 2015, ref. 15/NW/0078. For 37 patients, sample collection was undertaken via the CONVERT protocol (concurrent once-daily versus twice-daily radiotherapy: a 2-arm randomised controlled trial of concurrent chemo-radiotherapy comparing twice-daily and once-daily radiotherapy schedules in patients with limited stage small cell lung cancer (SCLC) and good performance status.), the National Research Ethics Service, NHS Central Manchester research ethics committee, ref. 07/H1008/229. Patient metadata can be found in Table S4.

#### Cell lines and CDX *ex vivo* cultures

CDX models were generated from patients’ CTCs, as previously described.^38^ Once implanted in NSG mice, tumors were harvested at a maximum volume of 1200 mm^3^ and disaggregated *ex vivo* as described previously.^122^ Dissociated CDX cells were cultured in a humidity-controlled environment (37°C, 5% CO_2_) in HITES medium (RPMI 1640 supplemented with 50 μg/mL insulin (Merck, I9278), 100 μg/mL transferrin (Merck, T8158), 100 nM hydrocortisone (Merck, H0888), 300 nM sodium selenite (Merck, S5261-100), 100 nM β-estradiol (Merck, E2758)) supplemented with Primocin (Invivogen, ant-pm-2) and 5 μM ROCK inhibitor (Tocris, 1254-10). 2.5% fetal bovine serum (FBS, Labtech, FCS-SA) was supplemented after a week of *in vitro* culture to allow prior loss of any mouse fibroblasts.

The human SCLC cell line HCC33 (Leibniz Institute DSMZ-German Collection of Microorganisms and Cell Cultures, ACC 487) was cultured in RPMI 1640 supplemented with 20% FBS and 1% penicillin-streptomycin (Sigma-Aldrich, P0781). The human SCLC cell line H1339 was cultured in RPMI 1640 supplemented with 10% FBS and 1% penicillin-streptomycin. The human colon adenocarcinoma cell line HT29 (ATCC, HTB-38) was cultured in McCoy’s 5A medium (Sigma-Aldrich, P0781) supplemented with 10% FBS and 1% penicillin-streptomycin.

Most SCLC CDX *ex vivo* cultures and cell lines grow as suspension clusters. To obtain single cell suspensions, cells were incubated with DNAse I (Millipore Sigma, 11284932001) for 10 min at room temperature, following incubation with Accutase for 5 to 15 min at 37°C. H1339 and HT29 adherent cells were detached with TryplE (Fisher Scientific, 10043382) for 5 min at 37°C, after which TryplE was quenched with media. After detachment or dissociation to single cell suspension, cells were centrifuged at 300*g* for 5 min and cell density was determined by counting cell solutions diluted 1:1 with Trypan Blue (Sigma Aldrich, T8154) on a Countess 3 Automated Cell Counter (Invitrogen) as per manufacturer’s instructions.

Cell lines and CDX *ex vivo* cultures were routinely tested for mycoplasma by the MBCF within CRUK MI using a VenorGeM-qEP Mycoplasma Detection Kit (Cambio, 11–9250) run on a QuantStudio 5 Real-Time PCR System (Thermo Fisher Scientific). In addition, cell lines and CDX *ex vivo* cultures were authenticated by STR profiling using the Promega PowerPlex 21 kit (Promega, DC8902) and analyzed using genemapper5 software and an in-house database for comparisons/matching.

### Method details

#### *In vivo* studies

##### Cisplatin and etoposide treatment

CDX models were generated from patients’ CTCs, as previously described.^38^ To assess response to standard of care cisplatin and etoposide, cells from each CDX were implanted subcutaneously in 8–16 week-old female NSG mice in 100μL of a 1:1 mixture of RPMI (Gibco) and ice-cold Matrigel (VWR). 29 CDX were tested in *N* = 3 mice, with the following exclusions: CDX18, CDX23P, CDX42P, *N* = 4 mice; CDX8, CDX8P, CDX18P, *N* = 5 mice; CDX9, CDX12, *N* = 6 mice. CDX13 (SCLC-P subtype) and CDX29, CDX21 (SCLC-N subtype) were not tested. The total number of mice used in this study was *N* = 115, to test response to cisplatin/etoposide in *N* = 37 CDX models. Mice were randomized at 200–300 mm^3^ by assignment to vehicle or cisplatin and etoposide treatment groups, by deterministically distributing initial tumor volume sizes. Cohort size was guided by a study by Murphy et al.,^123^ demonstrating that a cohort size as few as one mouse can predict treatment response. 5 mg/kg cisplatin dosed at 5 mL/kg (Christie Pharmacy Ltd), 8 mg/kg etoposide dosed at 5 mL/kg (Sigma, 33419-42-0) in N-methyl-2-pyrrolidone (NMP) and citric acid, and vehicle compound (0.9% saline solution and NMP, respectively) was administered by intraperitoneal injection on day 1 and on days 1, 2, and 3, respectively, or corresponding vehicle control. Mice were monitored at least twice weekly by caliper measurements until tumors reached 1200 mm^3^ or until animal health deteriorated. Mice underwent treatment of up to 3 cycles of cisplatin/etoposide, 14 days apart; some animals did not tolerate the full treatment and/or tumors reached maximum allowed size before end of treatment and were therefore sacrificed after one cycle of chemotherapy. To account for these differences, we calculated pRECIST scores, adapted from ref.^39^ and ^40^) after 1 cycle of cisplatin/etoposide in all CDX models tested. pRECIST was calculated based on initial tumor volume (ITV) and relative tumor regression or tumor growth. Given the time at which the tumor volume first exceeds +300% growth (Tx), the tumor growth delay (TGD) was calculated as the ratio of Tx_Treated_/median(Tx_Vehicle_) to compare tumor growth in treatment arm to vehicle arm; whenever tumors failed to reach +300% growth due to ill health or tumor conditions, the Tx was estimated by linear regression of previous tumor measurements. Treatment response was then classed as: progressive disease 1 (PD1), if treatment arm displayed <50% regression from ITV during the study period and >25% increase in ITV with a TGD ≤1.5; PD2, if treatment arm displayed <50% regression from ITV during the study period and >25% increase in ITV with a TGD >1.5; stable disease (SD), if treatment arm displayed <50% regression from ITV during the study period and <25% increase in ITV at the end of dosing; partial response (PR), if treatment arm displayed >50% tumor regression and <90% for at least one time point compared to ITV; complete response (CR), if treatment arm displayed >90% tumor regression compared to ITV for at least one time point; maintained complete response (MCR), if treatment arm displayed a complete response for at least one doubling time. Doubling time was calculated on http://radclass.mudr.org/content/doubling-time-calculation-growth-rate-lesion-or-mass Website using first and last tumor volumes.

##### Induction of ATOH1 KD *in vivo*

CDX models were generated as previously described.^12^^,^^124^ To test the effects of ATOH1 depletion *in vivo,* ShRen andShATOH1#3 (referred to as ShATOH1 cohort) CDX17P cells were implanted subcutaneously in 8–16 week-old female NSG mice in 100μL of a 1:1 mixture of RPMI (Gibco) and ice-cold Matrigel (VWR). Before implantation, ShRen andShATOH1#3 CDX17P cells were treated with 1 μg/mL doxycycline (DOX) for 16 h and sorted by fluorescence-activated cell sorting (FACS) for positivity to eGFP. Then, cells were allowed to recover in culture and implanted in NSG mice 11 days after sorting. CDX17P is a very aggressive CDX and displays quick growth *in vivo* based on previous studies conducted in the lab, with an average time to 50 mm^3^ from implantation of 19 days (data not shown). Based on previous studies, mice were monitored daily for the first 3 weeks for signs of premature tumor growth. Because no premature growth was observed, mice were fed either standard (control cohort, 3 mice per construct) or doxycycline-supplemented diet (SSniff; A115D70541) 19 days post-implantation (experimental cohort, 15 mice per construct; total number of mice *N* = 36). Mice were then monitored 3 times weekly for body weight and tumor growth, with palpable tumors measured three times a week using calipers. Subcutaneous tumors were surgically removed once they reached a size between 500 and 800 mm^3^ and animals kept on study to allow the formation of metastases. Following resection of subcutaneous tumor, animals were sacrificed in accordance with the regulations outlined in Schedule 1 of the Animals (Scientific Procedures) Act 1986, either at 28 days post-resection or when a tumor regrew at site of resection and reached maximum size of 1200 mm^3^, whichever came first. The 28-day time point was selected based on previous studies on CDX17P showing liver metastasis occurring in 100% of the animals between days 23 and 39 following resection (on average 29.7 ± 7.4, data not shown). At the time of sacrifice, a full necropsy was performed, and organs were fixed in 10% formalin and embedded in paraffin for histopathological analysis.

Due to differences in tumor latency within and across cohorts, tumor growth data were aligned to the same starting volume of 50 ± 10 mm^3^ or first measurement if this was >60 mm^3^. Because tumor measurements were performed three times weekly and were aligned to the same starting tumor volume, the tumor growth data represented in Figure 6B were obtained by: 1. inferring missing measurements via linear regression across each 2 available measurements; 2. including the last and maximum tumor measurement, repeated for as many days as it took until the last animal on the cohort underwent surgical resection. The data was then represented graphically as mean ± standard deviation. The slope of the curves was calculated by transforming tumor growth data with a cubic distribution ($y=\sqrt[{3}]{x}$) and fitting a linear regression model; slopes were compared with ANCOVA in GraphPad Prism version 9.2.0. Kaplan-Meier curves were calculated as time from starting tumor volume (as defined above) to surgical resection of the subcutaneous tumor. Six mice in the ShRen +DOX cohort did not undergo surgical resection due to tumors exceeding 800 mm^3^ or due to tumor conditions (i.e., tumors attached to body wall); these mice were not censored in the analysis of the s.c. tumor growth and time to event was considered as time to maximum tumor volume of 800 mm^3^. Kaplan-Meier curves were compared with Log rank (Mantel-Cox) test. For the analysis of metastatic dissemination, only animals that underwent surgical resection of the s.c. tumor and survived at least 22 days were considered in the analysis (ShRen -DOX, *N* = 3; ShRen +DOX, *N* = 5; ShATOH1 -DOX, *N* = 3; ShATOH1 +DOX, *N* = 15).

##### Intracardiac implantation

To assess the role of ATOH1 depletion during metastatic dissemination*,* ShRen or ShATOH1#3 (referred to as ShATOH1 cohort) CDX17P cells were implanted into the left ventricle of the heart (intracardiac implants), in 8–16 week-old female NSG mice, resuspended in 50 μL of RPMI media. Prior to cell implantation, ATOH1 depletion was induced by treating cells with doxycycline (DOX) for 4 days *in vitro*, followed by sorting eGFP-positive viable cells by FACS. Untreated control cells were sorted exclusively for viable cells. Briefly, cells were treated with 1 μg/mL doxycycline (Sigma-Aldrich, D9891) for 4 days to induce eGFP expression and ATOH1 KD. Then, cells were dissociated to single cells with Accutase (Sigma-Aldrich, A6964) and washed once in PBS. Cells were then stained with LIVE/DEAD Fixable Violet Dead Cell Stain Kit (Invitrogen, L34963) diluted 1:1000 in PBS for 10 min at room temperature. Cells were washed in 2 mL of PBS and centrifuged at 300*g* for 5 min. LIVE/DEAD Fixable Violet Dead Cell Stain signal was measured upon excitation by violet laser (405 nm) using a 450/50 bandpass filter. GFP signal was measured upon excitation at 488 nm using a 530/30 bandpass filter. Positivity to each signal was based on unstained and single color controls. Single cell suspensions were filtered through a 50 μm filter and sorted on a BD Aria III flow cytometer (BD Biosciences). Each mouse was implanted with 250,000 cells and sufficient cells were obtained to implant 5 animals in the ShRen +/− DOX and ShATOH1 -DOX cohorts, and 8 animals in the ShATOH1 +DOX cohort. In addition, animals in the DOX treatment cohorts were given DOX-supplemented feed 24 h prior to implantation and they were kept on that diet until the end of the study, whereas animals in the untreated control groups were given a standard diet. Animals from all 4 cohorts (ShREN +/−DOX and ShATOH1 +/−DOX) were removed at the onset of symptoms of liver metastatic disease (enlarged abdomen, firm at palpation) or after 70 days. The 70-day endpoint was based on a previous experiment showing liver metastasis occurring in 100% animals between days 56–91 following intracardiac injection (on average 71.4 ± 15.2). Animals were sacrificed following regulations outlined in Schedule 1 of the Animals (Scientific Procedures) Act 1986, and full necropsies were performed. All livers were kept as formalin-fixed, paraffin-embedded tissue for immunohistochemistry.

Cell implants and animals procedures were carried out in the morning on a laminar air flow bench and mice placed back in their home cages; tumor resections and intracardiac implantations were carried out under a class II laminar flow cabinet in aseptic conditions. The study was designed to detect a 1.5 effect size, with 70% power and alpha = 0.05 based on previous studies of CDX17P. Normality of data from previous studies on CDX17P was confirmed performing Shapiro-Wilk test and the power calculations performed with a t test using R version 4.1.0. In this experiment, blinding was not implemented, and confounders, as classified by the ARRIVE guidelines, were not controlled for.

#### Generation of stable genetically modified cells

##### Plasmid generation

To generate stable ATOH1 knockdown cell lines and CDX, mir-E-shRNAs targeting ATOH1 were designed using splashRNA^125^ and control sequences targeting Renilla Luciferase (Ren.713) were derived from Fellman et al.^47^ and inserted into the LT3GEPIR vector (Addgene, 111177) according to previously published protocols.^47^ Plasmid DNA Sanger Sequencing was used to verify successful ligations and was performed by the Molecular Biology Core Facility (MBCF) within Cancer Research UK Manchester Institute (CRUK MI) using an ABI3130xl 16 capillary system. Sanger Sequencing results were visualised with Chromas v2.6.4 (Technelysium Pty Ltd). Correctly ligated plasmids were prepared for cell transfection by recovery of bacterial cultures and isolation of DNA using Nucleobond Xtra-midi EF kit (Macherey-Nagel, 740420.50), according to manufacturer’s instructions.

##### LentiX 293T cell transfection

LentiX 293T cells (Clontech) were cultured in high glucose, pyruvate DMEM (Thermo Fisher, 41966052) supplemented with 10% FBS. LentiX 293T cells were transfected at 70% confluency in a 6 well plate with 880 ng of Human ATOH1 ORF clone in Mammalian Expression Vector (GenScript, OHu29710), using 6 μL of FuGENE HD (Promega, E2311) in 100 μL of Opti-MEM Reduced Serum Media (ThermoFisher, 31985062). After 72 h, protein lysates were harvested for further analysis by immunoblot.

##### Lentiviral production in LentiX 293T cells

To produce lentiviruses, LentiX 293T cells were transfected at 70% confluency in a 10 cm dish with 8.5 μg transfer plasmid, 3.4 μg pMDLg/pRRE (a gift from Didier Trono, Addgene #12251),^105^ 1.7 μg pCMV-VSV-G (a gift from Bob Weinberg, Addgene #8454),^106^ and 3.4 μg pRSV-Rev (a gift from Didier Trono, Addgene #12253)^105^ using FuGENE HD (Promega, E2311). The medium was refreshed after 24 h and virus was harvested 48- and 72-h post transfection and filtered through a 0.45 μm acrodisc syringe filter (VWR, 514–4101). Viral supernatant was concentrated with PEG-IT (autoclaved 5X solution of 100 g of PEG, 6 g NaCl and 250 mL ddH2O, pH 7.2). PEG-IT was added to the viral supernatant at 1:5 ratio and incubated for at least 12 h at 4°C; the mixture was then centrifuged at 1,500*g* for 30 min at 4°C and resuspended in serum-free HITES medium at the desired concentration.

##### Cell transduction and selection

Stable cell lines were obtained by transducing a single cell suspension of 5,000,000–10,000,000 CDX cells, either previously kept in culture or obtained directly from tumor disaggregation, with virus to a 2X final concentration in Serum free HITES medium, supplemented with 12 μg/mL polybrene (Merck, TR-1003-G). After 24 h, virus containing medium was replaced with fresh HITES supplemented with tetracycline-free FBS (Takara Bio, 631106). Cells obtained directly from disaggregation were cultured after transduction for a week without FBS to avoid outgrowth of mouse fibroblasts. 48 h post-transduction, puromycin (Merck, P8833) was added to the media at 1 μg/mL to select cells correctly transduced. After a week of selection with puromycin, cells were assessed for mycoplasma and injected subcutaneously in NSG mice in a 1:1 mixture with Matrigel (BD Biosciences, 354234) to amplify selected cells. When at size, tumors were disaggregated as previously described^122^ and cells were kept under selective pressure with 1 μg/mL puromycin at all times.

To ensure purity of the cell population when transduction rates were low, cells were sorted on a BD Aria III flow cytometer (BD Biosciences). Cells were treated with 1 μg/mL doxycycline (Sigma-Aldrich, D9891) O/N to induce GFP expression. Cells were dissociated to single cell suspension and stained with LIVE/DEAD Fixable Far Red Dead Cell Stain Kit (exc: 633 or 635 nm) (Invitrogen, L34976) diluted 1:1000 in PBS for 10 min at room temperature. Cells were washed in PBS and filtered with Flowmi Cell strainers (Fisher Scientific, 15342931) just before acquisition. GFP-positive, live cells were collected in cold PBS and immediately put back in culture with warm media.

#### *Ex vivo* drug treatments

##### Treatment of SCLC cell lines with cisplatin and etoposide

The sensitivity of ASCL1 and NEUROD1 SCLC cell lines to cisplatin and etoposide monotherapy was evaluated using the CellTiter-Glo 3D luminescent assay (Promega, G9683). Cells were seeded at a density of 2,000 cells per well in 384-well microplates (Greiner, 781080) with the use of the Integra Viaflo Assist platform (Integra Biosciences). Cells were incubated for 24h prior to the addition of the compounds.

Cisplatin (Christie NHS Foundation trust) and etoposide (Sigma-Aldrich, E1383) were tested across nine concentrations (0.03 μM–30 μM) using a 3-fold dilution series, with corresponding vehicle controls (PBS and DMSO, respectively). The compounds were added with the automated drug dispenser Echo 550 liquid handler (Labcyte Inc.). The CellTiter-Glo 3D luminescent assay was used at the time of compound addition (T0) and at the experiment’s endpoint (day 5) to assess cell doubling efficacy and compound potency, respectively. Concentration-response curves were generated with a four-parameter log-logistic model (4pLL) using GraphPad Prism v10.0.2 (Boston, Massachusetts, USA).

##### Induction of ATOH1 KD with doxycycline

Cell viability assays to assess effects of ATOH1 depletion were performed after 14 days treatment with doxycycline. Cells were seeded at 100,000 cells/mL in T75 flasks and doxycycline added fresh to the media every 2–3 days; after 7 days, cell clusters were dissociated to single cell suspension and 10,000 suspension cells were seeded in triplicate in 200 μL of media for 7 days with doxycycline additions every 2–3 days. At this point, doxycycline was either supplemented again or withdrawn to restore ATOH1 expression.

Cell viability upon ATOH1 KD, was quantified with 20 μL CellTiter-Glo 3D luminescent assay (Promega, G9683) per well, incubated for 30 min to stabilise the signal, before reading luminescence on a FLOUStar Omega plate reader (BMG LABTECH). One plate was processed on the same day of seeding, for normalization purposes, and one after 7 days treatment. Changes in viability were calculated as fold change in luminescence signal between the day of seeding and day 7; then, doxycycline-untreated controls per each cell line (ShRenilla, ShATOH1#1, ShATOH1#3) served to calculate fold changes relative to doxycycline-treated or withdrawn conditions. Cell death and apoptosis was assessed by flow cytometry (described below).

#### Flow cytometry assays

##### Cell death and apoptosis assay

ATOH1 KD stable CDX lines and HCC33 were seeded as described above to induce ATOH1 KD and assayed for detection of apoptosis and cell cycle progression (see below) after a total of 14 days in DOX. Similarly, CDX *ex vivo* cultures were seeded at seeded at 100,000 cells/mL in T25 flasks and treated after 24 h with 0.25, 0.5 and 1 μM CCS1477 for 7 days. After the time of treatment, cells were dissociated to single cells with Accutase or detached to single cells with TryplE as described above, and washed once in PBS. Cells were then stained with LIVE/DEAD Fixable Far Red Dead Cell Stain Kit (Invitrogen, L34976) diluted 1:1000 in PBS for 10 min at room temperature. Cells were washed in 2 mL of PBS and centrifuged at 300*g* for 5 min. To detect apoptosis, cells were stained with 200 μL of 2 μM NucView 405 Caspase-3 Enzyme (Biotium, 10407) solution in PBS for 20 min at room temperature. NucView 405 signal was measured upon excitation by blue laser using 450/50 bandpass filter; LIVE/DEAD Fixable Far Red Dead Cell Stain signal was measured upon excitation with red laser using 780/60 bandpass filter. Positivity to each signal was based on unstained and single color controls.

##### Cell cycle analysis

For cell cycle progression analysis, CDX17P and HCC33 cells carrying inducible control ShRen or ATOH1 KD were treated with DOX or vehicle for 14 days, as described above; cells were incubated with 2 μM 2′-Deoxy-5-ethynyluridine (EdU) (Carbosynth, NE08701) for 2 h at 37°C. Cells were harvested, dissociated to a single cell suspension and fixed with 100 μL of 4% formaldehyde (Sigma Aldrich, F8775) diluted in PBS for 15 min at room temperature. Cells were washed with PBS containing 2 mM EDTA (Thermo Fisher, 15575020) and centrifuged at 300*g* for 5 min. Pellets were permeabilized with PBS containing 0.5% Triton X-100 solution for 15 min at room temperature. The EdU Click reaction cocktail (4 mM CuSO_4_ (Acros, 197730010), 5 μM Sulfo-Cyanine 5 Azide (Lumiprobe, B3330), 100 mM Sodium ascorbate (Acros, 352685000)) was then prepared fresh and added to the cells for 30 min at room temperature. Cells were washed with PBS containing 2 mM EDTA, centrifuged and stained with 3 μM DAPI (Fisher Scientific, 10184322) diluted in PBS containing 2 mM EDTA for 15 min at room temperature. EdU signal was measured upon excitation by red laser using 660/20 bandpass filter; DAPI signal was measured upon excitation by blue laser using 450/50 bandpass filter. Positivity to each signal was based on unstained and single color controls.

Due to variability of EdU incorporation rates, cell cycle progression in CDX30P was assayed using ethanol fixation followed by propidium iodide (PI) staining. Cells were treated with doxycycline as above; after 14 days of DOX treatment, cells were dissociated to single cell suspension and stained with LIVE/DEAD Fixable Violet Dead Cell Stain Kit (Invitrogen, L34976) diluted 1:1000 in PBS for 10 min at room temperature. Cells were washed and fixed in 1 mL 70% Ethanol added dropwise. Cells were fixed for at least O/N at −20°C. On the day of assay, cells were washed in PBS and stained with 40 μg/mL PI and 60 μg/mL RNase A diluted in PBS. PI signal was measured upon excitation by yellow laser using 586/20 bandpass filter; LIVE/DEAD Fixable Violet Dead Cell Stain signal was measured upon excitation by blue laser using 450/50 bandpass filter. Positivity to each signal was based on unstained and single color controls.

Samples were filtered with Flowmi Cell strainers (Fisher Scientific, 15342931) before acquisition, with flow cytometry data obtained using NovoCyte (Agilent), BD FACSCanto II (BD Bioscience) or BD LSRFortessa (BD Bioscience).

#### ATOH1 antibody production

The full human protein sequence of ATOH1 was codon optimized for expression in bacteria (ThermoFisher GeneArt) and corresponding DNA sequences were ordered from ThermoFisher Scientific. 0.05 ng of template DNA was PCR-amplified with primers carrying PET-28A homology arms (see DNA sequences and primers) with Q5 High-Fidelity DNA Polymerase (NEB, M0491S) according to manufacturer’s instructions and with the following protocol: 98°C for 1 min; 30 cycles at 98°C for 10 s, annealing at 66°C for 30 s and extension at 72°C for 40 s; final extension at 72°C for 2 min. PCR buffers were removed using a QIAquick PCR Purification Kit (Qiagen, 28104) and both recipient plasmid PET-28A and ATOH1 DNA sequence underwent restriction digest with BamHI-HF and NotI-HF (NEB, R3136 and R3189) for 2 h at 37°C, followed by 20 min at 65°C to inactivate the restriction enzymes. Digested PET-28A was de-phosphorylated with 2U/μg Alkalyne Phosphatase (Sigma-Aldrich, 10713023001) for 1 h at 37°C, run on 1% agarose gel and purified with a QIAquick Gel Extraction Kit (Qiagen, 28706). ATOH1 DNA sequences were purified with a QIAquick PCR Purification Kit after restriction digestion. Ligation was performed with a Quick Ligation Kit (NEB, M2200) using 50 ng of recipient PET-28A vector and 25 ng of ATOH1 DNA fragment, representing 3:1 M ratios; 4μL of ligation reactions were transformed in NEB 5alpha competent E.coli (High Efficiency) (NEB, C2987U) according to manufacturer’s instructions. Briefly, cells were incubated with the plasmid on ice for 30 min and heat shocked at 42°C for 35 s; cells were subsequently allowed to recover in 900 μL Super Optimal broth with Catabolite repression (SOC) media at 37°C for 1 h, plated on 10 cm diameter agar plates containing Lysogeny Broth (LB) and 50 μg/mL kanamycin A (Sigma-Aldrich, K4000-25G) and allowed to grow O/N. Plasmids were isolated using a QIAprep Spin Miniprep Kit (Qiagen, 27106) and successful cloning was verified by Sanger sequencing performed by the MBCF of the CRUK MI.

To produce recombinant, human ATOH1 protein, 10 to 50 ng PET28A-ATOH1 were transformed in T7 Express lysY/Iq Competent E. coli (High Efficiency) (NEB, C3013I) according to manufacturer’s instructions. The next day, colonies were picked and cultured in 5–10 mL LB with Kanamycin An O/N. In the morning, the culture density was checked with a spectrophotometer and the culture was diluted to 0.01 OD in 1L of LB with Kanamycin A. OD was checked regularly and induction performed with 1 mM IPTG (Promega, V3955) when OD reached 0.4 for 3 h. After 3 h, bacterial cultures were pelleted at 3,500 rpm for 15 min and resuspended in 20 mL buffer A (50 mM Tris pH 8.0 (Sigma-Aldrich, T3253-500G), 100 mM NaCl (Sigma-Aldrich, S3014-500G), 15 mM MgSO_4_ (SLS, M7506-500G), 0.1 mM Dithiothreitol (DTT, Sigma-Aldrich, 10197777001), 0.2 mM phenylmethylsulfonyl fluoride (PMSF, Sigma-Aldrich, P7626-250MG)) and snap frozen. Pellets were lysed by defrosting at 37°C for 1 h with shaking and then by adding 4 mg of lysozyme (Sigma-Aldrich, L6876-1G) for 1 h at 37°C. After 2 h, DNAse I (Sigma-Aldrich, DN25-100MG) was added at final concentration 100 μg/mL and incubated for 30 min at 37°C. Lysed cells were centrifuged for 15 min at 10,000 rpm, the supernatant discarded and the pellet resuspended in 20 mL buffer A with 0.5% sodium deoxycholate (Sigma-Aldrich, D6750-100G), 1% Triton X-100 (Sigma-Aldrich, T8787-100ML) and incubated on ice for 15 min. Samples were centrifuged at 10,000 rpm for 15 min, following which the supernatant containing the soluble fraction was discarded and the pellet containing the inclusion bodies was washed once in buffer A. The remaining pellet was resuspended in 3 mL PBS with 1 mM PMSF and 1.5 mL 3X SDS Blue Loading Buffer (NEB, B7703S), incubated at 99°C for 5 min and run on 12% polyacrylamide gel on PROTEAN II xi Cell O/N at 50 mA at 4°C. After 12–16 h the gel was stained with Comassie staining solution (0.25% comassie brilliant blue R-250 (Bio-Rad, 161–0400), 50% methanol and 10% acetic acid) and the relevant band was cut out of the gel. The protein was recovered from the polyacrylamide gel by electroelution. Gel bands were wrapped in dialysis tubes (SLS, D6191-25EA) and eluted overnight at 100 mA in elution buffer (29.01 g/L Na_2_HPO_4_·12H_2_O (Sigma-Aldrich, 71640), 2.96 g/L NaH_2_PO_4_·2H_2_O (Sigma-Aldrich, S3139), 0.288 g SDS (Sigma-Aldrich, L3771), pH 7.4) in an electrophoresis tank. Eluted protein was collected after O/N electroelution, concentrated to ∼10 mL in Vivaspin Protein Concentrator Spin Columns (GE Healthcare, 28-9323-60) and dialyzed in Slide-A-Lyzer Dialysis Cassettes (Fisher Scientific, 66810) in electroelution buffer without SDS, O/N at 4°C. Protein concentration was determined by running 12% SDS-PAGE gels with a standard curve of BSA, staining with Comassie blue solution and calculating band intensity of standard curve with Fiji^126^ and interpolating the band intensity of dilutions of target purified proteins. 1 mg of ATOH1 was sent to Eurogentech for immunization of one rabbit (SY0287) with the recombinant protein using their 28-day protocol. Serum collected on the last day of the protocol was tested by immunoblot to assess general specificity and then affinity purified to target ATOH1 with AminoLink (Thermo Fisher, 44894). Columns were equilibrated with two washes with pH 10 coupling buffer, and coupled to 5 mg of recombinant protein, diluted 1:3 in pH 10 coupling buffer, O/N on an end-over-end rotator. Columns were washed twice in pH 7.2 coupling buffer and coupling reaction was performed in 2 mL pH 7.2 buffer supplemented with 40 μL 5M Sodium Cyanoborohydride Solution O/N at 4°C. The reaction was quenched with 2 mL quenching buffer twice and remaining active sites blocked with 2 mL quenching buffer supplemented with 40 μL 5M Sodium Cyanoborohydride Solution at room temperature for 30 min. The columns were then washed with washing buffer at least 5 times, equilibrated with storage buffer and stored at 4°C until use. To purify reactive antibodies, columns were equilibrated to room temperature and washed with 6 mL Wash Solution and 4 mL of serum were incubated with the resin for 1 h at room temperature on a rotator. The column was washed 7 times with 2 mL Wash Solution and antibodies eluted with 10 mL Thermo Scientific Pierce Binding and Elution Buffers for IgG (Fisher Scientific, 21004) and collected in 1 mL fractions, neutralized with 50 μL 1M Tris HCl at pH 9.0. 10 μL of each fraction was run on a 12% gel to determine which fractions contain the highest amount of IgG; in this case, fractions 3, 4 and 5 were the most enriched for IgG and were dialyzed in PBS O/N at 4°C in a Slide-A-Lyzer Dialysis Cassette (Fisher Scientific, 66380). After dialysis, the antibody was concentrated with Vivaspin Protein Concentrator Spin Columns and concentration determined with a Pierce BCA Assay kit (Thermo Fisher, 23225); the antibody was diluted to a concentration of 0.7 mg/mL in 1 volume of glycerol supplemented with 0.005 Sodium Azide (Sigma-Aldrich, S2002).

#### Transcriptomics

##### SCLC CDX biobank

RNA was obtained from three independent RNAlater (Sigma-Aldrich, #R0901) treated tumors from each CDX model as previously described.^12^^,^^18^^,^^124^ No new CDX were characterised in this study. Downstream analysis was performed as detailed below. NE score was calculated based on the newest NE and Non-NE signatures,^60^ as previously described.

##### Detection of MCPyV

Detection of MCPyV transcript was performed by aligning raw RNA-Seq reads using STAR v2.7.9a^108^ to MCPyV reference genome (NC_010277.2). The results were validated by using virus positive and negative human MCC samples from a publicly available dataset (BioProject 775071) and results were reported as uniquely mapped reads.

##### CDX17P with ATOH1 depletion

CDX17P ShRenilla (ShRen), ShATOH1#1 and ShATOH1#3 cells were seeded at 100,000 cells/mL for 7 days and treated with 1 μg/mL doxycycline where appropriate. After 6 days of DOX treatment, with fresh DOX addition every other day, cells were harvested and RNA extracted with RNeasy mini kit (Qiagen, 74104). RNA was processed similarly to the CDX biobank already described.^12^^,^^18^ Briefly, RNA was quantified using a Qubit RNA HS Assay kit (Thermo Fisher Scientific, Q32855) and RNA with an integrity number >8 determined using a Bioanalyzer RNA 6000 Nano assay (Agilent, 5067-1511) was taken forward to generate libraries. Indexed PolyA libraries were prepared using 200 ng of total RNA and 14 cycles of amplification with the SureSelect Strand Specific RNA-seq Library Preparation kit for Illumina Sequencing (Agilent, G9691B). Library quality was assessed using the Agilent Bioanalyzer. Libraries were quantified by qPCR using the Kapa Library Quantification Kit for Illumina (Roche, 07960336001). Paired-end 2 × 75 bp sequencing was undertaken on a NextSeq 500 sequencer (Illumina Inc.).

#### ChIP-seq

ChIP-Seq was performed on CDX17P ShRenilla (ShRen) and ShATOH1#3, after 6 days treatment with 1 μg/mL DOX, according to published protocols.^127^ Cells were dissociated to single cell suspension and counted, as described above. For ATOH1 ChIP-Seq, 50 million cells underwent a dual crosslink, where cells were incubated for 30 min in 50 mL PBS with 1 mM MgCl_2_ and 200 μL ChIP Crosslink Gold (Diagenode, C01019027), washed three times in PBS and crosslinked in 50 mL 1% formaldehyde solution in PBS for 20 min (Sigma-Aldrich, F8775). For H3K4me3 ChIP-Seq, 10 million cells were crosslinked in 10 mL 1% formaldehyde for 20 min. Crosslinking was quenched with glycine (Sigma-Aldrich, G8898) to 0.125 M final concentration. Cells were washed three times in cold PBS and pellets snap frozen at −80°C. Upon thawing, cell pellets were lysed in 10,000,000 cells/mL lysis buffer 1 (LB1, 50 mM HEPES (Sigma-Aldrich, H4034), 140 mM NaCl, 1 mM EDTA (Thermo Fisher, 15575020), 10% glycerol (Sigma-Aldrich, G9012), 0.75% NP-40 (Thermo Fisher, 85124), 0.25% Triton X-100) supplemented with protease inhibitor cocktail (PIC, Sigma-Aldrich, P8340) for 10 min at 4°C on a rotator. Pellets were centrifuged at 2200*g* for 5 min to collect cells and lysed in 10,000,000 cells/mL LB2 (200 mM NaCl, 1 mM EDTA, 0.5 mM EGTA (Generon, 40121266-2), 10 mM Tris-HCl pH 8) supplemented with PIC for 10 min at 4°C on a rotator. Pellets were centrifuged at 1800 rpm for 5 min and resuspended in sonication buffer LB3 (10 mM Tris-HCl pH 8, 100 mM NaCl, 1 mM EDTA, 0.5 mM EGTA, 0.1% Na-Deoxycholate (Sigma-Aldrich, D6750), 0.5% N-lauroylsarcosine (Sigma-Aldrich, L9150), 300 μL per 10,000,000 cells and moved to 1.5 mL sonication tubes (Diagenode, C30010016). Lysates were sonicated at 30 s on/30 s off for 8 cycles (H146) and 18 cycles (CDX17P) in a Bioruptor Pico (Diagenode) kept at 4°C. Optimal DNA shearing (200–400 bp) was assessed on 10 μL of sample by clearing the lysate with 1 μL 10% Triton X-100, centrifugation at maximum speed for 2 min and by reversing crosslinks with 1 μL Proteinase K (Thermo Fisher, EO0491) for 15 min at 65°C. Lysates were cleared again by centrifugation and supernatant (10 μL) was supplemented with 2 μL 6X loading dye (NEB, B7024S) and run on a 1% agarose gel at 120V for 20 min.

Antibody-coupled magnetic beads were prepared 24–72 h in advance. 10 μL/1 μg antibody Diamag protein G-coated magnetic beads (Diagenode, C03010021-150) were washed twice in 1.5 mL cold PBS 0.5% BSA (Sigma-Aldrich, A3608) on a magnetic rack and incubated at least O/N with 5 μg ATOH1 (in-house SY0287; ProteinTech, 21215-1-AP) or 1.5 μg H3K4me3 (abcam, ab8580) per sample. Before use, surplus antibody was removed, and beads washed three times with 1.5 mL cold PBS 0.5% BSA. Sonicated DNA was incubated with antibody-conjugated beads in LB3 O/N at 4°C on an end-over-end rotator.

After O/N incubation, the DNA and beads mixture was washed 5 times in RIPA buffer (50 mM HEPES-KOH pH 7.55, 500 mM LiCl (SLS, L9650), 1 mM EDTA, 1% NP-40, 0.7% Na-Deoxycholate) and once with TE buffer (10 mM 50 mM Tris-HCl pH 8, 1 mM EDTA) supplemented with 50 mM NaCl. DNA was eluted by incubating the mixture at 65°C for 15 min with 110 μL elution buffer (50 mM Tris-HCl pH 8, 10% SDS, 1 mM EDTA); 100 μL of the supernatant was removed, and a second elution was performed with 100 μL elution buffer for 10 min at 65°C. Crosslink reversal was performed O/N (maximum 18 h) at 65°C. To remove RNA, samples were diluted with 200 μL TE buffer, supplemented with 8 μL RNAse A (Thermo Fisher, EN0531) and incubated for 2 h at 37°C. Finally, 4 μL proteinase K was added to each sample and samples were incubated at 55°C for 2 h. Phenol:chloroform extraction was used to purify ChIP-ed DNA: 400 μL phenol:chloroform:isoamyl alcohol (Fisher Scientific, 11518756) was added to each sample and samples moved to MaXtract High Density phase-lock tubes (Qiagen, 129056); tubes were centrifuged for 3 min at 16,000*g* and aqueous phase was moved to a new tube with 16 μL 5M NaCl, 30 μg glycogen (Sigma-Aldrich, 10901393001) and 800 μL 99% EtOH and incubated O/N at −80°C. DNA was pelleted by centrifugating tubes at 20,000*g* for 10 min at 4°C and washed with 80% EtOH. Pellets were air-dried for 15 min and resuspended in 70 μL of 10 mM Tris-HCl, pH 8.0. Afterward, samples were quantified at Qubit (Thermo Fisher); 1 ng of DNA was used for library preparation with NEBNext Ultra II (NEB, E7645) according to manufacturer’s instructions and samples were sequenced on a Novaseq 6000 (2x100 cycles) (Illumina Inc.).

#### Western blotting and nuclear fractionation

Whole cell lysates were obtained from cell pellets or flash-frozen CDX tumor tissue by incubating with CST cell lysis buffer (Cell Signaling Technology, 9803S) supplemented with Protease Inhibitor Cocktail (PIC, Merck, P8340) and Phosphatase Inhibitor Cocktail II (Merck, P0044) and III (Merck, P5726) for 15 min on ice. Flash-frozen CDX tumors were homogenized in Fastprep tubes with matrix A using TissueLyser LT, at 50 Hz for 3 × 60 s, in ice-cold lysis buffer. Tubes were centrifuged at ≥ 16,000*g* for 1 min at 4°C.

Nuclear lysates were obtained from 5 to 10 million *ex vivo* cultured cells. The cytoplasmic fraction was isolated with 500 μL of hypotonic buffer (20 mM Tris-HCl pH 7.4, 10 mM NaCl, 3 mM MgCl_2_) supplemented with PIC and phosphatase inhibitors for 15 min on ice; the mixture was supplemented with 25 μL 10% NP-40 (Sigma) and vortexed, before centrifuging at 3000 rpm for 10 min. Remaining nuclear pellets were washed in 1 mL hypotonic buffer supplemented with 25 μL 10% NP-40 and centrifuged at 3000 rpm for 10 min. Nuclei were lysed in 50 μL CST cell lysis buffer supplemented with PIC and phosphatase inhibitors for 30 min. During this time, samples were sonicated in Bioruptor Pico (Diagenode) for 3 to 5 cycles, 10 s ON, 30 s OFF. Nuclear lysates were cleared by centrifugating at 14,000*g* for 30 min. All centrifugation steps and incubations were performed at 4°C.

Protein lysates were quantified with Pierce BCA Assay kit (Thermo Fisher, 23225) and diluted in 10x NuPAGE sample reducing agent (Thermo Fisher Scientific, NP0009) and 4x NuPAGE LDS sample buffer (Thermo Fisher Scientific, NP0007) to be resolved on 8% or 10% Tris-Glycine gels in the presence of Tris-Glycine running buffer (3% Tris base, 14.4% Glycine, 1% SDS). Proteins were transferred for 1 h at 100 V on nitrocellulose membranes (Fisher Scientific, 10600003) and blocked in Tris-buffered saline supplemented with 0.1% Tween 20 (Sigma-Aldrich) (TBS-T) and 5% non-fat, dry milk. Membranes were probed with the following antibodies, diluted in TBS-T with 5% non-fat milk: SY0287 α-ATOH1 in-house antibody (0.07 μg/mL), ATOH1 (1:1000, Proteintech, 21215-1-AP), Vinculin (1:10,000, Sigma-Aldrich, V9264-100UL), Lamin B (1:1000, Abcam, ab229025), NEUROD1 (1:1000, Abcam, ab213725), MYC (1:500, Abcam, ab32072), SYP (1:10,000, Abcam, ab32127), YAP1 (1:1000, Abcam, ab52771), PLAU (1:1000, Abcam, ab24121). Membranes were incubated with the appropriate horseradish peroxidase-coupled secondary IgG (Agilent Technologies, P044801-2, P044701-2, P044801-2) for 1 h at room temperature in TBS and developed with Pierce SuperSignal West Pico PLUS Chemiluminescent Substrate (Thermo Fisher, 34580) or SuperSignal West Femto Maximum Sensitivity Substrate (Thermo Fisher, 34095) and either the BioRad ChemiDoc XRS+ System (BioRad, 1708265) or in dark room with developing films. Images from the BioRad ChemiDoc XRS+ System were analyzed using BioRad software Image Lab 3.0.1.

#### Automated immunostaining of CDX tissue

##### Immunohistochemistry (IHC)

Formalin-fixed paraffin-embedded (FFPE) CDX tumors and mouse livers and tissue specimens from the CHEMORES ethics were cut as 4 μm sections and stained by IHC for the following markers and antigen retrieval conditions: ATOH1 (1 μg/mL, ER2 10 min, Proteintech, 21215-1-AP), GFP (1:200, ER1 20 min, Cell Signaling Technologies, 2956), ASCL1 (1:250, ER1 20 min, BD Pharmigen, 556604), NEUROD1 (1:250, ER1 10 min, Abcam, ab213725). Anti-human mitochondria antibody (1:500, ER1 20 min, Abcam, ab92824) was used to detect human tumor cells in murine livers. IHC was performed using standard protocol F with Bond Polymer Refine Detection kit (Leica Biosystems, DS9800) on an automated BondMax or BondRX autostainers (Leica Biosystems). ATOH1 staining protocol was optimized on cell pellets and xenografts of ATOH1 KD CDX17P, HCC33 and Merkel Cell carcinoma human samples. For MYCL IHC staining, antigen retrieval was performed manually: tissue sections were rehydrated in water and antigen retrieval was performed in the Biocare Decloaking chamber (Biocare, DC2012) using Target Retrieval Solution, pH 6 (Agilent, S2369) at 110°C for 15 min; the slides were then cooled under running water for 10 min. IHC staining was then performed on a BondMax or BondRX autostainers with with Bond Polymer Refine Detection kit and using standard protocol F, excluding dewax and antigen retrieval steps, with MYCL1/L-Myc (Novus Biologicals, 25310002) diluted 1:2000.

### Quantification and statistical analysis

#### Flow cytometry data analysis

Flow cytometry data analysis was performed in FlowJo v10.8 Software (BD Life Sciences). For data obtained on BD FACSCanto II or BD LSRFortessa, cells were gated using FSC-A and SSC-A channels and single cells were gated using FSC-A and FSC-H channels. For data obtained on Novocyte, cells were gated using FSC-H and SSC-H channels and single cells were gated using FSC-A and FSC-H channels.

Early apoptosis was determined by single positivity to NucView 405 Caspase-3 Enzyme, necrosis by single positivity to LIVE/DEAD Fixable Far Red Dead Cell Staining or dual positivity. Cell death rate was calculated by summing both apoptosis and necrosis rates.

Cell cycle progression assessed by EdU incorporation was analyzed in gated single cells, by gating out possible apoptotic or dead cells in DAPI vs. SSC-H, characterised by low DAPI intensity. S phase was quantified as EdU+ cells. Cell cycle progression assessed by PI incorporation was analyzed with FCS Express v7 S phase quantified by MultiCycle cell cycle modeling in gated live, single cells.

Data reported in the Results and N numbers of independent biological repeats are reported in the appropriate legend for Figure 5: CDX17P, *N* = 4 Sh*Ren*, *N* = 3 ShATOH1#1 and #3; CDX30P, *N* = 5; HCC33, *N* = 2 ShRen, *N* = 3 Sh*ATOH1#1* and *#3* independent experiments. (E) Flow cytometry quantification of cell death after 14 days induction with DOX of ATOH1 KD, normalised as in D. Total cell death is reported as sum of apoptotic and necrotic cells. CDX17P: *N* = 4; CDX30P: *N* = 4 ShRen, *N* = 7 ShATOH1#1, *N* = 5 ShATOH1#3; HCC33: *N* = 2 ShRen, *N* = 3 ShATOH1#1 and *#3* independent experiments. (F) Same as E, reporting total Caspase-3 positive cells. All statistics in panel B are reported as two-tailed unpaired *t* tests across indicated conditions. (G) Flow cytometry quantification of cell death (as defined in E) after 7 days DOX-induction of ATOH1 KD in CDX17P. *N* = 3 independent experiments. *p* values are reported in C-G as per two-tailed unpaired *t* test. (H-I) ShATOH1#1 CDX17P (H) and CDX30P (I) cells were treated with (red) or without (black) DOX and with or without ferrostatin-1 (1μM), necrosulfonamide (NSA, 100 nM) or Z-VAD-FMK/Q-VD-OPh (20μM) and indicated combinations for 7 days. Cell viability was measured with CellTiter-Glo, normalized to vehicle treated, DOX-untreated cells and reported as fold change. Statistics in H-I are reported as per one-way ANOVA test with Dunnett’s test correction for multiple comparisons between DOX-treated conditions with and without programmed cell death inhibitors. Data are shown as mean ± SD.

#### Analysis of RNA-seq data

##### CDX studies

Alignment of RNA-seq data to Homo sapiens GRCh38 and Mus Musculus GRCm38 assembly (Ensembl release 99) was performed using nf-core RNA-seq pipeline 3.2,^107^ including STAR version 2.6.1d.^108^ In order to remove mouse contaminant reads, reads aligned to human GRCh38 were filtered using the bamcmp algorithm (version 2.0).^109^ Count matrices were generated from the filtered reads with the Rsubread package version 2.0.1.^128^ PCA was performed with prcomp within the R package “stats” on variance stabilising transformed (VST) data. In RNA-Seq upon ATOH1 KD in CDX17P, we found a batch effect linked to the processing date and this was removed with Limma v3.48.^110^ Differential expression analysis was performed on mouse-filtered count matrices with DESeq2 v1.32^129^ adjusting the *design* to account for confounding factors, such as batch effect (experimental date) and DOX treatment. For visualization and further analysis, log_2_ fold change was shrunk using the ‘apeglm’ transform^130^ within DESeq2 (v1.14). Differentially expressed genes were visualized with the EnhancedVolcano package v1.10^111^ and with the pheatmap package v1.0.12.^112^ GO enrichment analysis was performed with gProfiler2 v0.2.1^56^ and gene set enrichment analysis (GSEA) was performed using the Fast Gene Set Enrichment Analysis (FGSEA) package v1.18.^58^ All analyses were performed in R v4.1.0.

##### SCLC cell lines

RNA-Seq from SCLC cell lines is publicly available from The Broad Institute Cancer Cell Line Encyclopedia (CCLE) at https://portals.broadinstitute.org/ccle where transcript per million (TPM) counts were downloaded (file: CCLE_RNAseq_rsem_transcripts_tpm_20180929.txt.gz). TPM transcript counts were loaded in R v4.1.0 and target transcripts selected based on the most annotated transcript in Ensembl. Target transcripts were plotted with ggplot2 v3.3.5.^113^

##### SCLC limited stage tumors

Fragments per kilobase of exon per million mapped fragments (FPKM) normalized RNA-Seq from 81 surgically resected SCLC tumors is publicly available from ref. ^37^. FPKM gene counts were loaded in R v4.1.0 and plotted with ggplot2 v3.3.5^113^ and ggbeeswarm v0.6.0 for genes of interest.

##### Single cell RNA-Seq

Single cell RNA-Seq (scRNA-Seq) from SCLC biopsies and resections is publicly available from https://data.humantumoratlas.org,.^46^ Processed data were loaded in Python v3.6.12 and processed with algorithms published in the original article and available from https://github.com/dpeerlab/SCLC_atlas-HTAN.

#### Whole-exome sequencing

Whole-exome sequencing from CDX models was obtained and analyzed as previously described.^124^ Copy number for MYCL were reported as copy number ratio, Log_2_(CNV/2).

#### ChIP-seq bioinformatic analysis

Alignment of ChIP-Seq data to Homo sapiens GRCh38 and Mus Musculus GRCm38 assembly (Ensembl release 99) was performed using nf-core ChIP-seq pipeline 1.2.1,^107^ including Burrows-Wheeler Aligner (BWA) v0.7.17-r1188.^114^ Mouse contaminant reads were removed using the bamcmp algorithm (version 2.0).^109^ Because mouse reads only accounted for ∼ 2% of the reads, we performed the analysis on count matrices derived from alignment to GRCh38, without further filtering. Peak calling was performed within nf-core ChIP-seq pipeline 1.2.1 with MACS2 v2.2.7.1^131^ with the following: nextflow run nf-core/chipseq --genome GRCh38_v99 --macs_gsize 2.7e9 --narrow_peak. Quality control was performed with MultiQC v1.9.^115^ Normalised bigWig files were scaled to 1 million mapped reads with BEDTools v2.29.2^116^ for visualization purposes on Integrative Genomics Viewer (IGV) and were used to generate gene-body meta-profiles with deepTools v3.4.3.^117^ Differential binding analysis was performed with DESeq2^129^ within DiffBind v3.2.7^132^ between ATOH1-competent and depleted conditions. Correlative heatmaps were obtained with dba.plotHeatmap function within DiffBind and plotted with pheatmap v1.0.12^112^ for visualization purposes. Principal component analysis (PCA) plots were obtained with dba.plotPCA and volcano plots obtained with dba.plotVolcano within DiffBind. Binding sites overlap across conditions was obtained with dba.plotVenn function. Results of differential binding analysis were exported with dba.report specifying method = DBA_DESEQ2 and differentially bound (DB) peaks annotated and plotted with ChIPSeeker v1.28.3.^118^ Gene ontology (GO) enrichment analysis was performed on genes annotated on DB peaks with ClusterProfiler v4.0.5.^119^ ChIP-Seq profile over consensus peak sets for each ATOH1 antibody was obtained with the function BamBigwig_to_chipProfile and generateEnrichedHeatmap within profileplyr v1.8.1.^120^ Motif enrichment analysis was performed on 500 bp FASTA sequences, centered on peak summit, annotated with bedtools v2.27.1–7 getfasta function, with MEME ChIP (https://meme-suite.org/meme).^121^ All analyses were performed in R v4.1.0.

#### Integration of ChIP-Seq and RNA-Seq with BETA

ChIP-Seq and RNA-Seq from ATOH1-competent and depleted cells were integrated with Binding and Expression Target Analysis (BETA, v.1.0.7^57^) to identify direct transcriptional targets. BETA was run on bed files from differentially bound (DB) peaks, including false discovery rate (FDR) and DGEA upon ATOH1 depletion included log_2_ fold change and p adjust obtained from DESeq2 analysis. BETA was run in basic function with the following parameters: --gname2 -k O --info 1,2,3 --method score -g hg38 --pn 17737 -d 10000 --df 0.01 --da 1. In this way we have considered all peaks (--pn 17737), *p* value cutoff of 0.01 for DGEA and a 10 kB distance from transcription start site (TSS).

#### IHC analysis

Whole IHC slides were scanned using a Leica SCN400 or OLYMPUS VS200 and whole IF slides were scanned on Olympus VS120. IHC was analyzed with HALO Image analysis software (Akoya Biosciences). Regions of tissue to be analyzed were annotated and classified using a random forest tissue classifier into tumor, stroma and necrotic areas. Areas of tumor were annotated for further analysis and nuclei within the tumor were detected based on size, shape and haematoxylin staining. Tumor cells were scored as either positive or negative based on the staining intensity threshold within the cytoplasm (GFP, human mitochondria) or nucleus (ATOH1, ASCL1, NEUROD1). The percentage of positive tumor cells was exported for all analyses. In patient samples, the threshold of expression defining marker positivity (Figure 2) was >1.5% positive tumor cells for ASCL1 and NEUROD1 and >5% positive tumor cells for ATOH1 in *N* = 102 independent samples. Whole sections were scored for *N* = 2 independent CDX tumors for IHC reported Figure 1. Metastatic dissemination to the liver was detected with an anti-human mitochondria antibody and reported as percentage of murine liver area (Figure 6I).

#### *In vivo* analysis

Cisplatin/etoposide treatment of *N* > 3 independent biological repeats was carried out in *N* = 29 CDX and statistical analysis performed with Fisher’s exact test between ATOH1 CDX and the remaining CDX (Figure 1I and Results).

#### Effect of ATOH1 knockdown *in vivo* after resection

CDX17P ShRen and ShATOH1#3 (ShATOH1) were injected s.c. in NSG mice and left for 19 days to allow for tumor establishment. After 19 days, mice were fed either standard diet (control arms, *N* = 3) or DOX-supplemented feed (experimental arms, *N* = 15) and s.c. tumor growth was assessed. S.c. tumors were surgically resected when at 500-800 mm^3^ to allow for metastatic dissemination and mice were kept on study for 28 days or until s.c. tumor reached maximum size, whichever came first. (B) S.c. tumor growth curves, from day of first tumor measurement to s.c. tumor resection (see methods), of mice implanted with ShRen and ShATOH1 and fed DOX-supplemented diet. Key: black, ShRen fed DOX-diet; red, ShATOH1#3 fed DOX-diet. *N* = 15 mice per cohort; data reported as mean ± SD. Dotted lines indicate when tumors from each cohort reached 500 mm^3^: ShRen, 14 ± 3 days; ShATOH1, 21 ± 5 days. (C) Quantification of the slope of tumor growth curves in B. Key: same as in B; shades of gray for control cohort fed standard diet for the duration of the study. *p* values were calculated with ANOVA test and slope of the curve was reported as mean ± SD for each cohort. (D) Kaplan-Meier curve of time to surgical resection of s.c. tumor or maximum 800 mm^3^ for inoperable tumors. Control arms, fed a standard diet, reported in scales of gray. *p* values were calculated with Log rank Mantel-Cox test. (E) Quantification of metastatic dissemination to the liver in *N* = 3 mice fed standard diet, *N* = 5 Sh*Ren*- and *N* = 15 Sh*ATOH1*-tumour bearing mice fed DOX-diet that underwent surgical resection of s.c. tumor and survived on study for at least 22 days after resection. Data is shown as percentage of animals displaying metastatic dissemination (disseminated tumor cells and micro/macro-metastases, in red) or no metastatic dissemination in the liver (blue). Metastases were identified based on human mitochondria staining. (F) (see IHC method analysis) and: Quantification of GFP (G) and ATOH1 (H) IHC staining in metastases from *N* = 2 DOX-untreated ShRen, *N* = 3 DOX-untreated ShATOH1#3, *N* = 4 ShRen DOX-fed, *N* = 6 ShATOH1#3 DOX-fed mice. Data are shown as geometric mean ± geometric SD. *p* values are reported as per two-tailed unpaired Mann Whitney U test.

#### Effect of ATOH1 knockdown *in vivo* after intracardiac implantation

Prior to cell implantation, ATOH1 depletion was induced by DOX treatment for 4 days *in vitro*, followed by sorting GFP-positive, viable cells by flow cytometry. Untreated control cells were sorted exclusively for viable cells. Animals in the DOX treatment cohorts were fed a DOX-supplemented diet 24 h prior to implantation and they were kept on that diet until endpoint. Animals in the uninduced control groups were given a standard diet. Animals from all 4 cohorts (ShRen +/− DOX and ShATOH1 +/− DOX) were removed at the onset of symptoms (i.e., distended abdomen, detailed in methods) or after 70 days. (J) Kaplan-Meier curve of time to sacrifice. Control cohorts, fed a standard diet, reported in scales of gray. *p* values were calculated with Log rank Mantel-Cox test. (K) Quantification of metastatic dissemination to the liver for each cohort. Data is shown as per Figure 6D. (L) Quantification of metastatic cells in the liver for each cohort. Metastatic cells were identified based on human mitochondria staining. Data shown as mean ± SD. *p* values were calculated with a two-tailed unpaired Mann Whitney U test. (M-N) Quantification of GFP (M) and ATOH1 (N) IHC staining in metastases from *N* = 5 DOX-untreated ShRen, *N* = 5 DOX-untreated ShATOH1, *N* = 5 ShRen DOX-fed, *N* = 1 ShATOH1#3 DOX-fed mice. Data are shown as geometric mean ± geometric SD. No statistical test could be performed as ShATOH1 contained only one value.

#### Sequences and primers

##### ATOH1 antibody production and cloning

###### Codon-optimized ATOH1 DNA sequences

**Table:** 

ATOH1 full length	ATGAGCCGTCTGCTGCATGCCGAAGAATGGGCTGAAGTTAAAGAACTGGGTGATCATC ATCGTCAGCCGCAGCCGCATCATCTGCCGCAGCCTCCGCCTCCTCCTCAGCCTCCTGC AACACTGCAGGCACGTGAACATCCGGTTTATCCGCCTGAACTGAGCCTGCTGGATAGC ACCGATCCGCGTGCATGGCTGGCACCGACGCTGCAGGGTATTTGTACCGCACGTGCAG CACAGTATCTGCTGCACAGTCCGGAACTGGGAGCAAGCGAAGCAGCAGCACCGCGTG ATGAAGTTGATGGTCGTGGTGAACTGGTTCGTCGTAGCAGCGGTGGTGCAAGCAGCA GTAAAAGCCCTGGTCCGGTTAAAGTTCGTGAACAGCTGTGTAAACTGAAAGGTGGTGT TGTTGTTGATGAACTGGGTTGTAGCCGTCAGCGTGCACCGAGCAGCAAACAGGTTAA TGGTGTTCAGAAACAGCGTCGTCTGGCAGCAAATGCCCGTGAACGTCGTCGTATGC ATGGTCTGAATCATGCATTTGATCAGCTGCGTAATGTTATCCCGAGCTTCAACAAT GATAAAAAACTGAGCAAATATGAAACCCTGCAGATGGCCCAGATTTATATCAAT GCACTGAGCGAACTGCTGCAGACCCCGAGTGGTGGTGAACAGCCTCCTCCGC CACCGGCAAGCTGTAAAAGCGATCATCACCATCTGCGTACCGCAGCAAGCTA TGAAGGTGGTGCAGGTAATGCAACCGCAGCCGGTGCACAGCAGGCAAGCGGT GGTAGCCAGCGTCCGACACCGCCTGGTAGCTGTCGTACCCGTTTTAGCGCACC GGCATCAGCCGGTGGTTATAGCGTTCAGCTGGATGCACTGCATTTTAGCACC TTTGAAGATAGCGCACTGACCGCAATGATGGCACAGAAAAATCTGAGCCCGA GCCTGCCAGGTAGCATTCTGCAGCCGGTTCAAGAAGAAAATAGCAAAACC AGTCCGCGTAGCCATCGTAGTGATGGTGAATTTTCACCGCATAGCCATTAT AGCGATAGTGATGAAGCAAGC

###### Recombinant ATOH1 sequencing and amplification primers

**Table:** 

Name	Sequence	Purpose
AC_ATOH1_FRW-2	TGGTGGACAGCAAATGGGTCGC GGATCCATGAGCCGTCTGCTGCATG	PCR-amplification of ATOH1-codon optimized for Ab production (Ta = 66)
AC_ATOH1_REV-2	GGTGGTGCTCGAGTGCGGCCGCGC TTGCTTCATCACTATCGCTATAATG	PCR-amplification of ATOH1-codon optimized for Ab production (Ta = 66)
T7-terminal-REV	GCTAGTTATTGCTCAGCGG	Sequencing primer for PET28A and PET28A-ATOH1
T7-FRW	TAATACGACTCACTATAGGG	Sequencing primer for PET28A-ATOH1
LacI-FRW	ACACCATCGAATGGCGCAA	Sequencing primer for PET28A
mir-E CTRL primer	TGTTTGAATGAGGCTTCAGTAC	sequencing primer for mir-E constructs

###### ATOH1 knockdown constructs

**Table:** 

Name	Sequence	
shATOH1#1	TGCTGTTGACAGTGAGCGAAGCGATGATGGCGCAAAAGAATAGTGAAGCCACAGATGTATTCTTTTGCGCCATCATCGCTG TGCCTACTGCCTCGGA	
shATOH1#3	TGCTGTTGACAGTGAGCGCCAACGACAAGAAGCTGTCCAATAGTGAAGCCACAGATGTA TTGGACAGCTTCTTGTCGTTGTTGCCTACTGCCTCGGA	

## Supplemental information

Document S2. Data S1–S10

Table S4. Clinical characteristics of the 104 patient-derived biopsies used for IHC profiling, related to Figure 2B

Table S5. IHC positivity of clinical samples, related to Figure 2C

Table S6. Differentially bound ATOH1 ChIP-seq peaks obtained with DiffBind, related to Figure 3F

Table S7. KEGG and GO enrichment analysis of ATOH1 differentially bound genes, related to Figures S3E and S3F

Table S8. DGEA upon ATOH1 depletion in CDX17P (positive values indicate genes upregulated upon ATOH1 depletion), related to Figure 4A

Table S9. GO enrichment analysis of genes downregulated (blue) and upregulated (red) upon ATOH1 depletion in CDX17P, related to Figure 4B

Table S11. Gene signatures used for GSEA, related to Figures S4A–S4D

Table S12. ATOH1 direct targets as determined by BETA, related to Figure 4C

Table S13. GO enrichment analysis of ATOH1 direct targets, related to Figure 4D

Table S14. DGEA in ATOH1 CDX vs. the rest of the biobank (positive values indicate enrichment in ATOH1 CDX), related to Figure 4F

Table S15. ASCL1 and NEUROD1 gene signatures, related to Figures S4H and S4I Obtained from Borromeo et al. and Ireland et al.

Document S3. Article plus supplemental information
