# Supplementary material for: Functional characterization of the ATOH1 molecular subtype indicates a pro-metastatic role in small cell lung cancer
Source: Cell Rep. 2025 Apr 29;44(5):115603. doi: 10.1016/j.celrep.2025.115603 (PMC12116416; doi:10.1016/j.celrep.2025.115603)
Supplement: Document S1. Figures S1–S6 and Tables S1–S3, S10, and S16 [file mmc1.pdf]

Cell Reports, Volume *44*

Supplemental information

Functional characterization of the ATOH1 molecular subtype indicates a pro-metastatic role in small cell lung cancer

Alessia Catozzi, Maria Peiris Pagès, Sam Humphrey, Mitchell Revill, Derrick Morgan, Jordan Roebuck, Yitao Chen, Bethan Davies-Williams, Kevin Brennan, A.S. Md. Mukarram Hossain, Vsevolod J. Makeev, Karishma Satia, Pagona P. Sfyri, Melanie Galvin, Darryl Coles, Alice Lallo, Simon P. Pearce, Alastair Kerr, Lynsey Priest, Victoria Foy, Mathew Carter, Rebecca Caeser, Joseph M. Chan, Charles M. Rudin, Fiona Blackhall, Kristopher K. Frese, Caroline Dive, and Kathryn L. Simpson

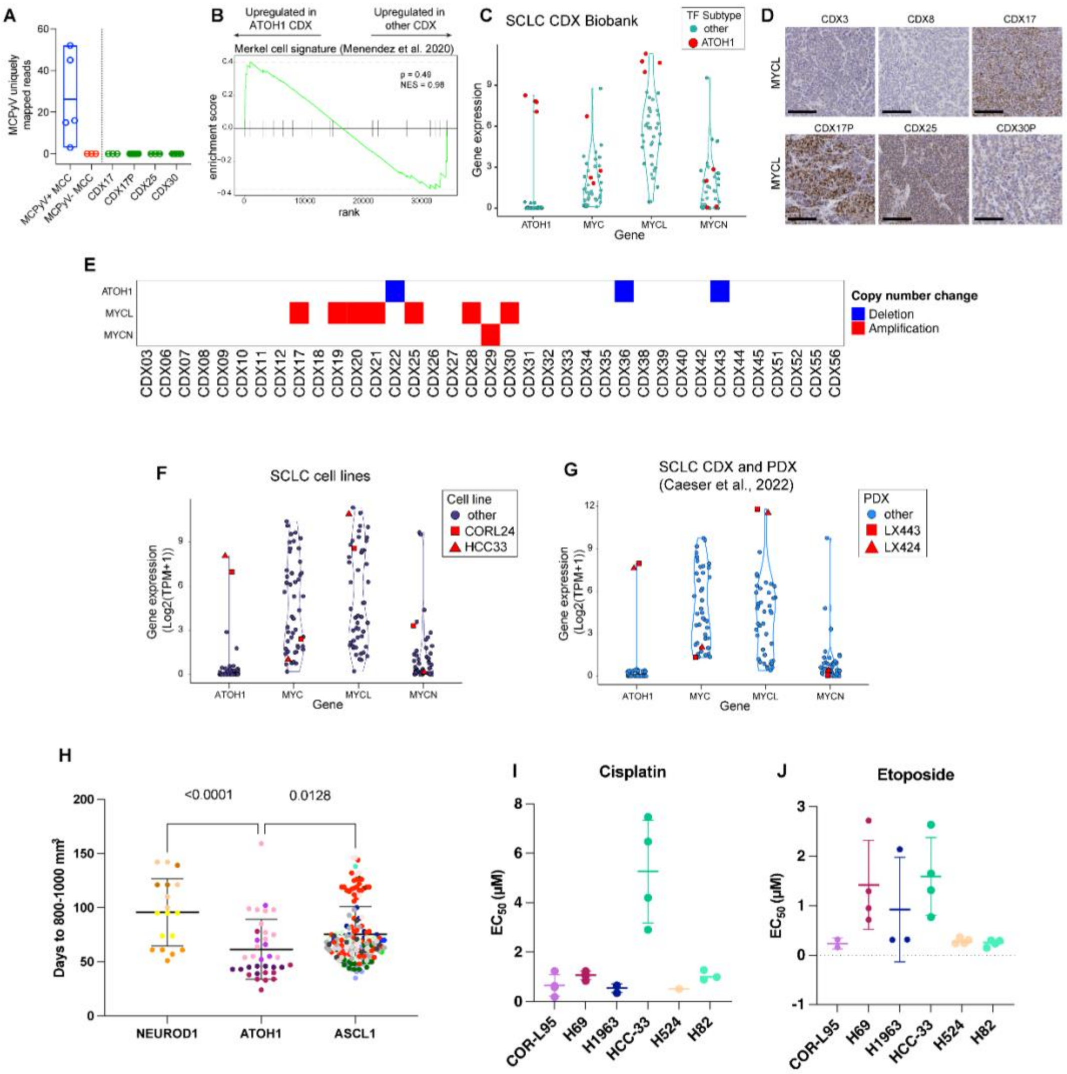

Supplemental Figure S1. ATOH1 CDX do not have MCC origin and present high expression of MYCL. Relative to Figure 1. (A) Detection of Merkel cell polyoma virus (MCPyV) transcripts in positive and negative control human Merkel cell carcinoma (MCC) samples (PRJNA775071) and ATOH1 CDX. (B) Gene set enrichment analysis (GSEA) for a Merkel cell gene signature from Menendez *et al*.[S1] in ATOH1 CDX (N=4) compared to the whole biobank (N=35). GSEA was performed with Fgsea (http://bioconductor.org/packages/fgsea/). (C) Violin plot of expression of indicated *MYC* family genes in the SCLC CDX biobank (N=39). ATOH1 subtype samples and preclinical models highlighted in red. (D) Representative IHC images for MYCL in SCLC-A CDX3, SCLC-N CDX8 and ATOH1 CDX CDX17, 17P, 25 and 30P. (E) Heatmap representing copy number loss and amplification for ATOH1, MYCL and MYCN in SCLC CDX. Copy number ratios were inferred using CNVKit from the whole-exome sequence data of CDX models. Deletion: log2 ratio < log2(0.5); amplification: log2 ratio > log2(2). (F-G) Violin plot of expression of indicated *MYC* family genes in SCLC cell lines[S2](F) and SCLC PDX[S3] (G) from publicly available datasets. ATOH1 subtype preclinical models highlighted in red and annotated by shape as in legend. (H) CDX models were implanted subcutaneously in immunocompromised mice and time for the tumour to reach 800-1000 mm3 assessed. Data are shown divided by CDX subtype. Individual tumours are shown, coloured by CDX model, NEUROD1 models: CDX08, brown, CDX08P, orange, CDX21, light orange, CDX29, yellow, ATOH1 models: CDX17, pink, CDX17P, raspberry, CDX25, dark purple, CDX30, rich mauve, ASCL1 models: CDX3, blue, CDX3P, dark blue, CDX20, light blue, CDX 18, grey, CDX18P, dark green, CDX28, bright green, CDX42, turquoise, CDX42P, barbour, CDX22, maroon, CDX51, red, CDX52, black, CDX26, silver, CDX32, salmon pink. (I-J) Response to cisplatin (I) and etoposide (J) was evaluated in SCLC cell lines belonging to different molecular subtypes (SCLC-A: COR-L95, H69 and H1693; NEUROD1: H524, H82; ATOH1: HCC-33). Sensitivity is reported as EC50 after 5 days treatment. Data are shown as mean ± SD; P values are shown as per non-parametric one-way anova followed by Dunnett's multiple comparisons test.

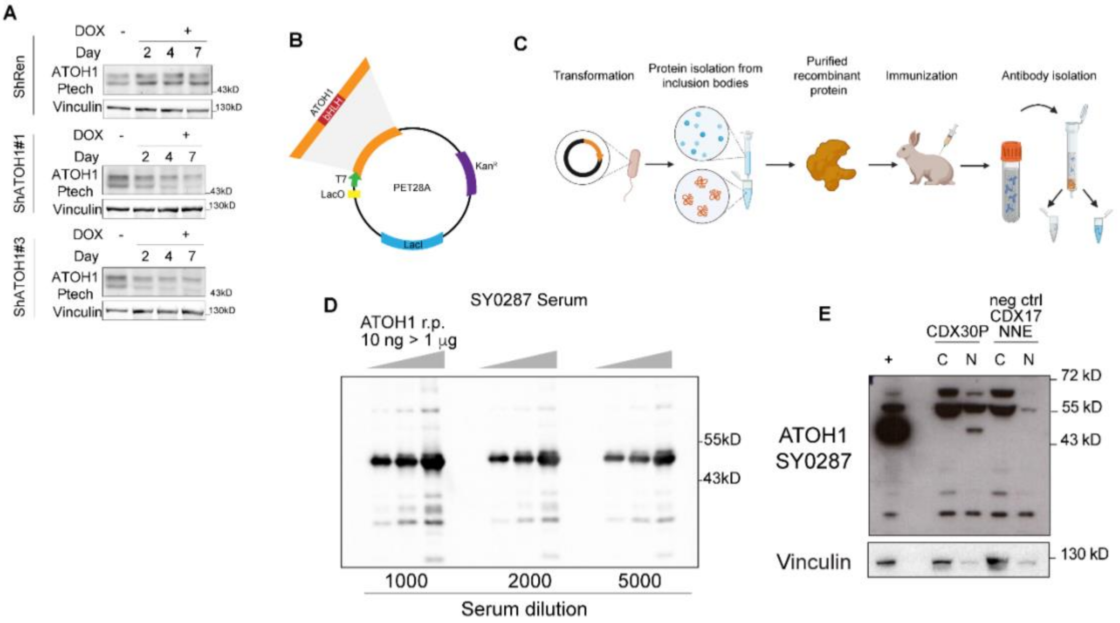

Supplemental Figure S2. ATOH1 antibody production and validation. Relative to Figure 2. (A) Western blot showing ATOH1 expression detected by the Ptech antibody over a time-course (0 to 7 days) of ATOH1 knockdown (KD) induction with doxycycline (DOX) in CDX17P. ShRen served as control for ATOH1 KD and Vinculin served as loading control. Western blots are representative of N=2 independent experiments. (B) Schematic of plasmid construct to express ATOH1 recombinant protein in IPTG-inducible PET28A system. (C) Workflow to produce the in-house antibody: ATOH1 recombinant protein was purified from bacterial culture and used for immunization of one rabbit. Polyclonal antibodies were isolated from final bleed serum by affinity purification. (D) Test of SY0287 serum before affinity purification against increasing amounts of ATOH1 recombinant protein (10 ng, 100 ng and 1 mg) by western blot. (E) Validation of ATOH1 detection by nuclear (N) and cytoplasmic (C) fractionation of CDX30P (positive control) and CDX17 Non-NE cells (Negative control). Transient ATOH1 overexpression in LentiX 293T cells (indicated as +) served as positive control for detection.

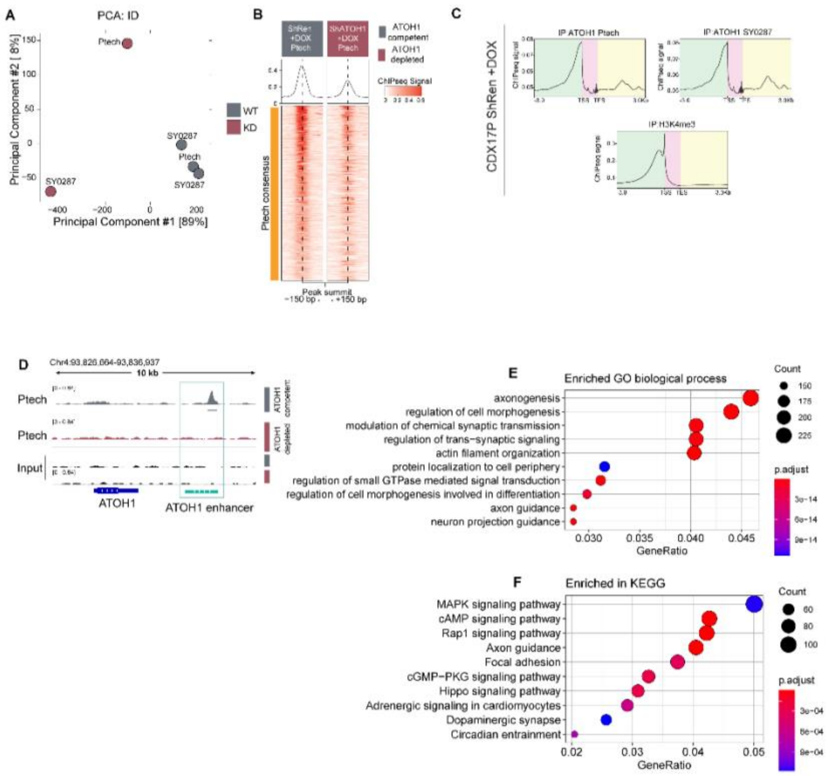

Supplemental Figure S3. ChIP-Seq samples cluster based on ATOH1 competency and ATOH1 binds to its own enhancer. Relative to Figure 2. (A) Principal component analysis (PCA) of ChIP-Seq samples where ATOH1 competent samples (grey, WT) cluster together and away from ATOH1-depleted samples (red, KD). (B) Heatmap of ChIP-Seq signal for consensus peak sets of Ptech in ATOH1 competent (grey) and depleted (red) CDX17P, generated with the generateEnrichedHeatmap function within profileplyr v1.8.1 (https://www.bioconductor.org/packages/devel/bioc/vignettes/profileplyr/inst/doc/profileplyr.html).
(C) Metagene analysis of ATOH1 (detected with Ptech and SY0287) and H3K4me3 ChIP-Seq signal generated with deepTools[S4]. Key: green, upstream of gene body; pink, gene body; yellow, downstream of gene body. (D) ATOH1 binding peaks at the ATOH1 locus and its downstream enhancer (shown in light green) as detected by the Ptech antibody. The peaks were visualized with the Integrated Genomics Viewer genome browser. (G-H) Gene ontology (GO) biological process (G) and KEGG (H) enrichment analysis of differentially bound ATOH1 peaks identified Figure 3F. Analysis was performed with gage[S5].

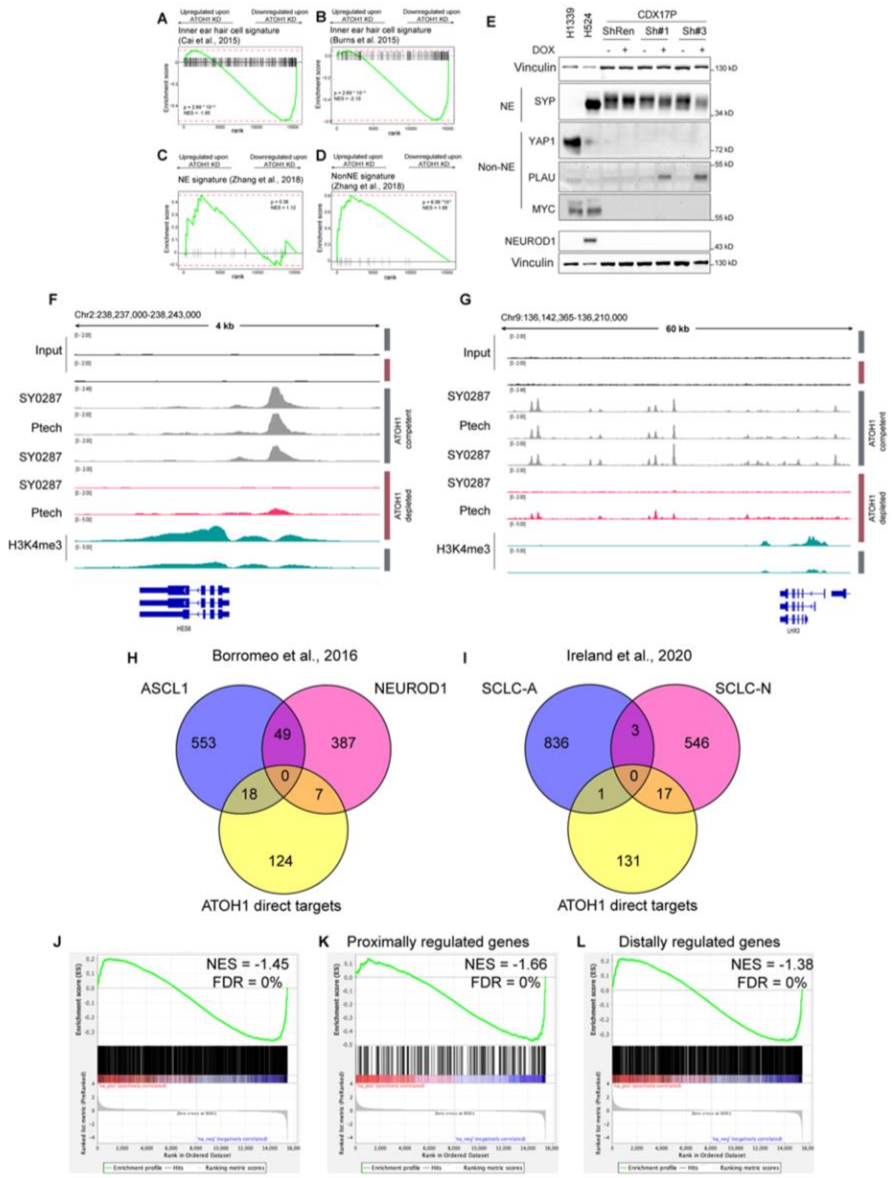

Supplemental Figure S4. ATOH1 direct targets identified in CDX17P are upregulated in ATOH1 CDX. Relative to Figure 4. (A-B) Gene set enrichment analysis (GSEA) for inner ear hair cell gene signatures obtained from Cai *et al*., (2015)[S6] (A) and Burns *et al*., (2015)[S7] (B) upon ATOH1 depletion in CDX17P, performed with opensource software Fgsea (http://bioconductor.org/packages/fgsea/). (C-D) GSEA for NE (C) and Non-NE (D) gene signatures obtained from Schenk *et al*., (2021)[S8]. NES: normalized enrichment score. (E) Western blot expression of NE marker SYP and NonNE markers YAP1, MYC and PLAU after 14 days of ATOH1 knockdown (KD) induction with doxycycline (DOX) in CDX17P. ShRen served as control for ATOH1 KD; H1339 and H524 served as positive controls for expression of YAP1 and MYC; Vinculin served as loading control. Western blots are representative of N=2 independent experiments. (F-G) ATOH1 binding peaks at HES6 promoter (F) and LHX3 locus and downstream (G) as detected by the Ptech and SY0287 antibodies. The peaks were visualized with the Integrated Genomics Viewer genome browser. (H-I) Venn diagrams showing overlap between ATOH1 direct targets and ASCL1 and NEUROD1 targets from Borromeo *et al*., (2016)[9] and Ireland *et al*., (2020)[10]. (J) GSEA of the genes associated with the top 10% of ATOH1-specific peaks, ranked by differential expression upon ATOH1 depletion. (K-L) GSEA of the genes associated with the top 10% of ATOH1 differentially bound peaks, divided by genes regulated proximally (J) or distally (K), based on the distance of the closest ATOH1 binding event (□ 5 kB from TSS or >5 kB from TSS) ranked by differential expression upon ATOH1 depletion. NES = Normalised Enrichment Score; FDR = False Discovery Rate.

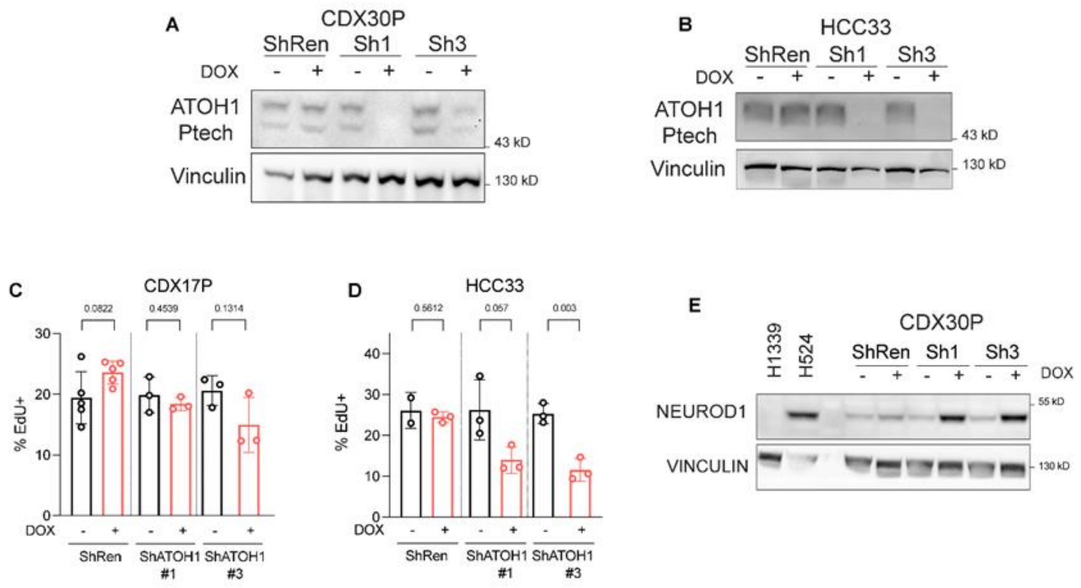

Supplemental Figure S5. ATOH1 knockdown in CDX17P, CDX30P and HCC33. Relative to Figure 5. (A-B) Representative western blot for ATOH1 in CDX30P (A) and HCC33 (B) cells transduced with ShRenilla (ShRen) and ShATOH1#1 (Sh1) and #3 (Sh3) and treated with DOX for 7 days. (C-D) Bar plot of percentage of cells in S phase, as identified by EdU incorporation, in CDX17P (C) and HCC33 (D) upon ATOH1 depletion. Statistics are reported as two-tailed unpaired *t* test between DOX untreated and treated condition. (E) Representative western blot for NEUROD1 in CDX30P cells transduced with ShRenilla (ShRen) and ShATOH1#1 (Sh1) and #3 (Sh3) and treated with DOX for 7 days.

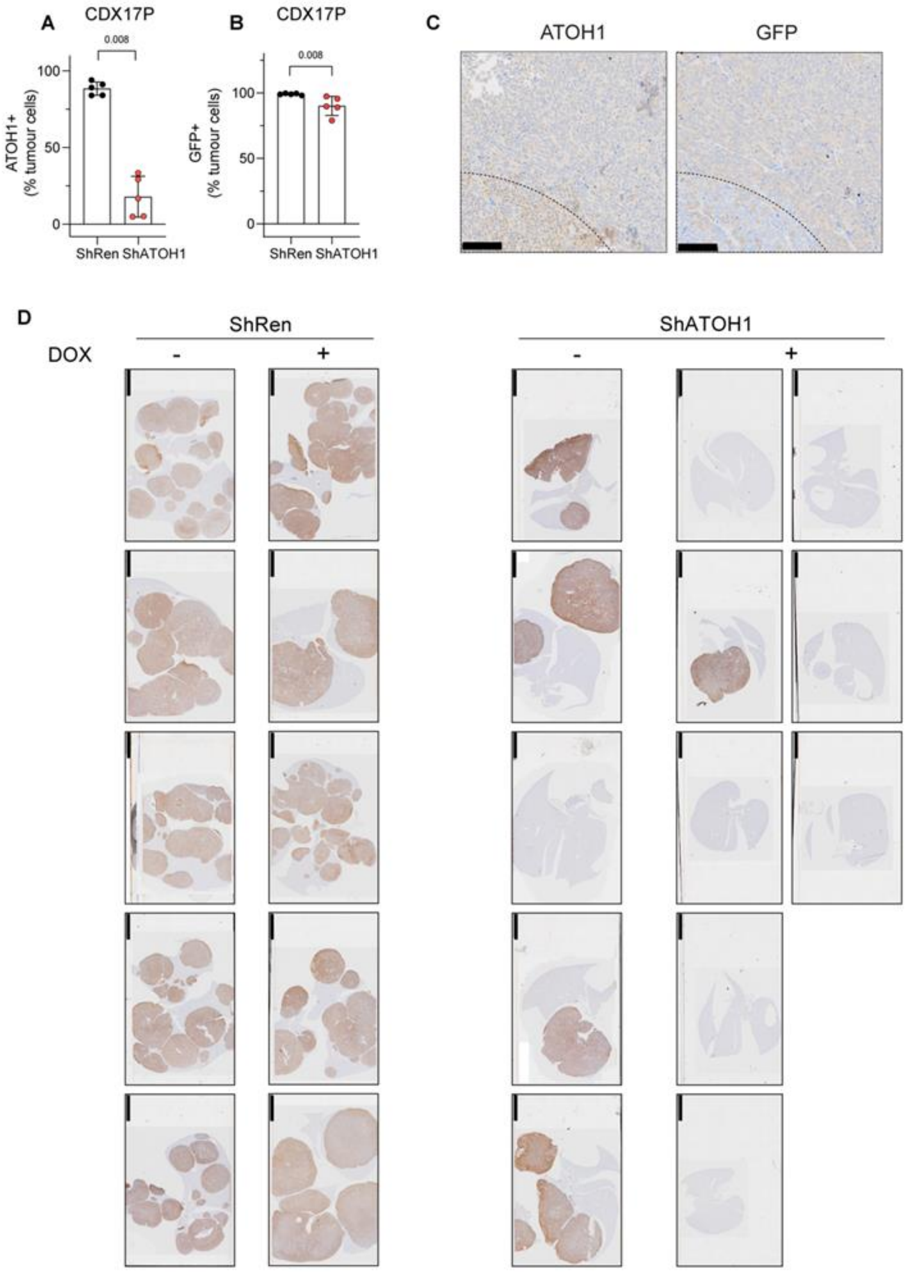

Supplemental Figure S6. Heterogeneous GFP and ATOH1 expression in ATOH1 KD subcutaneous tumours. ATOH1 KD cells exhibit reduced metastatic ability. Relative to Figure 6. (A-B) Quantification of ATOH1 (A) and GFP (B) IHC staining in N=5 subcutaneous tumours from mice implanted with either ShRen or ShATOH1 cells and fed DOX-supplemented diet. KD cohort highlighted in red. Statistics reported as per two-tailed unpaired Mann Whitney U test. (C) Representative images of ATOH1 and GFP IHC staining in consecutive sections highlighting parts of tumours negative for GFP and positive for ATOH1 (dotted lines). Scale bars: 100 mm. (D) IHC staining of human mitochondria in livers from animals that underwent intracardiac implantation of ShRen cells and fed a standard diet (-DOX, N=5) or a DOX-supplemented diet (+ DOX, N=5) or ShATOH1 cells and fed a standard diet (-DOX, N=5) or a DOX-supplemented diet (+DOX, N=8). Only one animal in the ATOH1 KD cohort developed metastasis in the liver. Scale bars: 5 mm.

Supplemental Tables

Table S1. Clinical characteristics of patients donor for ATOH1 CDX and PDX, related to Figure 1A

CDX/PDX model	Gender	Age at diagnosis	SCLC stage	Site of metastases	First line of therapy	Cycles	Chemosensitivity status	Assessment	OS (months)
CDX17/17P	Female	60	ES	Nodes, Liver	Carboplatin/Etoposide	4	Progressive	Clinical symptoms	6.3
CDX25	Male	80	ES	Nodes, Liver	Carboplatin	1	Progressive	N/A	2.4
CDX30P	Female	63	ES	Liver	Carboplatin/Etoposide	4	Progressive	Liver progression	13
LX424/443	Male	68	ES	Nodes, bones	Cisplatin/etoposide	2	Progressive	Bilateral adrenal metastases, four brain lesions	14

*chemosensitivity status is assessed within 3 months of the end of the last cycle of chemotherapy. Progression of the disease is assessed radiogically or clinically.

Table S2. Merkel cell gene signature from Menendez et al., 2020, related to Figure S1B

merkel_cells_Menendez2020 gene signature
CAV2
LHX2
APOE
HSPB1
CD44
SYN2
GSDMA
KRT27
KRT10
GNG4
KCNV1
TP63
PLEC
WNT10B
COL17A1
AVIL
PKP1
LAMB3
TRIM29
THSD4
GC
KRT17
KRT33A
PPP1R16B
LMX1B
PPL
DRGX
MUC1
PENK
SLC35D3
SV2C
TCHH
LGALS7
LGALS7B
TMEM158
CDH3
KRT79
KRT5
SERPINB5
DSG1
AHNAK
PHLDB3
FAM83C
CCER2

Table S3. pRECIST scores for CDX after 1 cycle of chemotherapy in vivo, related to Figure 1I		
**CDX**	**TF_subtype**	**pRECIST**
CDX12	ASCL1	SD
CDX17	ATOH1	PD1
CDX17P	ATOH1	PD1
CDX2	ASCL1	PR
CDX23	ASCL1	PD1
CDX25	ATOH1	PD1
CDX30P	ATOH1	PD1
CDX31P	ASCL1	PD1
CDX32P	ASCL1	SD
CDX34	ASCL1	SD
CDX35	ASCL1	SD
CDX38	ASCL1	SD
CDX4	ASCL1	PD1
CDX42	ASCL1	PR
CDX42P	ASCL1	CR
CDX7	ASCL1	CR
CDX8	NEUROD1	PR
CDX8P	NEUROD1	SD
CDX10	ASCL1	PR
CDX14P	ASCL1	PR
CDX15P	ASCL1	PD2
CDX15PP	ASCL1	PR
CDX22P	ASCL1	PR
CDX26	ASCL1	PD2
CDX3	ASCL1	MCR
CDX39P	ASCL1	SD
CDX3P	ASCL1	PR
CDX41P	ASCL1	PD2
CDX44P	ASCL1	SD
CDX9	ASCL1	SD
CDX18	ASCL1	SD
CDX18P	ASCL1	SD
CDX20	ASCL1	PD2
CDX20P	ASCL1	PD2
CDX33	ASCL1	SD
CDX40	ASCL1	SD
CDX45	ASCL1	CR

Table S10. Results of gene set enrichment analysis performed with fgsea upon ATOH1 depletion in CDX17P, related to Figure S4A-D

pathway	pval	padj	log2err	ES	NES	size	leading_edge
**NE_signature_Cai et al., 2021**	0.29566855	0.344946642	0.105920293	0.462799619	1.132097155	24	CHGB, SEZ6, SYN1, RUNDC3A, CELF3, RTN1, SH3GL2
**NonNE_signature_Cai et al., 2021**	2.37E-05	6.64E-05	0.575610261	0.815149599	1.994013185	24	PLAU, S100A10, SLC16A5, S100A16, TACSTD2, ITGB4, ANXA1, MYOF, LGALS3, IFITM3, ABCC3, ARHGDIB, EMP1, PTGES
**hair_cells_Burns et al. 2015**	1.00E-10	3.50E-10	NA	-0.598172416	-2.11868424	269	ATOH1, DLL1, CLEC18A, RASD2, INSC, CLEC18B, LHX3, CLEC18C, USH2A, IRX2, BDNF, MNS1, CCDC141, RBM24, B3GNT4, C12orf56, CFAP77, MYO15A, ESPNL, AK9, EFCAB10, PLAAT1, CFAP54, DYDC2, PLK4, GRXCR1, LMLN, PAX2, CDC25B, EFCAB6, LRRC73, CFAP299, CHRNG, IQUB, TMPRSS3, SMIM18, FAM166C, HYDIN, ELMOD1, IQCA1, KIF9, MAK, DNAH7, RUNX1T1, ABCD2, C17orf97, NEK5, MAPK15, NEURL1B, MYCL, CFAP43, CDH23, CLRN1, KCNH6, CXCL14, CFAP69, FANK1, AKNAD1, LNX2, CASZ1, MINDY4B, DNAL1, BBOF1, LRGUK, DLK2, C2orf81, ARMC9, CCDC113, SLC52A3, CFAP65, FAM183A, RAB3B, ENO4, KIF24, POU4F3, SRRM4, LRRIQ1, DYTN, AK8, ADAMTS19, DNAAF4, NCALD, NME9, FHAD1, PIH1D2, EFHC1, TNFAIP8L1
**hair_cells_Cai et al. 2015**	1.00E-10	3.50E-10	NA	-0.529970354	-1.961298532	419	ATOH1, DLL1, CLEC18A, RASD2, HES6, INSC, CLEC18B, LHX3, CLEC18C, TBC1D2, USH2A, DLL3, BDNF, WWC1, RBM24, B3GNT4, ST8SIA3, TCERG1L, MREG, C12orf56, KCNMB2, DLL4, RNF182, LRRC43, FAM160A1, BARHL1, LMO7, ESPNL, MFNG, PLS1, EFCAB10, ACTN3, RSPH1, NOD2, GRXCR1, TMEM184A, PSAT1, PRPH2, ARMC4, SYT17, PAX2, OXTR, EFCAB6, CIB2, SALL3, ACBD7, LFNG, TTC25, CHRNG, IQUB, UGT8, FAM166C, IQCG, SALL1, HYDIN, C9orf116, MYO16, ELMOD1, MYO6, IGFBPL1, PPIL6, CBLN1, ICA1, PLEKHF2, MTHFD2, JAKMIP1, ABCD2, C17orf97, ENKUR, NEK5, NEURL1B, ATP7B, GPR4, GCK, FGF5, KCNH6, CXCL14, FANK1, AKNAD1, LNX2, NEUROD6, DNALI1, LHFPL4, LRGUK, DLK2, RIMBP2, EMX2, FBXO36, CADPS2, ZDHHC23, LRRC26, NACA, RAB3B, KIAA1549L, POU2AF1, ENO4, KIF5C, HEBP2, POU4F3, BEX2, CHRNA1, SRRM4, LRRIQ1, PCSK1, HES5

Table S16. Šídák's Multiple Comparisons test applied to cell viability data from ATOH1 KD +/- apoptosis, pyroptosis, necroptosis or ferroptosis inhibitors, related to Figure 5H-I

CDX17P

Šídák's multiple comparisons test	Mean Diff.	95.00% CI of diff.	Summary	Adjusted P Value
Fe-1 vs. +DOX Fe-1 1 uM	0.4475	-0.04356 to 0.9385	ns	0.0649
NSA vs. +DOX NSA 100 nM	0.4358	0.02538 to 0.8462	*	0.0422
Z-VAD vs. +DOX Z-VAD	0.4995	0.3323 to 0.6667	***	0.0008
Q-VD vs. +DOX Q-VD	0.398	-0.2733 to 1.069	ns	0.1348
Fe+NSA+Z-VAD vs. +DOX+Fe+NSA+Z-VAD	0.4291	-0.9058 to 1.764	ns	0.3805
Fe+NSA+Q-VD vs. +DOX+Fe+NSA+Q-VD	0.5922	-19.36 to 20.54	ns	0.8393
+DOX Vehicle vs. +DOX Fe-1 1 uM	0.026	-0.5149 to 0.5669	ns	>0.9999
+DOX Vehicle vs. +DOX NSA 100 nM	0.09371	-0.3624 to 0.5498	ns	0.9353
+DOX Vehicle vs. +DOX Z-VAD	0.08756	-0.2989 to 0.4740	ns	0.9712
+DOX Vehicle vs. +DOX Q-VD	-0.04309	-0.9830 to 0.8968	ns	>0.9999
+DOX Vehicle vs. +DOX+Fe+NSA+Z-VAD	0.2567	-1.370 to 1.883	ns	0.8308
+DOX Vehicle vs. +DOX+Fe+NSA+Q-VD	0.1572	-27.90 to 28.22	Ns	>0.9999

CDX30P

Šídák's multiple comparisons test	Mean Diff.	95.00% CI of diff.	Summary	Adjusted P Value
Fe-1 vs. +DOX Fe-1 1 uM	0.4678	-0.1816 to 1.117	ns	0.0938
NSA vs. +DOX NSA 100 nM	0.4366	-0.2933 to 1.167	ns	0.1326
Z-VAD vs. +DOX Z-VAD	0.3454	0.1647 to 0.5262	**	0.0076
Q-VD vs. +DOX Q-VD	0.3154	-0.2277 to 0.8586	ns	0.14
Fe+NSA+Z-VAD vs. +DOX+Fe+NSA+Z-VAD	0.3167	-0.7970 to 1.430	ns	0.4541
Fe+NSA+Q-VD vs. +DOX+Fe+NSA+Q-VD	0.2361	-1.405 to 1.877	ns	0.8753
+DOX Vehicle vs. +DOX Fe-1 1 uM	0.09983	-0.4938 to 0.6935	ns	0.7963
+DOX Vehicle vs. +DOX NSA 100 nM	0.1081	-0.6737 to 0.8898	ns	0.892
+DOX Vehicle vs. +DOX Z-VAD	0.0405	-0.5204 to 0.6014	ns	>0.9999
+DOX Vehicle vs. +DOX Q-VD	-0.06579	-1.843 to 1.711	ns	>0.9999
+DOX Vehicle vs. +DOX+Fe+NSA+Z-VAD	0.06443	-0.5439 to 0.6728	ns	0.967
+DOX Vehicle vs. +DOX+Fe+NSA+Q-VD	0.06814	-1.212 to 1.348	ns	0.9998

Supplemental References

1 Menendez, L. *et al.* Generation of inner ear hair cells by direct lineage conversion of primary somatic cells. *Elife* **9**, doi:10.7554/eLife.55249 (2020).

2 Starrett, G. J. *et al.* Clinical and molecular characterization of virus-positive and virus-negative Merkel cell carcinoma. *Genome Med* **12**, 30, doi:10.1186/s13073-020-00727-4 (2020).

3 Caeser, R. *et al.* Genomic and transcriptomic analysis of a library of small cell lung cancer patient-derived xenografts. *Nat Commun* **13**, 2144, doi:10.1038/s41467-022-29794-4 (2022).

4 Lallo A, W. B. U., Frese KK, Potter DS, Helleday T, Dive C. Ex vivo culture of circulating tumour cell derived explants to facilitate rapid therapy testing in small cell lung cancer. *European Association for Cancer Research Annual Conference. Manchester, U.K.2016* (2016).

5 Murphy, B. *et al.* Evaluation of Alternative In Vivo Drug Screening Methodology: A Single Mouse Analysis. *Cancer Res* **76**, 5798-5809, doi:10.1158/0008-5472.CAN-16-0122 (2016).

6 Cai, T. *et al.* Characterization of the transcriptome of nascent hair cells and identification of direct targets of the Atoh1 transcription factor. *J Neurosci* **35**, 5870-5883, doi:10.1523/JNEUROSCI.5083-14.2015 (2015).

7 Burns, J. C., Kelly, M. C., Hoa, M., Morell, R. J. & Kelley, M. W. Single-cell RNA-Seq resolves cellular complexity in sensory organs from the neonatal inner ear. *Nat Commun* **6**, 8557, doi:10.1038/ncomms9557 (2015).

8 Schenk, M. W. *et al.* Soluble guanylate cyclase signalling mediates etoposide resistance in progressing small cell lung cancer. *Nat Commun* **12**, 6652, doi:10.1038/s41467-021-26823-6 (2021).

9 Borromeo, M. D. *et al.* ASCL1 and NEUROD1 Reveal Heterogeneity in Pulmonary Neuroendocrine Tumors and Regulate Distinct Genetic Programs. *Cell Rep* **16**, 1259-1272, doi:10.1016/j.celrep.2016.06.081 (2016).

10 Ireland, A. S. *et al.* MYC Drives Temporal Evolution of Small Cell Lung Cancer Subtypes by Reprogramming Neuroendocrine Fate. *Cancer Cell* **38**, 60-78 e12, doi:10.1016/j.ccell.2020.05.001 (2020).
