# Supplementary material for: Functional characterization of the ATOH1 molecular subtype indicates a pro-metastatic role in small cell lung cancer
Source: Cell Rep. 2025 Apr 29;44(5):115603. doi: 10.1016/j.celrep.2025.115603 (PMC12116416; doi:10.1016/j.celrep.2025.115603)

Data S1 (relative to Figure 3B). Detection of ATOH1 and Lamin B in nuclear and cytoplasmic fractions of CDX17P with and without ATOH1 knockdown.

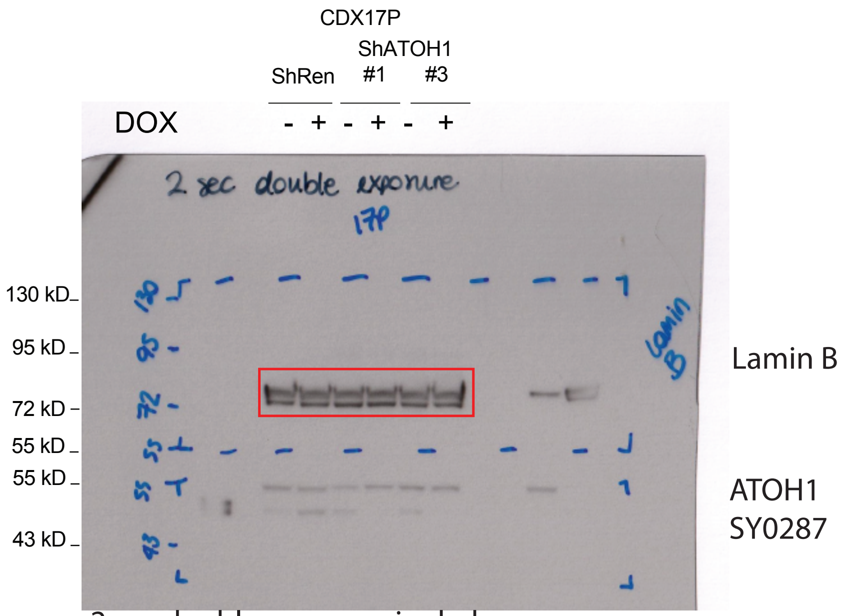

2 sec double exposure in dark room

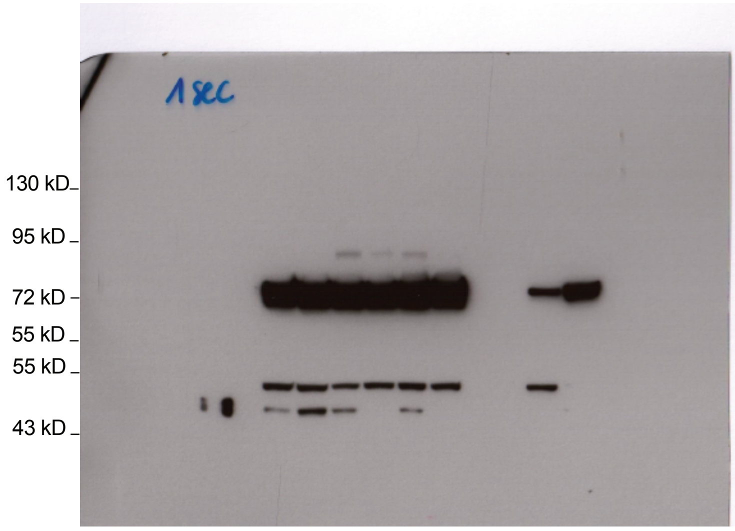

1 sec exposure in dark room

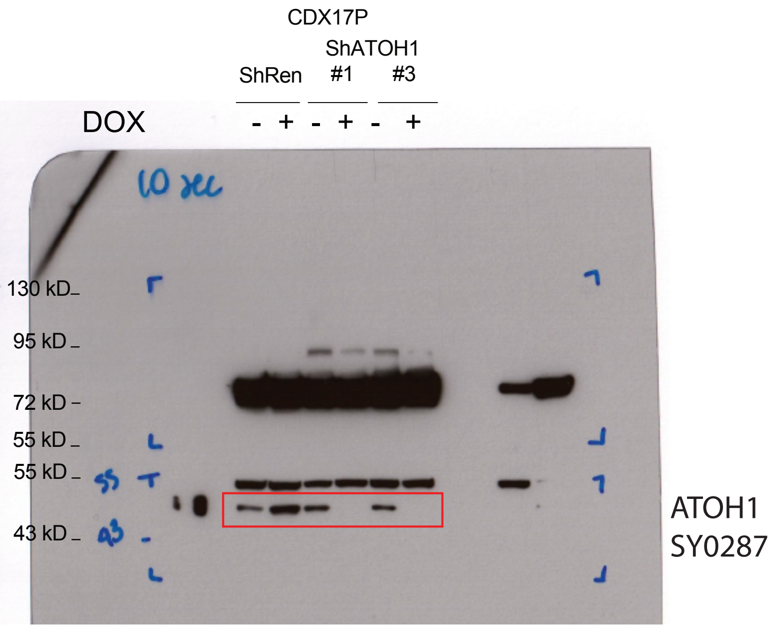

10 sec exposure in dark room

Data S2 (relative to Figure 3C). Detection of ATOH1 in CDX17P with or without ATOH1 knockdown subjected to ChIPSeq.

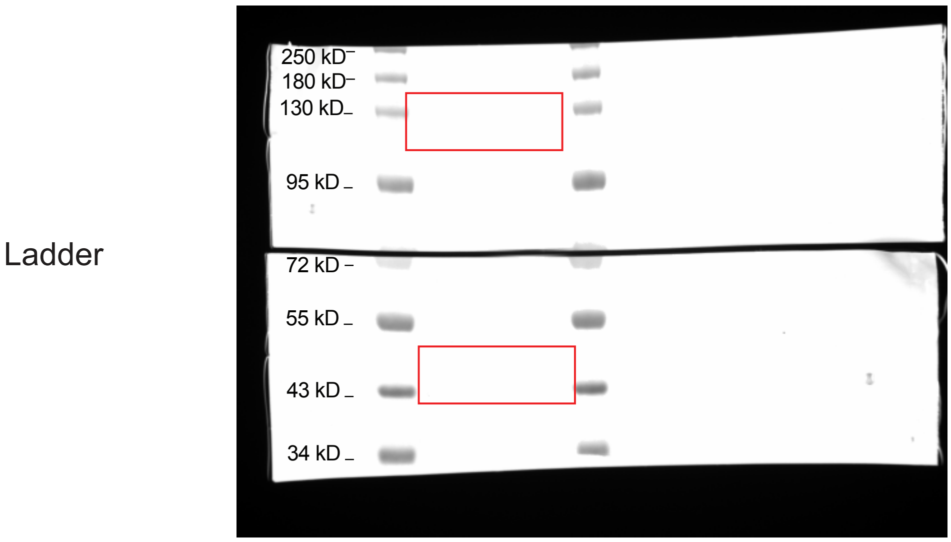

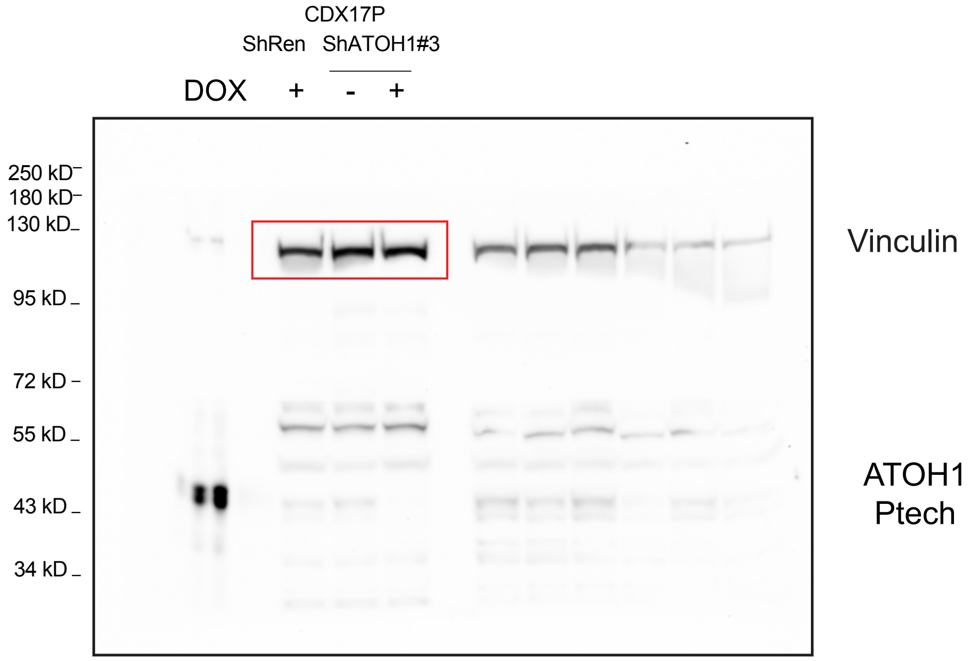

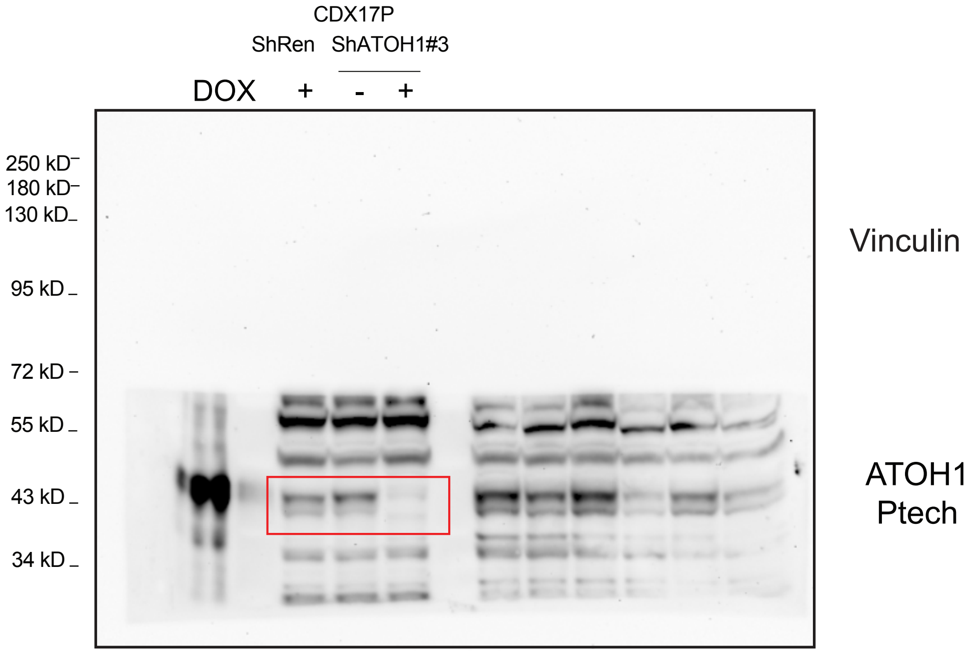

Data S3 (relative to Figure 5B). Detection of ATOH1 in CDX17P with or without ATOH1 knockdown and restoration after removal of doxycyline.

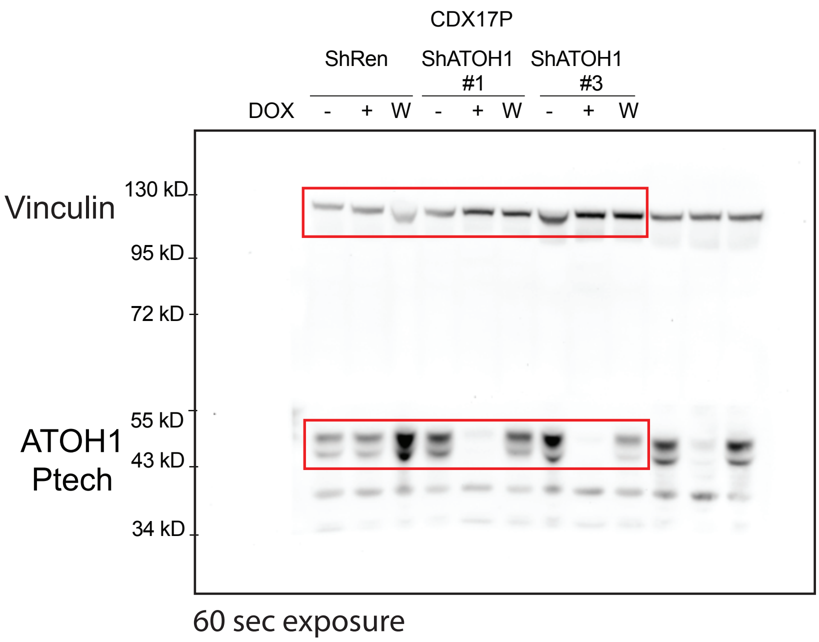

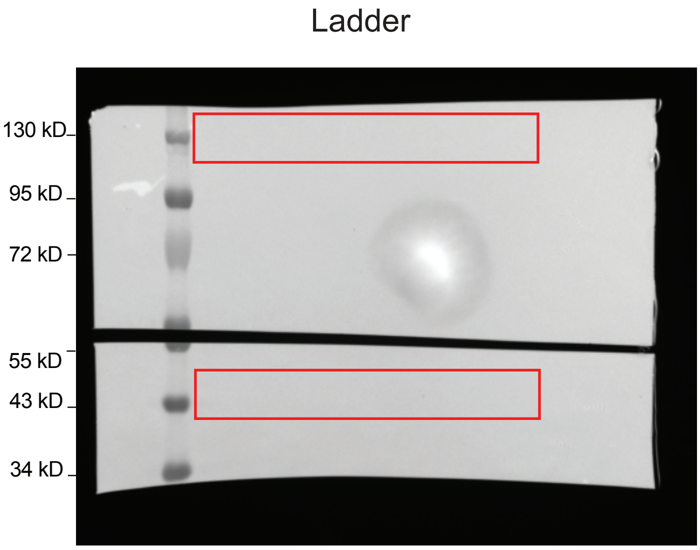

Data S4 (relative to figure S2A). Detection of ATOH1 in CDX17P ShRen upon treatment with doxycyline for 2,4 or 7 days.

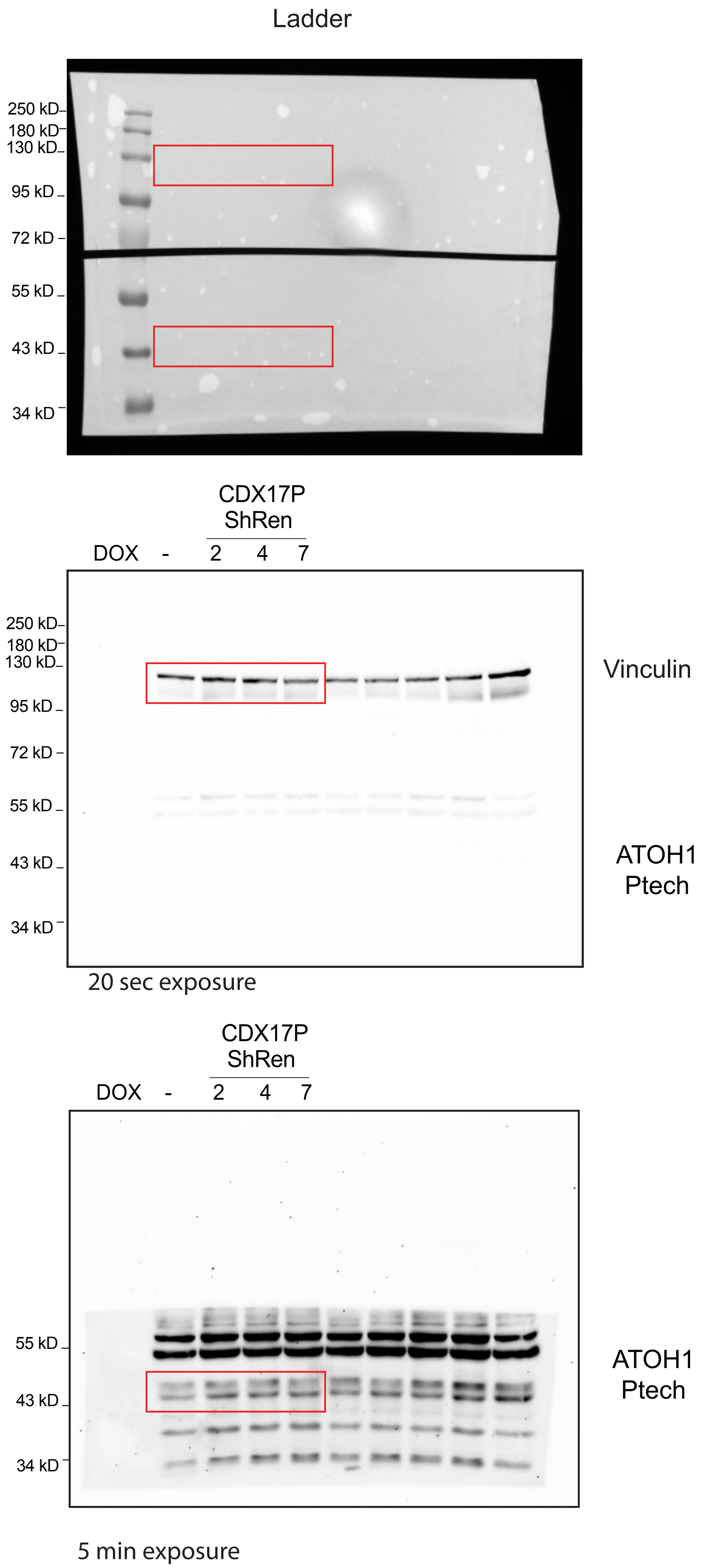

Data S5 (relative to figure S2A). Detection of ATOH1 in CDX17P ShATOH1#1 upon treatment with doxycyline for 2,4 or 7 days.

Ladder

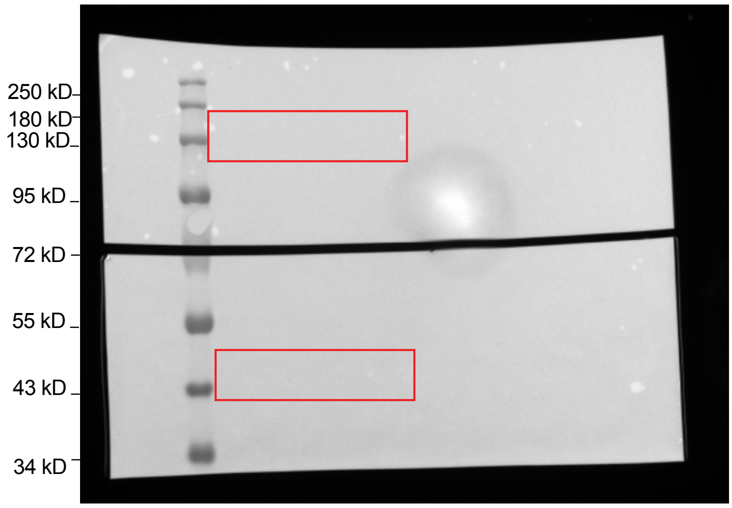

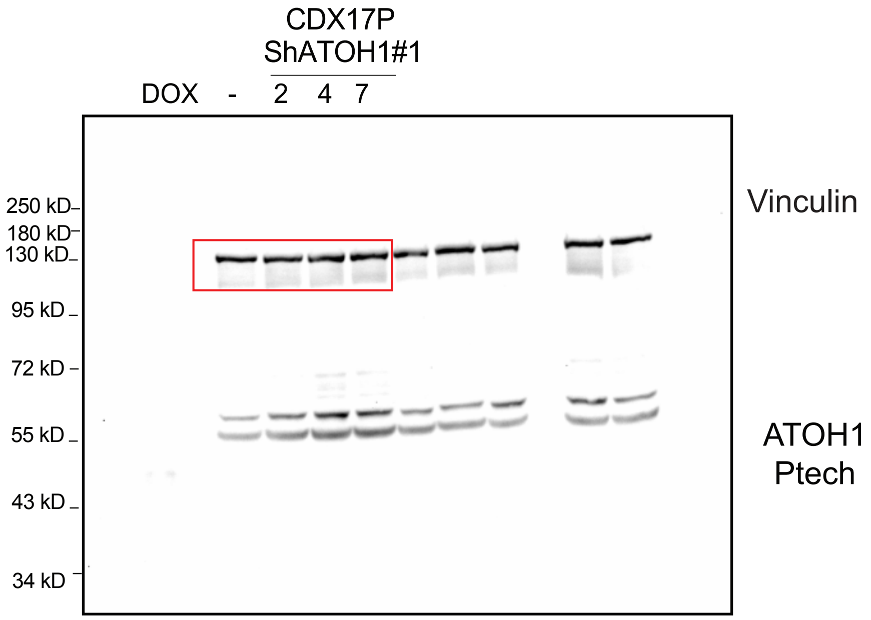

20 sec exposure

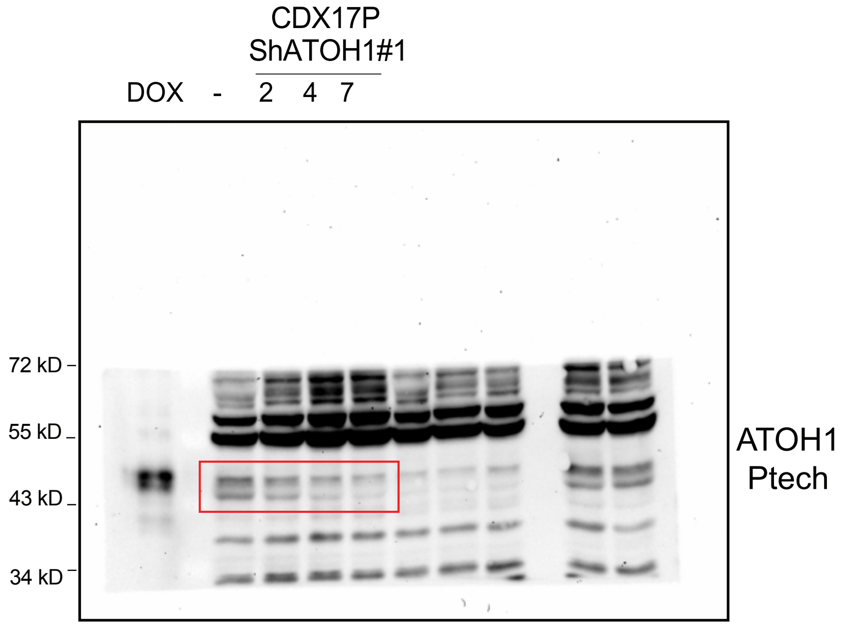

5 min exposure

Data S6 (relative to figure S2A). Detection of ATOH1 in CDX17P ShATOH1#3 upon treatment with doxycyline for 2,4 or 7 days.

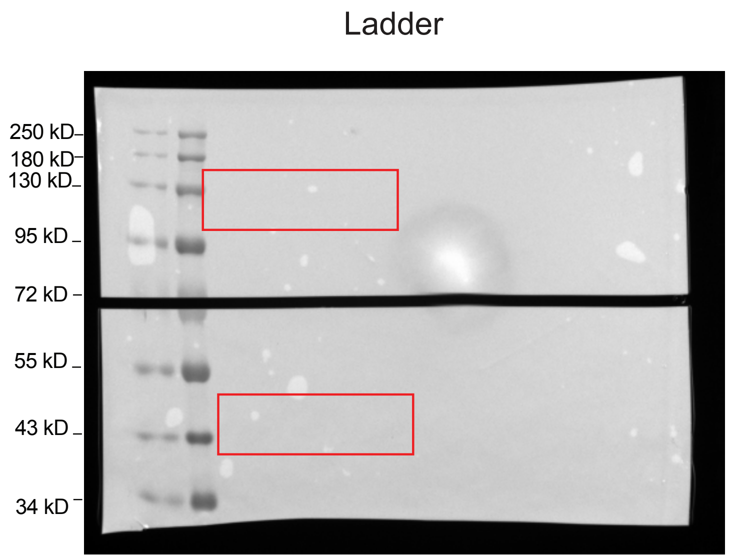

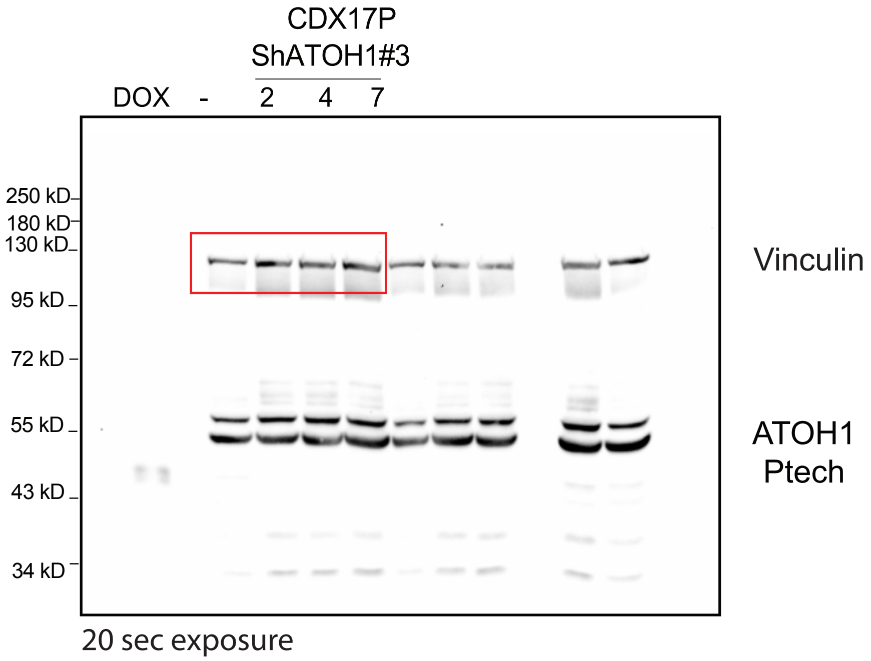

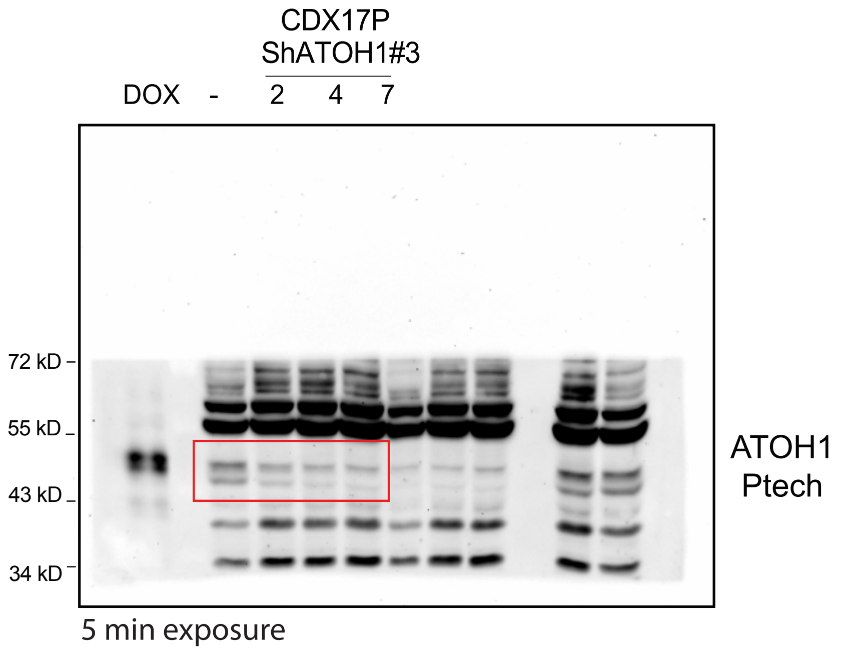

Data S7 (relative to Figure S2D). Detection of ATOH1 recombinant protein with SY0287 serum.

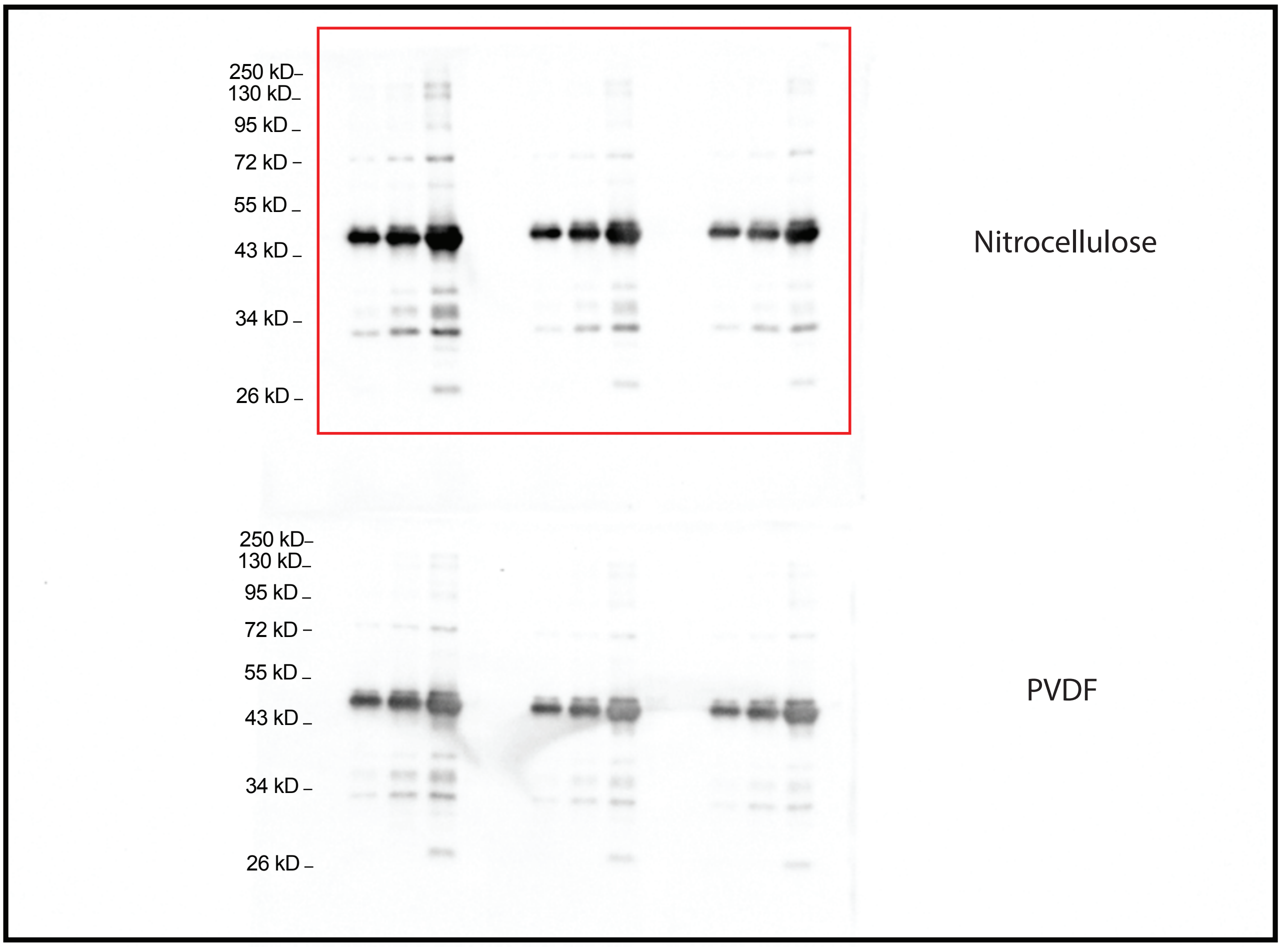

5 sec exposure

Ladder

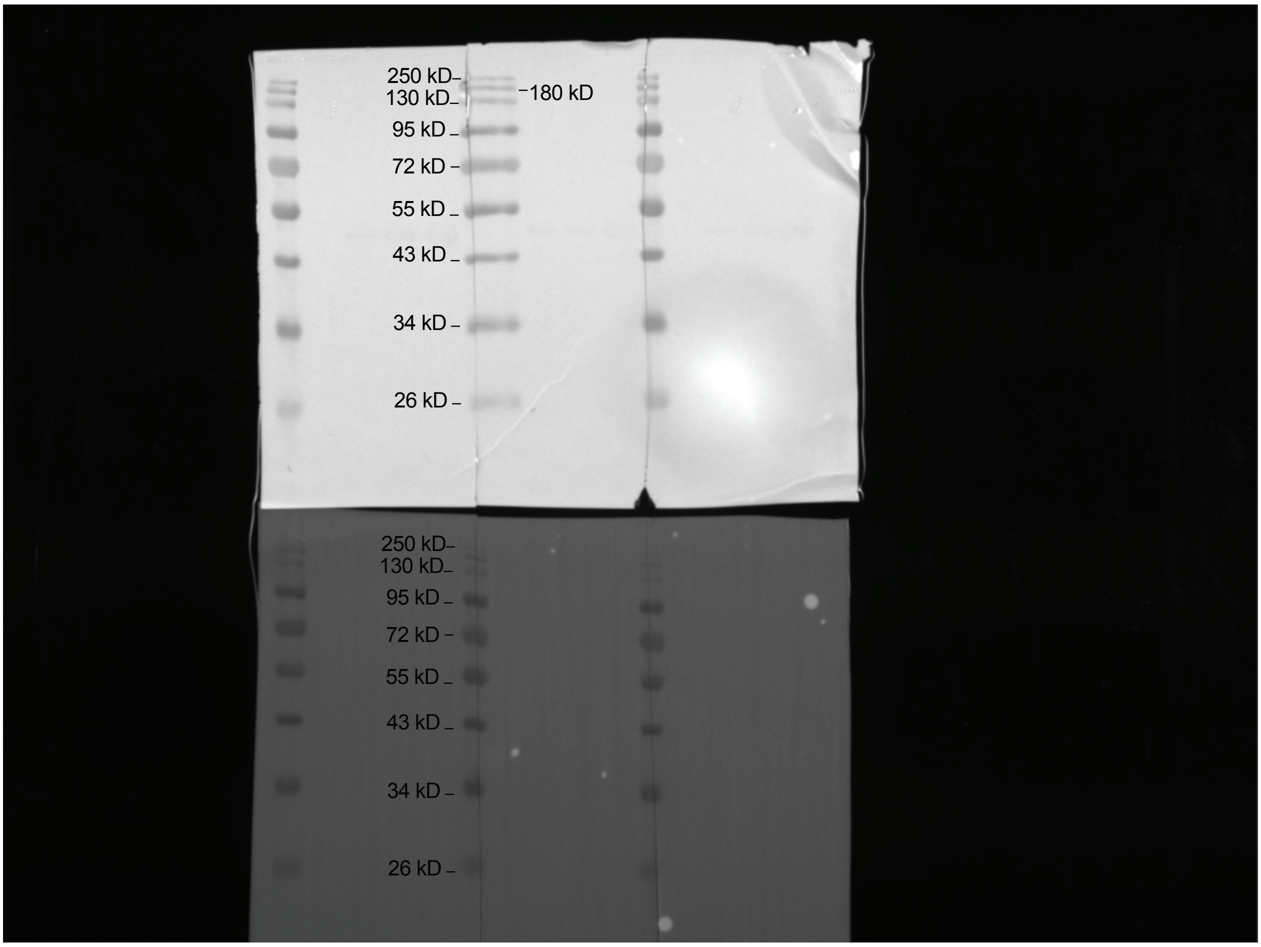

Data S8 (relative to Figure S2E). Detection of ATOH1 in nuclear fractions of CDX30P and CDX17 Non-Neuroendocrine (NNE) cells.

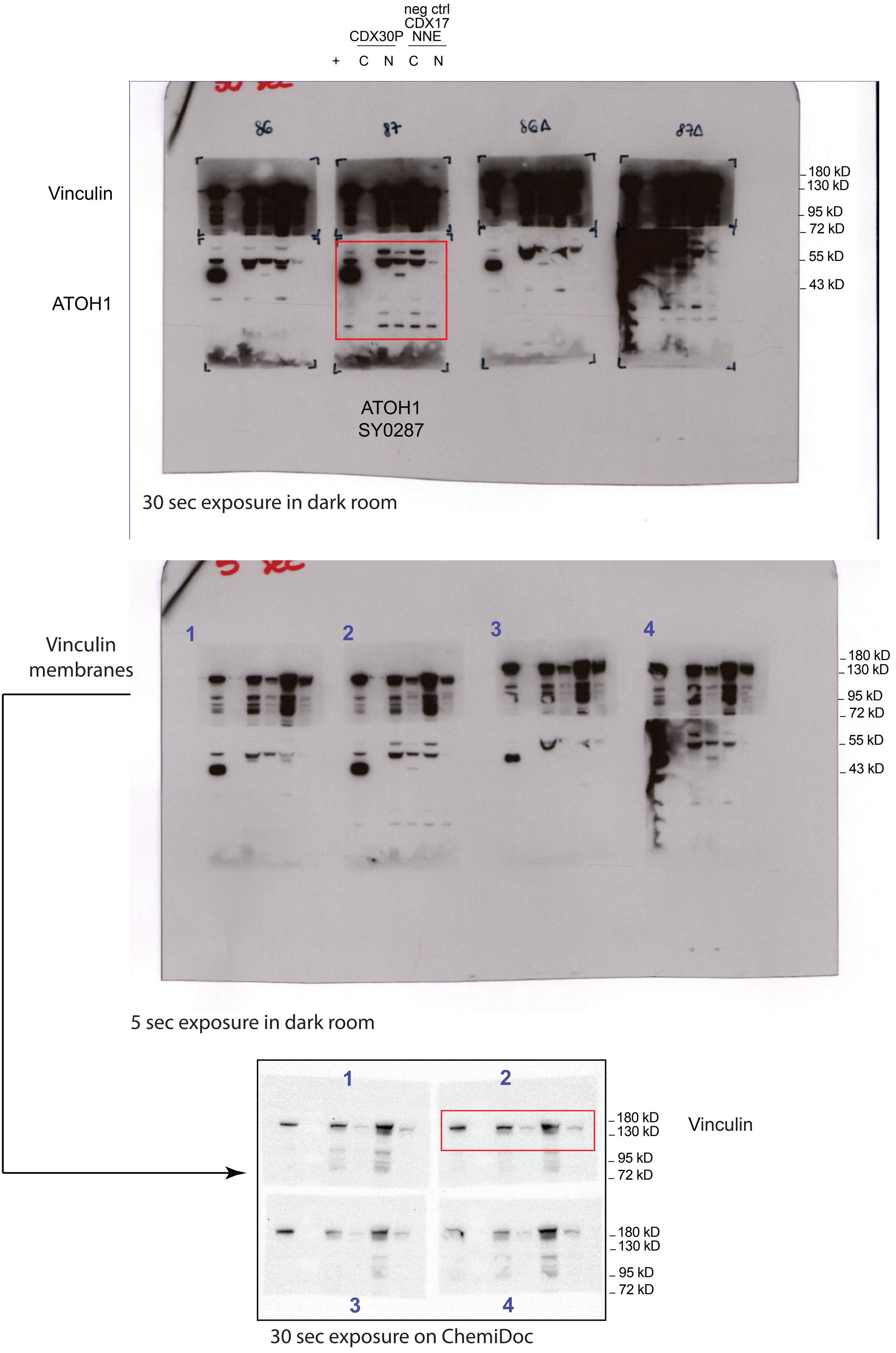

Data S9 (relative to Figure S4E). Detection of neuroendocrine and non-neuroendocrine markers in CDX17P with or without ATOH1 knockdown.

Gel 1

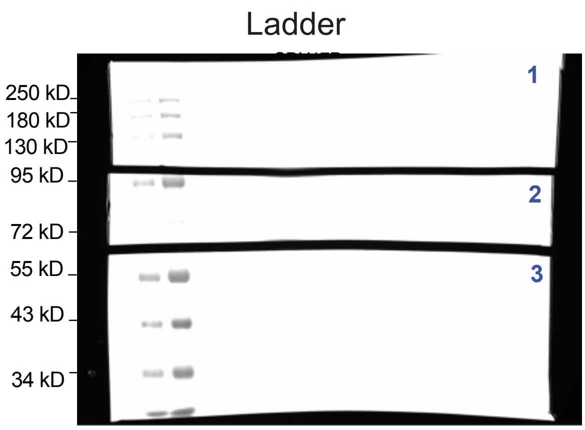

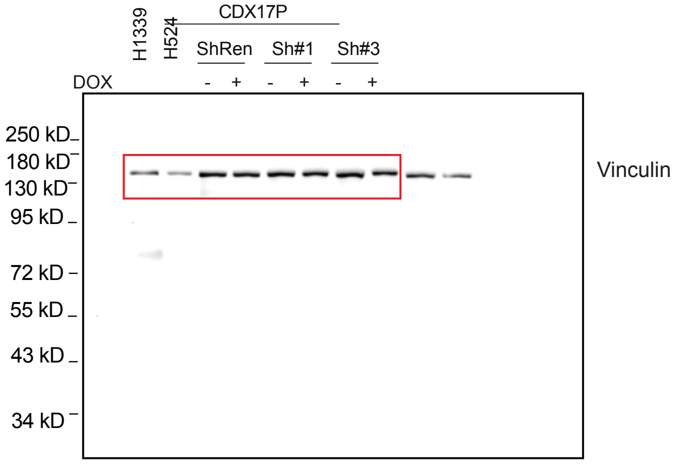

30 sec exposure on ChemiDoc

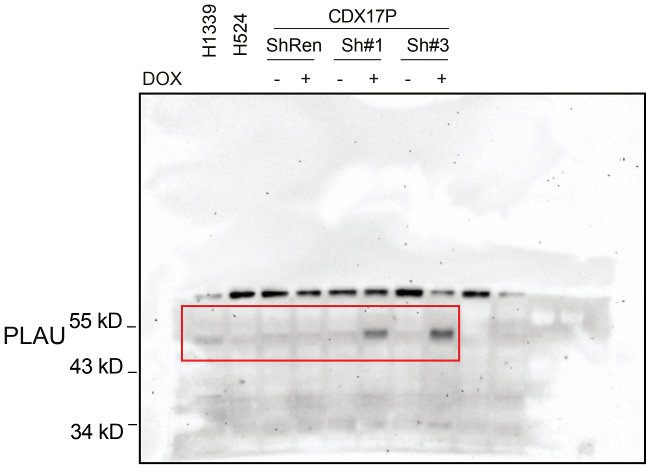

180 sec exposure on ChemiDoc only bottom membrane

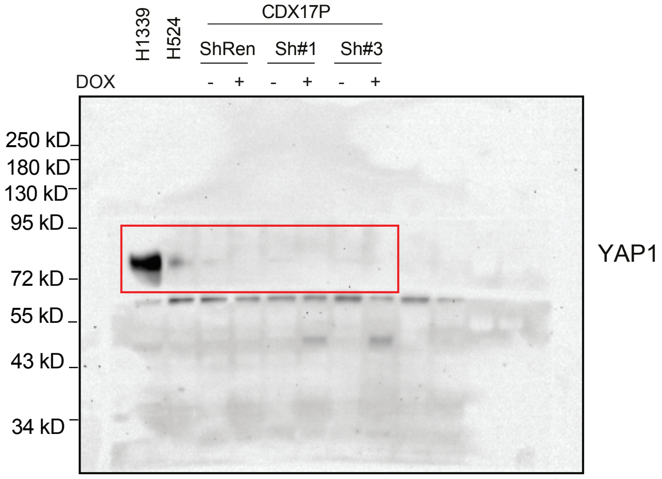

90 sec exposure on ChemiDoc without vinculin membrane

Gel 2

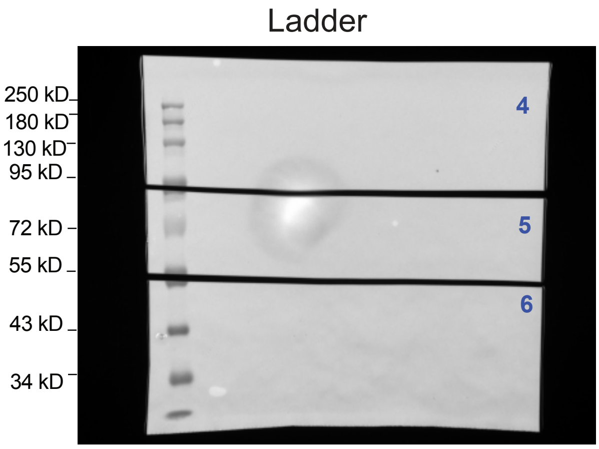

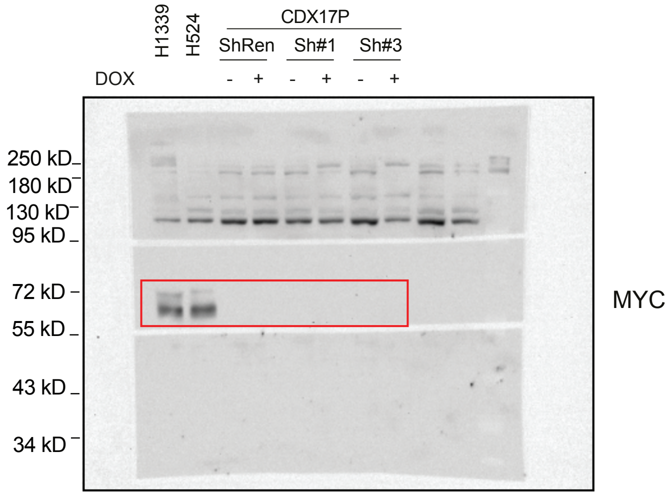

30 sec exposure on ChemiDoc

Data S9 (relative to Figure S4E). Continued.

Gel 1 top stripped for SYP, Gel 2 stripped for NEUROD1 and VINCULIN

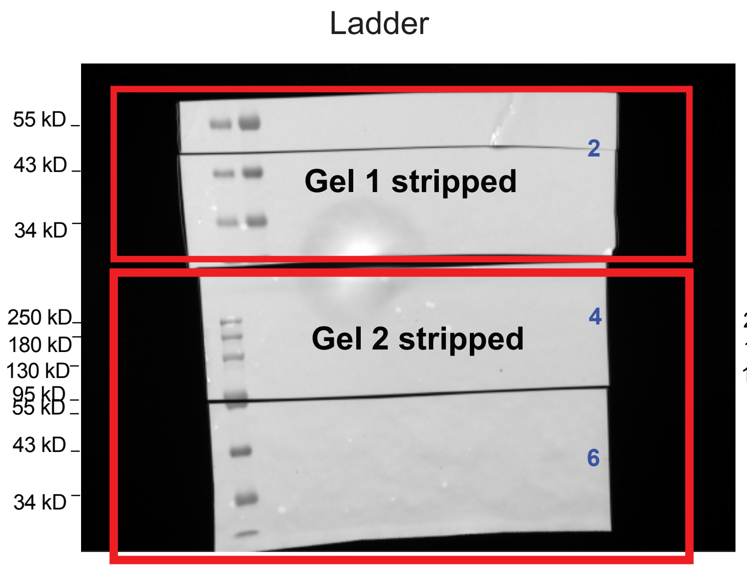

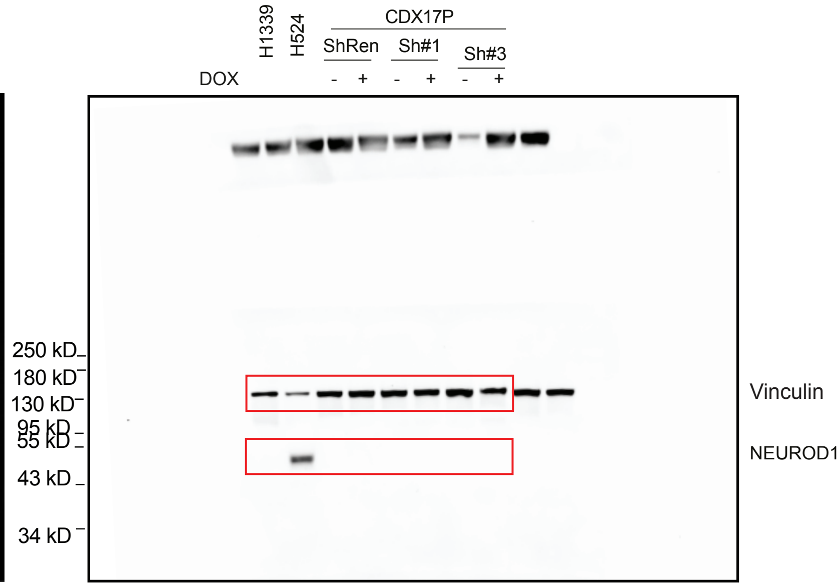

90 sec exposure on ChemiDoc

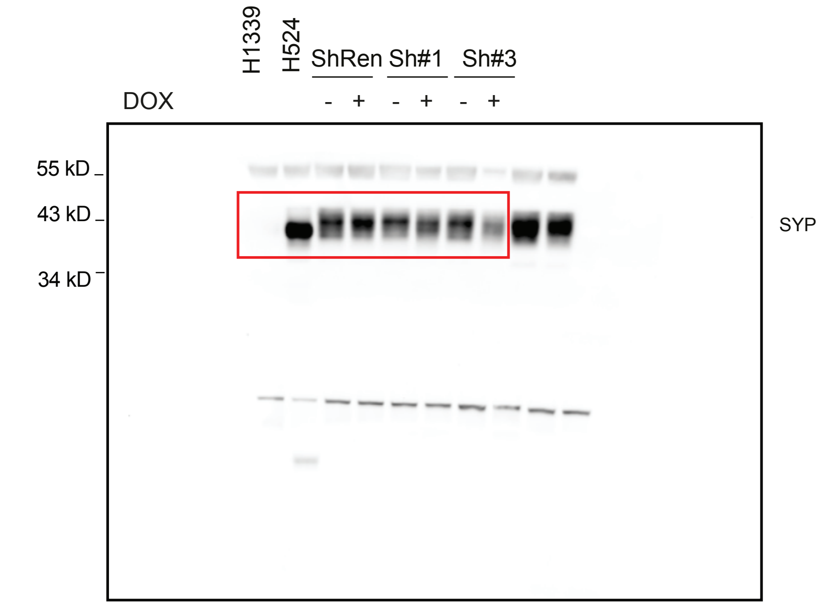

20 sec exposure on ChemiDoc

Data S10 (relative to Figure S5A). Detection of ATOH1 in CDX30P with or without ATOH1 knockdown.

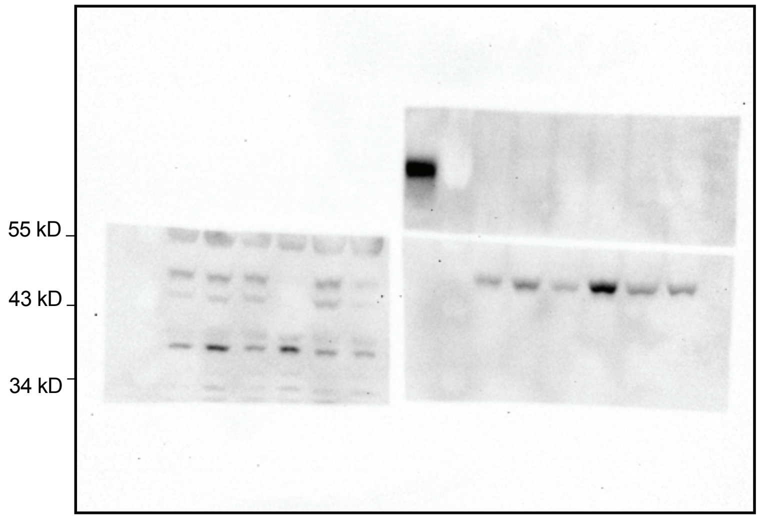

Ladder

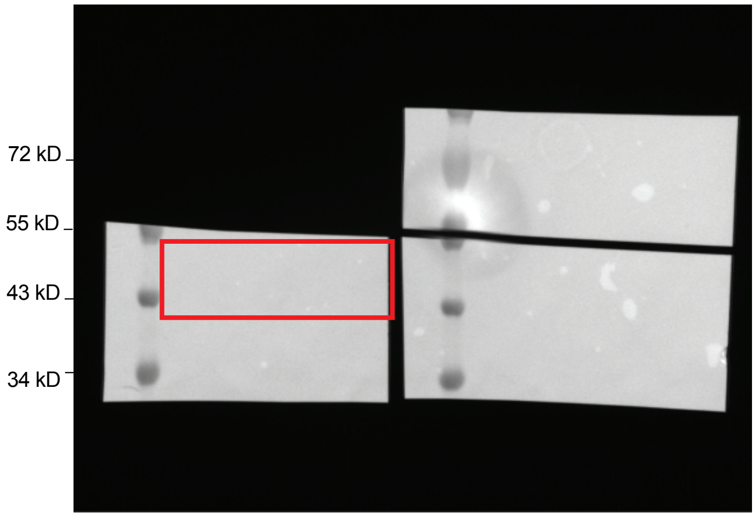

30 sec exposure

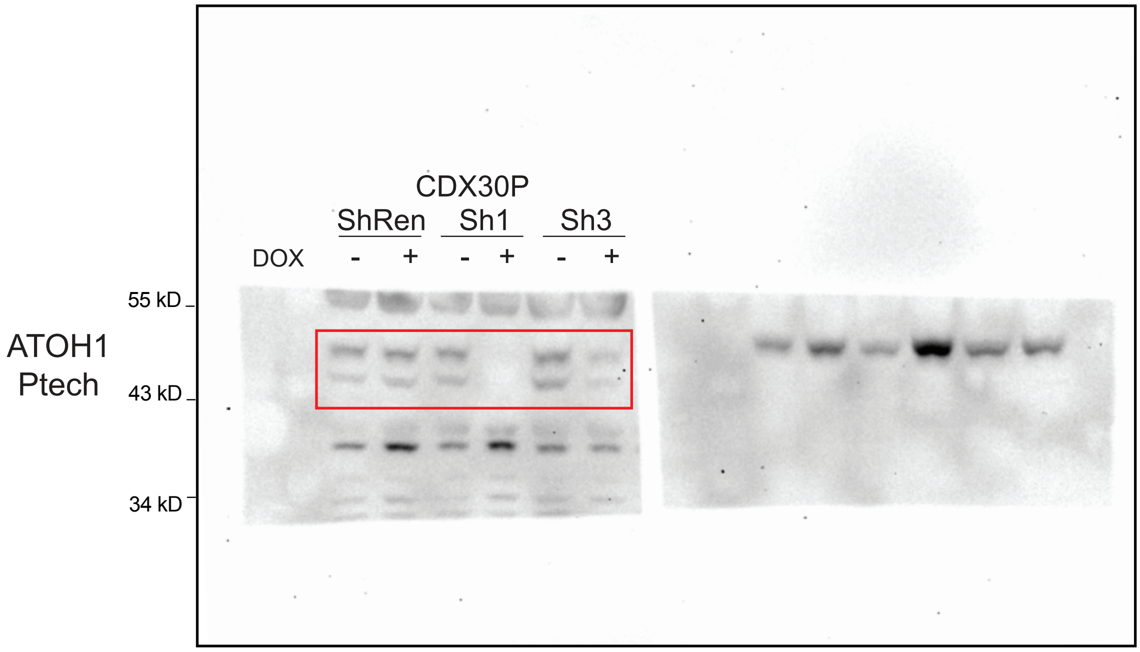

60 sec exposure without top right membrane

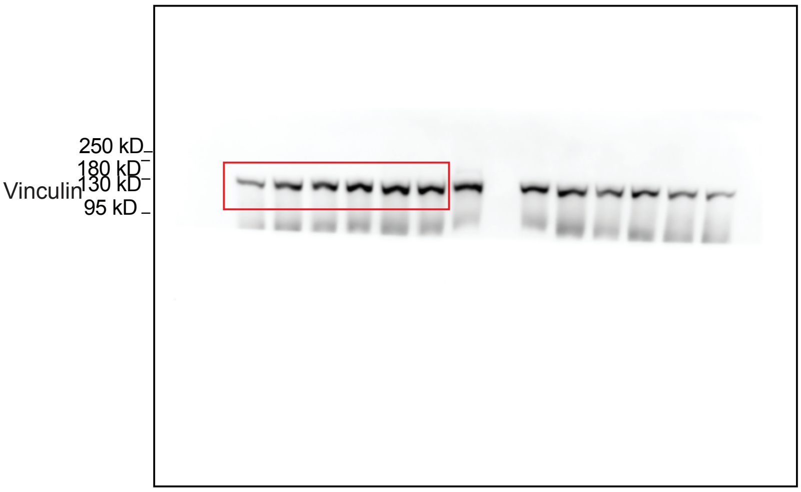

Ladder

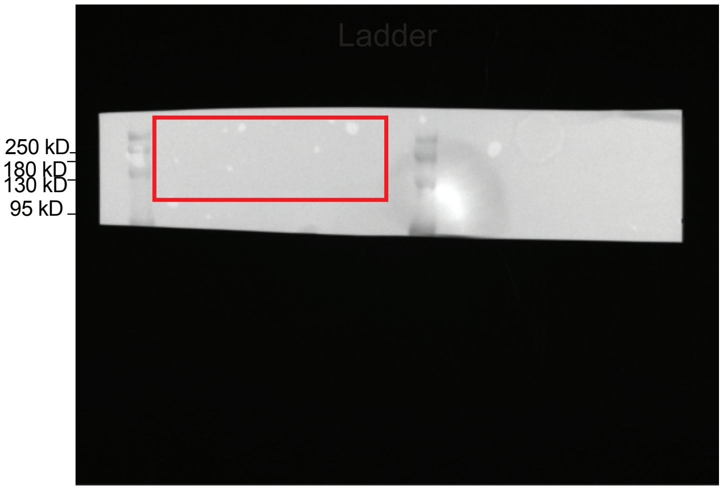

10 sec exposure

Data S10 (relative to Figure S5B). Detection of ATOH1 in HCC33 with or without ATOH1 knockdown.

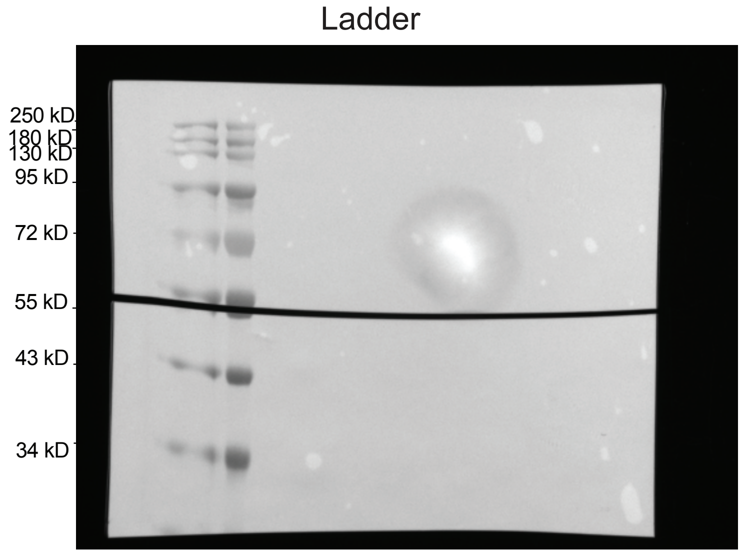

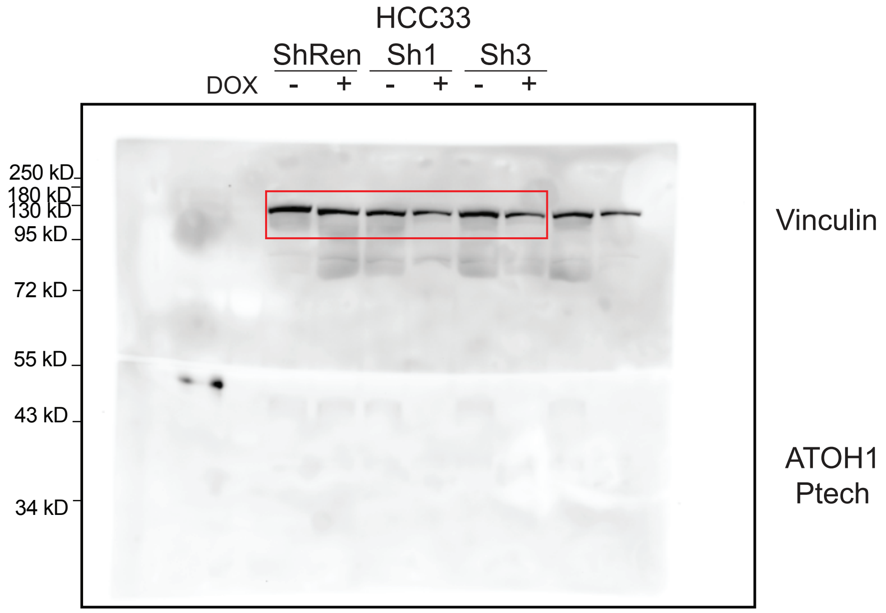

10 sec exposure

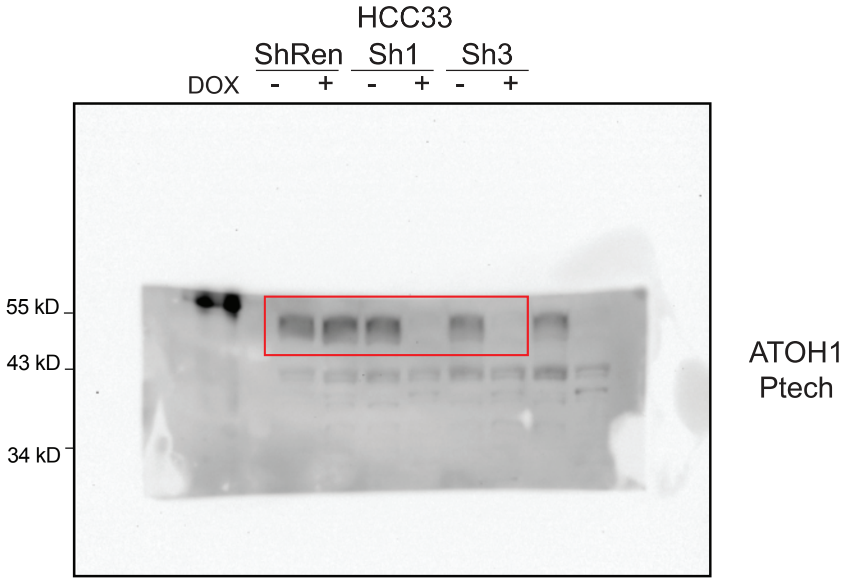

60 sec exposure without Vinculin membrane

Data S10 (relative to Figure S5E). Detection of NERUOD1 in CDX30P with or without ATOH1 knockdown.

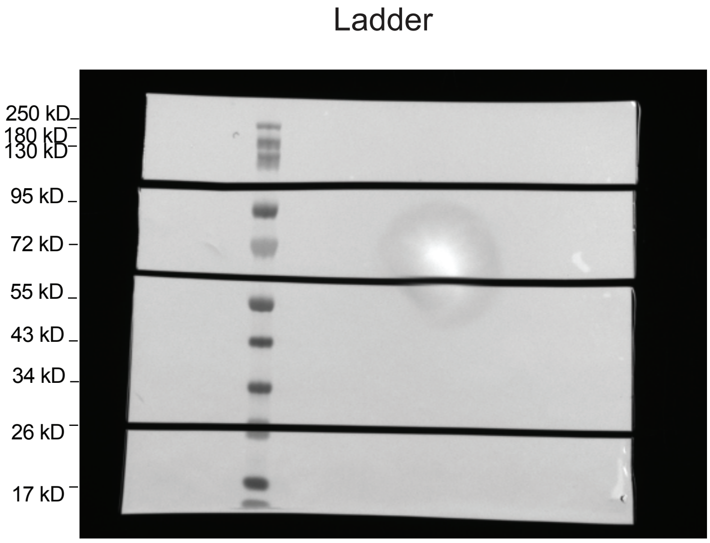

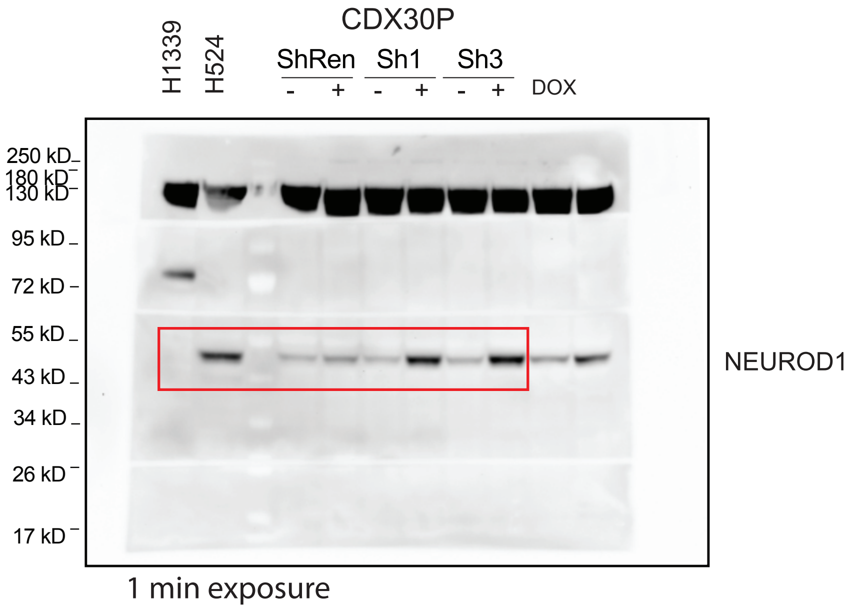

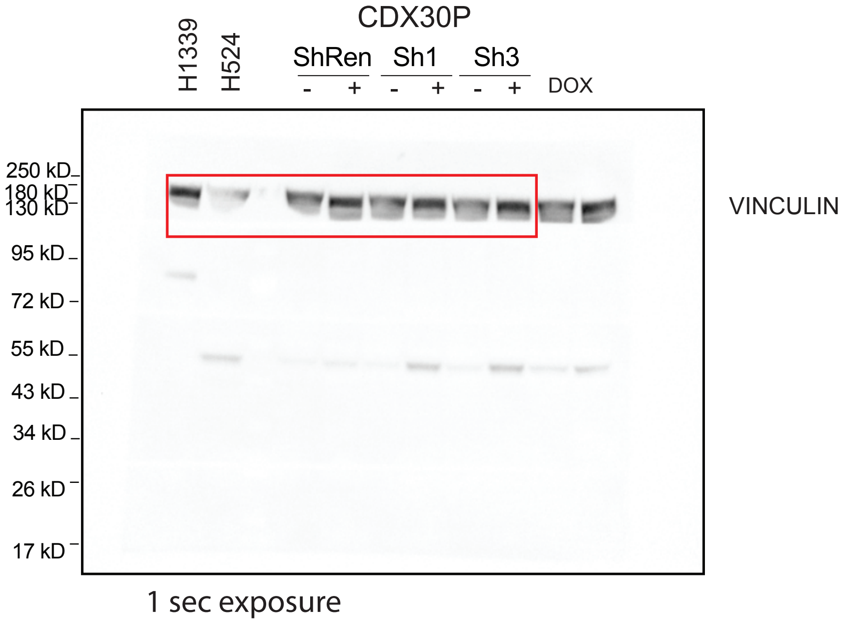

Supplement: Document S2. Data S1–S10 [file mmc2.pdf]
